# Azasulfurylpeptide Modulation of CD36-Mediated Inflammation Without Effect on Neovascularization

**DOI:** 10.3390/biomedicines6040098

**Published:** 2018-10-22

**Authors:** Stéphane Turcotte, Katia Mellal, Ramesh Chingle, Mukandila Mulumba, Samy Omri, Lylia Dif-Yaiche, Sylvain Chemtob, Huy Ong, William D. Lubell

**Affiliations:** 1 Departments of Chemistry, Université de Montréal, Montréal, QC H3C 3J7, Canada; stephane.turcotte@intellisynrd.com (S.T.); rameshchingle26@gmail.com (R.C.); dif.yaiche.lylia@gmail.com (L.D.-Y.); 2 Faculty of Pharmacy, Université de Montréal, Montréal, QC H3C 3J7, Canada; katia.mellal@gmail.com (K.M.); dilan.mulumba@gmail.com (M.M.); huy.ong@umontreal.ca (H.O.); 3 Maisonneuve-Rosemont Hospital, Montréal, QC H1T 2M4, Canada; samy.omri@gmail.com (S.O.); sylvain.chemtob@gmail.com (S.C.); 4 Pediatrics, Ophthalmology and Pharmacology, Université de Montréal, Montréal, QC H3C 3J7, Canada

**Keywords:** age-related macular degeneration, azapeptide, azasulfurylpeptide, cluster of differentiation 36, fibroblast-stimulating lipopeptide, growth hormone-releasing peptide-6, *N*-Aminosulfamide, nitric oxide, semicarbazide, toll-like receptor, tumor necrosis factor-α

## Abstract

Modulation of the cluster of differentiation-36 receptor (CD36) has proven promising for dampening pro-inflammatory macrophage signaling. For example, azapeptides (e.g., **1** and **2**) bind CD36 selectively with high affinity, mitigate Toll-like receptor (TLR) agonist-induced overproduction of nitric oxide (NO), and reduce pro-inflammatory cytokine and chemokine production in macrophages. Moreover, semicarbazides **1** and **2** inhibit microvascular sprouting mediated through CD36 in the choroid explant. Seeking a selective CD36 modulator that mediated inflammation without influencing neovascularization, a set of azasulfurylpeptides (e.g., **3a**–**e**) were synthesized in which the semicarbazide was replaced by an *N*-aminosulfamide residue using a novel solid-phase approach. Notably, azasulfurylpeptide **3c** diminished selectively CD36-mediated TLR-2-triggered inflammatory response without affecting neovascularization. Subtle chemical modification at the peptide backbone from a carbonyl to a sulfuryl residue has had a selective effect on biological activity providing a valuable probe for studying CD36 chemical biology.

## 1. Introduction

Inflammation is a defensive biological response to dangerous stimuli such as pathogens and damaged-associated molecular patterns released by apoptotic cells. In response to the tissue insult, innate immunity cells such as macrophages and microglia function to initiate inflammatory responses to remove debris by phagocytosis and to remodel the tissue to a homeostasis state [1,2,3]. Playing pivotal roles in innate immunity, macrophages protect against infection and remodel tissue mediating inflammation in disease [1,2,3,4,5,6]. Their Toll-like receptors (TLRs) recognize pathogen- and damage-associated molecular patterns, and can initiate signaling leading to inflammatory responses [7]. Macrophage signaling has emerged as a promising target for countering chronic inflammation underlying disease.

Expressed on macrophage plasma membranes, the cluster of differentiation 36 receptor (CD36) serves key roles in recognition and phagocytosis as a scavenger of multiple-ligands: e.g., oxidized lipoproteins, and cholesterol [8,9,10,11]. As a co-receptor of the TLR-2 heterodimer complex, CD36 plays a mediating role in innate immunity [12]. For example, CD36 has been shown to sustain TLR2 signaling elicited by diacylglycerols, and to regulate TLR2-dependent macrophage driven inflammation, via upregulation of NF-κB [13,14]. Modulation of CD36 may thus offer means to regulate macrophage-driven inflammation to treat disease [15,16,17]. 

**Figure 1:**
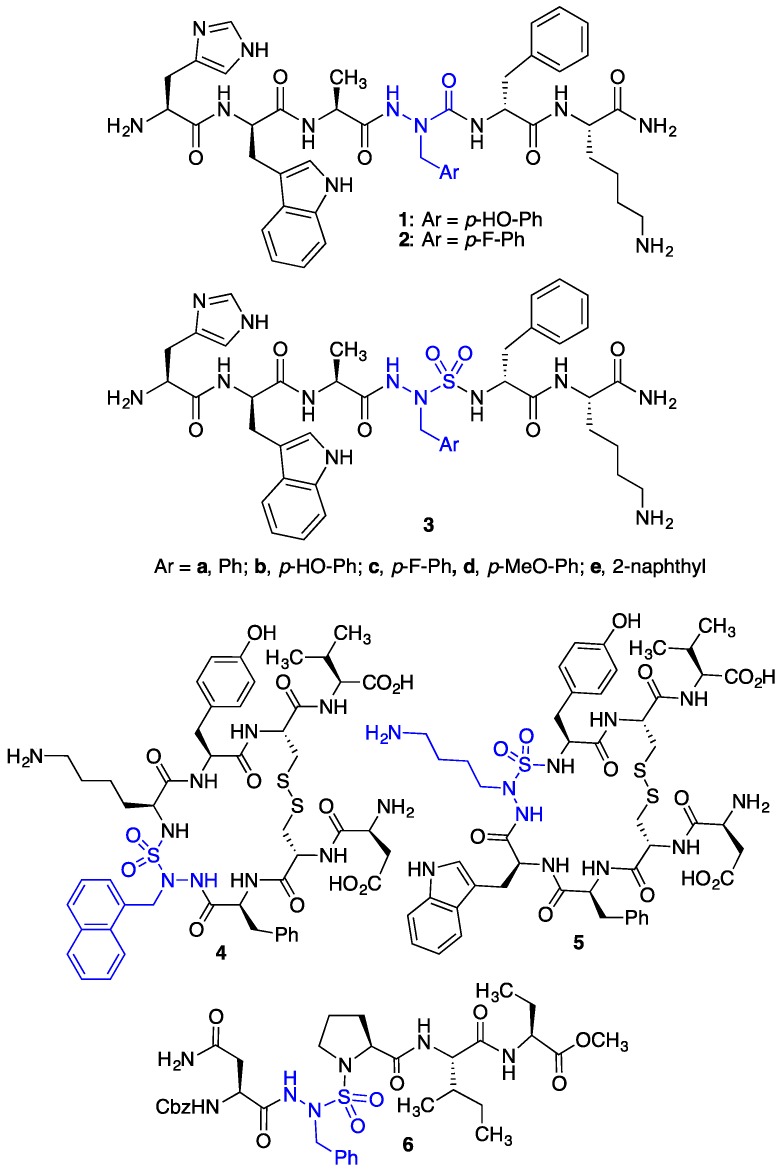
Azapeptides **1** and **2**, and azasulfurylpeptides **3**–**6**.

Semicarbazide analogs of growth hormone releasing peptide-6 (GHRP-6, H-His-D-Trp-Ala-Trp-D-Phe-Lys-NH_2_), such as [azaY^4^]- and [aza(4-F)F^4^]-GHRP-6 (**1** and **2**, Figure 1), have exhibited selectivity and relatively high binding affinity for CD36 (IC_50_ 1.90 and 6.06 µM) [15,18]. In macrophage cells, azapeptides **1** and **2** reduced significantly CD36-mediated overproduction of nitric oxide (NO) after treatment with the TLR-2-agonist fibroblast-stimulating lipopeptide (R-FSL-1) [15]. Furthermore, they attenuated induced neovascularization relative to controls in a microvascular sprouting assay on mouse choroidal explants [15]. Mediating two of its pleotropic effects, azapeptides **1** and **2** are promising lead CD36 modulators for the development of prototypes to treat disease states implicating chronic inflammation and neovascularization. For example, in *ApoE*^−/−^ mice prone to accumulate lipids and remnants in the Bruch membrane characteristic of the early phase of age-related macular degeneration (AMD) [19], [azaY^4^]-GHRP-6 decreased sub-retinal deposits and minimized macrophage infiltration within the subretinal space. In the interest to differentiate the roles of CD36 in inflammatory response and neovascularization, a search for modulators that influence only the former has been pursued.

Azasulfurylpeptides (e.g., **3**–**6**, Figure 1) are peptides containing an *N*-aminosulfamide as amino amide surrogate [20,21,22,23,24,25]. Combining semicarbazide [26] and sulfonamide properties [27,28], *N*-aminosulfamides were shown (by X-ray crystallography) to restrict model peptide backbones to geometry characteristic of the central residues in ideal β- and γ-turns [22]. Cyclic azasulfurylpeptides have served as ligands of the urotensin II receptor (e.g., **4** and **5**) [23]. Moreover, the sulfamide tetrahedral geometry was credited for the activity of azasulfurylpeptide **6**, which served as a transition-state mimic inhibitor of human immunodeficiency virus-1 (HIV-1) proteinase [24,25]. 

Relative to amino amides, semicarbazides and *N*-aminosulfamides are expected to exhibit enhanced metabolic stability [26]. Moreover, the latter may have an improved potential to make backbone hydrogen bonds due to greater NH Brønsted acidity and sulfuryl oxygen Lewis basicity [22]. Although azasulfurylpeptide synthesis has historically impeded their broad application, a novel solution-phase method featuring alkylation of an azasulfurylglycine (AsG) tripeptide has recently enabled installment of side-chains onto the *N*-aminosulfamide residues [20,21]. Towards the development of a diversity-oriented approach to generate libraries for studying structure–activity relationships, solid-phase methods are now reported for making azasulfurylpeptides and used to explore the impact in replacing a semicarbazide by an *N*-aminosulfamide residue in azapeptide ligands (e.g., **1** and **2**). 

Through the exploration of the biological activities of azasulfurylpeptides, a novel class of CD36 modulators has been developed and epitomized by azasulfuryl-*p*-fluorophenylalanine-4- [As(4-F)F^4^]-GHRP-6 (**3c**). Exhibiting similar binding affinity as its aza-counterpart, [aza(4-F)F^4^]-GHRP-6 (**2**), azasulfuryl analog **3c** reduced CD36-mediated overproduction of NO, and pro-inflammatory chemokines and cytokines in macrophage cells. Moreover, in a murine model, azasulfurylpeptide **3c** behaved like azapeptides **1** and **2** and reduced recruitment of subretinal IBA-1 positive cells induced by blue light exposure. In contrast to the azapeptides **1** and **2**, which reduced angiogenic sprouting in mouse choroidal explants, azasulfurylpeptide **3c** did not affect choroidal vascular growth. A selective modulator of inflammation without influence on neovascularization, [As(4-F)F^4^]-GHRP-6 (**3c**) represents a novel probe for differentiating the pleotropic effects of CD36 towards the development of therapeutic prototypes for countering chronic inflammation without effect on the microvasculature.

## 2. Experimental Section

### 2.1. N-(Boc)Alaninyl-azasulfurylphenylalaninyl-D-phenylalanine tert-butyl ester *(**8**)*

A solution of Boc-Ala-AsG-D-Phe-O*t*-Bu (**7**, 243 mg, 0.50 mmol, prepared according to [20]) in THF (5 mL) at room temperature (rt) was treated with tetraethylammonium hydroxide (40% in H_2_O, 202 μL, 0.55 mmol) and benzyl bromide (66 μL, 0.55 mmol), and stirred for 3h. The volatiles were evaporated to a residue, which was dissolved in dichloromethane (DCM, 10 mL), washed with 5% citric acid (1 × 10 mL), water (1 × 10 mL) and brine (1 × 10 mL), dried over MgSO_4_, filtered and evaporated. The residue was purified by flash chromatography [29] eluting with hexane:EtOAc 3:1 to afford AsF-peptide **8** as a solid (235 mg, 81%): R*_f_* 0.42 (hexane:EtOAc 7:3); mp 60 °C; [α]${}_{D}^{20}$ −57.6° (CHCl_3_, *c* 0.96); ^1^H NMR (CDCl_3_, 400 MHz) 1.14 (3H, d, *J* = 7.0), 1.39 (9H, s), 1.40 (9H, s), 3.14 (2H, d, *J* = 5.7), 3.95–4.05 (1H, m), 4.46 (1H, q, *J* = 5.8), 4.50 (1H, d, *J* = 14.2), 4.55 (1H, d, *J* = 14.1), 4.90 (1H, d, *J* = 7.2), 5.61 (1H, d, *J* = 6.8), 7.20–7.35 (10H, m), 7.88 (1H, s); ^13^C NMR (CDCl_3_, 100 MHz) 18.0, 28.3, 28.6, 39.0, 49.2, 55.2, 57.7, 80.9, 83.2, 127.3, 128.5, 128.6, 128.9, 129.6, 130.3, 134.8, 136.2, 156.0, 170.5, 172.1; IR (neat) *v*_max_/cm^−1^ 1155, 1253, 1369, 1460, 1501, 1693, 2989; HRMS (ESI) *m*/*z* calculated for C_28_H_40_N_4_NaO_7_S [M + Na]^+^ 599.2510; found 599.2526.

### 2.2. N-(Fmoc)Alaninyl-azasulfurylphenylalaninyl-D-phenylalanine tert-butyl ester *(**9**)*

A solution of AsF-peptide **8** (202 mg, 0.35 mmol) in *t*-BuOAc (1.00 mL) was treated with conc. H_2_SO_4_ (77.8 μL, 1.40 mmol), stirred at rt for 3 h, quenched with saturated NaHCO_3_ (10 mL), and extracted with EtOAc (4 × 15 mL). The organic layers were combined and evaporated to a residue, which was dissolved in water (3.5 mL), cooled in an ice bath, and treated with potassium carbonate (65 mg, 0.47 mmol) followed drop-wise with a solution of 9-fluorenylmethyl N-succinimidyl carbonate (Fmoc-OSu, 138 mg, 0.14 mmol) in 1,4-dioxane (6.5 mL). After stirring at 0 °C for 1 h and at rt for 4 h, brine was added (25 mL) to the mixture. The layers were separated and the aqueous phase was extracted with EtOAc (3 × 40 mL). The organic phases were combined, dried over MgSO_4_, filtered and evaporated. The residue was purified by flash chromatography [29] eluting with hexane:EtOAc 7:3 to afford carbamate **9** as a solid (123 mg, 53%): R*_f_* 0.33 (hexane:EtOAc 7:3); mp 77 °C; [α]${}_{D}^{20}$ −38.5° (CHCl_3_, *c* 1.00); ^1^H NMR (CDCl_3_, 400 MHz) δ 1.16 (3H, d, *J* = 6.2), 1.41 (9H, s), 3.10–3.15 (1H, m), 3.15–3.20 (1H, m), 4.05–4.10 (1H, m), 4.15 (1H, t, *J* = 6.4), 4.25–4.35 (2H, m), 4.45–4.65 (3H, m), 5.29 (1H, d, *J* = 6.9), 5.75 (1H, d, *J* = 6.6), 7.20–7.35 (12H, m), 7.40 (2H, t, *J* = 7.2), 7.50–7.60 (2H, m), 7.76 (2H, d, *J* = 7.6), 8.00 (1H, s); ^13^C NMR (CDCl_3_, 100 MHz) δ 18.3, 28.2, 39.1, 47.3, 49.4, 55.3, 57.6, 67.6, 83.3, 120.3, 125.4, 127.28, 127.38, 127.41, 128.02, 128.05, 128.56, 128.59, 128.8, 129.7, 130.3, 134.7, 136.2, 141.6, 143.9, 144.2, 156.3, 170.5, 171.7; IR (neat) *v*_max_/cm^−1^ 1155, 1252, 1369, 1454, 1502, 1703, 3263; HRMS (ESI) *m*/*z* calculated for C_38_H_43_N_4_O_7_S [M + H]^+^ 699.2847; found 699.2861.

### 2.3. N-(Fmoc)Alaninyl-azasulfurylphenylalaninyl-D-phenylalanine *(**10**)*

*tert*-Butyl ester **9** (95 mg, 0.14 mmol) was treated with TFA:DCM 1:1 (1 mL) for 1.5 h at rt. The volatiles were evaporated to afford acid **10** as a solid (86 mg, 98%): mp 98 °C; [α]${}_{D}^{20}$ −31.7° (CHCl_3_, *c* 1.05); ^1^H NMR (CDCl_3_, 400 MHz) δ 0.70–0.90 (3H, m), 3.10–3.30 (2H, m) 3.90–4.15 (3H, m), 4.15–4.25 (1H, m), 4.25–4.35 (1H, m), 4.55–4.65 (1H, m), 4.65–4.75 (1H, m), 5.75–5.85 (1H, br), 5.85–6.00 (1H, br), 6.70–7.50 (17H, m), 7.60–7.75 (2H, m), 8.48 (1H, br); ^13^C NMR (CDCl_3_, 100 MHz) δ 17.2, 39.0, 47.1, 49.4, 55.5, 57.2, 67.8, 120.2, 125.4, 127.32, 127.39, 128.1, 128.6, 128.7, 129.8, 130.3, 134.2, 135.7, 141.5, 143.7, 144.1, 157.0, 172.9, 173.5; IR (neat) *v*_max_/cm^−1^ 1165, 1252, 1353, 1454, 1520, 1694, 3299; HRMS (ESI) *m*/*z* calculated for C_34_H_35_N_4_O_7_S [M + H]^+^ 643.2221; found 643.2231.

### 2.4. Fmoc-Ala-AsF-D-Phe-Lys(Boc)-NH-Rink resin *(**12**)*

A solution of acid **10** (77 mg, 0.12 mmol) and 2-(1H-benzotriazol-1-yl)-1,1,3,3- tetramethyluronium hexafluorophosphate (HBTU, 46 mg, 0.12 mmol) in dimethylformamide (DMF, 2 mL) was stirred for 1 min, treated with *N*,*N*-diisopropylethylamine (DIEA, 42 μL, 0.24 mmol), stirred for 5 min and added to resin **11** (188 mg, 0.64 mmol/g, 0.12 mmol) swollen in DMF (2 mL) in a 3-mL plastic filtration tube equipped with polyethylene filter. The resin mixture was shaken for 18 h at rt. The resin was filtered, washed and filtered sequentially using 15 s agitations with DMF (3×), MeOH (3×) and DCM (3×), and dried in vacuo to afford resin **12**. A resin aliquot (5 mg) was placed into a 1-mL plastic filtration tube equipped with polyethylene filter, treated with 20% piperidine in DMF and filtered (2 × 0.5 h), swollen and cleaved with 0.5 mL of a 95:2.5:2.5 cocktail of TFA:H_2_O:triethylsilane (TES) for 1 h at rt, and filtered. The filtrate was collected in a 1.5 mL Eppendorf^®^ tube, concentrated with air bubbles, treated with Et_2_O (1 mL), and centrifuged for 2 min. After decantation, the precipitate was dissolved in H_2_O (1 mL) and analyzed by LC-MS: R*t* 4.97 min on a Sunfire™ column using a gradient of 5–80% MeOH (0.1% FA) for 7.5 min + 90% MeOH (0.1% FA) for 2.0 min. The unreacted amine was capped after treatment of the resin with acetic anhydride (57 μL, 0.6 mmol) and DIEA (100 μL, 0.6 mmol) in DMF (2 mL), shaking for 0.5 h, washing as above, and drying in vacuo.

### 2.5. H-His-D-Trp-Ala-AsF-D-Phe-Lys-NH_2_ *(**3a**)*

The Fmoc group was removed from resin **12** on treatment (2 × 0.5 h) with a 20% piperidine in DMF solution followed by filtration, washing and drying as described above. The resin was swollen in DMF and treated with a premixed (1 min) solution of Fmoc-D-Trp(Boc)-OH (3.0 eq.) and HBTU (3.0 eq.) in DMF (2 mL) that was treated (5 min) with DIEA (6.0 eq.), and the mixture was shaken for 18 h at rt. The resin was filtered, washed, and dried as described for **12** above, then treated to remove the Fmoc group and coupled to Boc-His(Trt)-OH as described above. The resin (100 mg) was cleaved using a cocktail of TFA:H_2_O:TES (95:2.5:2.5, 2 mL) for 3 h in a cold room (4 °C), filtered and washed with TFA (2 × 2 mL). The filtrate was collected in a 50 mL Eppendorf™ tube, concentrated with air bubbles, treated with Et_2_O (50 mL) and centrifuged for 5 min. After decantation, the precipitate was dissolved in H_2_O (5 mL) and freeze-dried to a solid: analysis indicated peptide **3a** to be of 73% crude purity (R*t* 13.08 min) on a Gemini C18 column with a gradient of 0–80% MeOH [0.1% formic acid (FA)] in water (0.1% FA) for 30.0 min, followed by 90% MeOH (0.1% FA) in water (0.1% FA) for 10.0 min. Purification on a Gemini^®^ 5 micron C18 110A column (Phenomenex^®^ Inc. Torrance, CA, USA, 250 × 21.2 mm, 5 μm) with a gradient of 30–55% MeOH (0.1% FA) in water (0.1% FA), with a flow rate of 10.0 mL/min, to afford the desired formate salt **3a** (11.0 mg, 12%). The purified product was analyzed by analytical RP-HPLC using a Gemini^®^ 5 micron C18 110A column (Phenomenex^®^ Inc., 150 × 4.6 mm, 5 μm) and revealed to be of >99% purity: R*t* 18.95 min [5–80% MeOH (0.1% FA) in water (0.1% FA) for 30.0 min + 90% MeOH (0.1% FA) in water (0.1% FA) for 10.0 min]; R*t* 13.55 min [5–80% MeCN (0.1% FA) in water (0.1% FA) for 30.0 min + 90% MeCN (0.1% FA) in water (0.1% FA) for 10.0 min]. HRMS (ESI) *m*/*z* calculated for C_42_H_54_N_12_NaO_7_S [M + Na]^+^ 893.3851; found 893.3832.

### 2.6. N-(Alloc)Alaninyl-azasulfurylglycinyl(Fmoc)-D-phenylalanine tert-butyl ester *(**20**)*

A solution of Na_2_CO_3_ (132 mg, 1.24 mmol) and AsG-peptide **19** (390 mg, 0.8863 mmol) in 1:1 water:1,4-dioxane (14 mL) at 0 °C was treated with a solution of Fmoc-OSu (364 mg, 1.08 mmol) in dioxane (7 mL), stirred for 1 h, and allowed to warm to rt with stirring for 18 h. Brine (25 mL) was added to the mixture. The aqueous phase was extracted with EtOAc (3 × 50 mL). The organic phases were combined, dried over MgSO_4_, filtered and evaporated to a residue, which was purified on two consecutive flash chromatography [29] columns eluting first with 3:1 hexane/EtOAc and second with 19:1 DCM/EtOAc. Evaporation of the collected fractions afforded fluorenyl carbazate **20** (396 mg, 69%) as a solid: R*_f_* 0.27 (hexane:EtOAc 3:1), mp 83 °C; [α]${}_{D}^{20}$ −14.6° (MeOH, *c* 0.98); ^1^H NMR (CD_3_OD, 300 MHz) showed an 7:50 mixture of carbazate isomers (^1^H and ^13^C signals of the minor isomer are respectively presented in brackets and parentheses): δ 1.32 (9H, s), [1.36, (9H, s)], 1.34 (3H, d, *J* = 7.1), [1.43 (3H, d, *J* = 7.1)], 2.90–3.10 (2H, m), 4.20-4.40 (3H, m), 4.40–4.60 (4H, m), 5.13 (1H, d, *J* = 10.7), [5.19 (1H, d, *J* = 10.7)] 5.26 (1H, d, *J* = 16.4), [5.32 (1H, d, *J* = 16.4)], 5.80–6.00 (1H, m), 7.15–7.35 (7H, m), 7.40 (2H, t, *J* = 7.4), 7.60–7.70 (2H, m), 7.81 (2H, d, *J* = 7.4); ^13^C NMR (CD_3_OD, 75 MHz):^6^ δ 19.1, 28.97 (29.01), 40.6 (40.8), 48.6, 51.2, 60.2 (60.4), 67.5, 71.3 (71.8), 83.9 (84.3), 118.6, 121.8 (121.9), 127.10 (127.17), 127.3 (127.4), 128.7, 129.2, 129.8, 130.2, 131.6 (131.7), 135.04 (135.10), 138.4 (138.5), 143.31 (143.37), 145.5 (145.6), 145.6 (145.7), 154.2, 172.4 (172.7), 175.0; IR (neat) *v*_max_/cm^−1^ 1152, 1178, 1226, 1316, 1369, 1384, 1450, 1498, 1695, 2978, 3292; HRMS (ESI) *m*/*z* calculated for C_35_H_40_N_4_NaO_9_S [M + Na]^+^ 715.2408; found 715.2424.

### 2.7. N-(Alloc)Alaninyl-azasulfurylglycinyl(Fmoc)-D-phenylalanine *(**21**)*

Acid **21** was synthesized from ester **20** (0.750 g, 1.1 mmol) using the protocol described for **10**. The volatiles were evaporated to afford **21** as solid (0.682 g, 99%): [α]${}_{D}^{20}$ −36.8° (MeOH, *c* 0.98); ^1^H NMR (CD_3_OD, 300 MHz) showed a 9:20 mixture of carbazate isomers (^1^H and ^13^C signals of the minor isomer are respectively presented in brackets and parentheses): δ 1.34 (3H, d, *J* = 7.1) [1.42 (3H, d, *J* = 7.1)], 3.00–3.20 (2H, m), 4.20–4.40 (3H, m), 4.40–4.55 (3H, m), 4.55–4.70 (1H, m), 5.13 (1H, d, *J* = 10.8) [5.18 (1H, d, *J* = 10.8)], 5.32 (1H, d, *J* = 15.7) [5.37 (1H, d, *J* = 15.7)], 5.80–6.00 (1H, m), 7.15–7.35 (7H, m), 7.40 (2H, t, *J* = 7.4), 7.60–7.70 (2H, m), 7.80 (2H, d, *J* = 7.4); ^13^C NMR (CD_3_OD, 75 MHz):^6^ δ 19.1 (19.2), 40.4, 48.54 (48.63), 51.17 (51.41), 59.9, 67.5, 71.4 (71.8), 118.6, 121.8 (121.9), 127.2 (127.4), 128.7, 129.2, 129.8, 130.2, 131.6, 135.1, 138.4 (138.5), 143.3 (143.4), 145.6 (145.7), 154.1, 158.7, 174.8, 175.0. IR (neat) *v*_max_/cm^−1^ 1178, 1220, 1315, 1384, 1449, 1519, 1543, 1643, 1688, 1737, 3071, 3289; HRMS (ESI) *m*/*z* calculated for C_31_H_33_N_4_O_9_S [M + H]^+^ 637.1963; found 637.1970.

### 2.8. Alloc-Ala-AsG(Fmoc)-D-Phe-Lys(Boc)-NH-Rink Resin *(**22**)*

A solution of AsG(Fmoc)-tripeptide **21** (396 mg, 0.621 mmol) and *N*,*N*’-diisopropylcarbodiimide (DIC, 97 μL, 0.621 mmol) in DMF (2 mL) was stirred for 1 min, treated with hydroxybenzotriazole (HOBt, 84 mg, 0.621 mmol), stirred for 5 min, and added to resin **11** (497 mg, 1.00 mmol/g, 0.497 m mol) swollen in DMF (2 mL) in a 3-mL plastic filtration tube equipped with a polyethylene filter. The resin mixture was shaken for 18 h at rt, filtered, washed and dried as described above to afford resin **22**, an aliquot of which was analyzed as described above: R*t* 5.11 min on a Sunfire™ column using a gradient of 5–80% MeOH (0.1% FA) in water (0.1% FA) for 7.5 min followed by 90% MeOH (0.1% FA) in water (0.1% FA) for 2.0 min. The resin was capped with acetic anhydride as described above and dried in vacuo.

### 2.9. Alloc-Ala-AsY(TBDMS)-D-Phe-Lys(Boc)-NH-Rink resin *(**24b**)*

The Fmoc group was removed from resin **22** (100 mg, 0.071 mmol), which was washed and dried as described in the synthesis of **3a** above. A solution of 40% tetraethylammonium hydroxide in water (79 μL, 0.214 mmol) followed by 4-*tert*-butyldimethylsilyl(TBDMS)oxybenzyl bromide (65 mg, 0.214 mmol) were added to resin **23** swollen in DMF (2 mL), and the mixture was shaken at rt for 18 h, filtered, washed as described above and dried in vacuo. Analysis of a cleaved resin aliquot of **24b** as described above by LC-MS indicated AsY(TBDMS)-peptide to be of 53% purity (R*t* 6.09 min) contaminated with bis-alkylation product of 6% purity (R*t* 6.09 min) using a Sunfire™ column with a gradient of 5–80% MeOH (0.1% FA) in water (0.1% FA) for 7.5 min followed by 90% MeOH (0.1% FA) in water (0.1% FA) for 2.0 min.

Alloc-Ala-AsF-D-Phe-Lys(Boc)-NH-Rink resin (**24a**) was prepared and analyzed as **24b** using benzyl bromide (26 μL, 0.214 mmol) and indicated to be of 77% purity (R*t* 5.89 min) using an initial gradient of 20–80% MeOH (0.1% FA) in water (0.1% FA) for 6 min.

Alloc-Ala-(4-F)AsF-D-Phe-Lys(Boc)-NH-Rink resin (**24c**) was prepared and analyzed as **24b** using 4-fluorobenzyl bromide (27 μL, 0.214 mmol) and indicated to be of 72% purity (R*t* 6.86 min) contaminated with bis-alkylation product of 10% purity (R*t* 8.33 min) using an initial gradient of 5–80% MeOH (0.1% FA) for 7.5 min.

Alloc-Ala-(4-MeO)AsF-D-Phe-Lys(Boc)-NH-Rink resin (**24d**) was prepared and analyzed as **24b** using 4-methoxybenzyl bromide (32 μL, 0.214 mmol) and indicated to be of 64% purity (R*t* 6.71 min) contaminated with bis-alkylation product of 12% purity (R*t* 8.07 min) using an initial gradient of 5–80% MeOH (0.1% FA) in water (0.1% FA) for 7.5 min.

Alloc-Ala-AsNal-D-Phe-Lys(Boc)-NH-Rink resin (**24e**) was prepared and analyzed as **24b** using 2-bromomethylnaphthalene (47 mg, 0.214 mmol) and indicated to be of 77% purity (R*t* 7.71 min) contaminated with bis-alkylation product of 9% purity (R*t* 7.71 min) using an initial gradient of 5–80% MeOH (0.1% FA) in water (0.1% FA) for 7.5 min.

### 2.10. H-His-D-Trp-Ala-(4-F)AsF-D-Phe-Lys-NH_2_ *(**3c**)*

Resin **24c** was swollen in DMF (2 mL) for 30 min, filtered, and treated with a solution of Pd(PPh_3_)_4_ (16 mg, 0.014 mmol, freshly washed with EtOH) and *N*,*N*-dimethylbarbituric acid (111 mg, 0.47 mmol, 7.0 eq.) in 2 mL of 3:2 DCM:DMF. After shaking at rt for 2 h, the resin was filtered and retreated with the same conditions for a second time for 2 h. The resin was filtered, washed as above, treated with 0.5% sodium diethylthiocarbamate (2 mL) for 30 min, filtered and washed two more times with 0.5% sodium diethylthiocarbamate (2 mL) for 15 s. After washing as described above, analysis of a cleaved aliquot of resin showed a R*t* 4.28 min: Sunfire™ column; gradient: 5–80% MeOH (0.1% FA) for 7.5 min + 90% MeOH (0.1% FA) for 2.0 min. The (4-F)Asf-peptide resin was elongated, cleaved and purified as described above for the synthesis of AsF-peptide **3a**: 65% crude purity (R*t* 5.96 min) on a Sunfire™ column using a gradient of 5–80% MeOH (0.1% FA) in water (0.1% FA) for 7.5 min followed by 90% MeOH (0.1% FA) in water (0.1% FA) for 2.0 min. Purification by preparative RP-HPLC using a Gemini^®^ 5 micron C18 110A column (Phenomenex^®^ Inc., 250 × 21.2 mm, 5 μm) with a gradient of 20–50% MeOH (0.1% FA) in water (0.1% FA), with a flow rate of 10.0 mL/min afforded (4-F)Asf-peptide **3c** (1.0 mg, 3%) as FA salt: >99% purity (R*t* 5.23 min) 5–80% MeOH (0.1% FA) in water (0.1% FA) for 7.5 min followed by 90% MeOH (0.1% FA) in water (0.1% FA) for 2.0 min]; (R*t* 3.59 min) 5–80% MeCN (0.1% FA) in water (0.1% FA) for 7.5 min followed by 90% MeCN (0.1% FA) for 2.0 min. HRMS (ESI) *m*/*z* calculated for C_42_H_53_FN_12_NaO_7_S [M + Na]^+^ 911.3757; found 911.3769.

H-His-D-Trp-Ala-AsF-D-Phe-Lys-NH_2_ (**3a**) was synthesized in 82% crude purity from resin **24a** using the same protocols described above for **3c** and exhibited identical retention time as material prepared from AsF-peptide **10**.

H-His-D-Trp-Ala-AsF(4-HO)-D-Phe-Lys-NH_2_ (**3b**) was synthesized from resin **24b** using the same protocols described above for **3c** [70% crude purity (R*t* 5.03 min) 10–40% MeOH (0.1% FA) in water (0.1% FA) for 7.5 min, purified by preparative RP-HPLC using a gradient of 20–50% MeOH (0.1% FA) in water (0.1% FA) to afford **3b** as the FA salt: 3.4 mg, 9% yield, >99% purity (R*t* 5.03 min) 15–45% MeOH (0.1% FA) in water (0.1% FA) for 7.5 min; (R*t* 5.22 min) 15–40% MeCN (0.1% FA) in water (0.1% FA) for 7.5 min]. HRMS (ESI) *m*/*z* calculated for C_42_H_54_N_12_NaO_8_S [M + Na]^+^ 909.3801; found 909.3805.

H-His-D-Trp-Ala-(4-MeO)AsF-D-Phe-Lys-NH_2_ (**3d**) was synthesized from resin **24d** using the same protocols described above for **3c** [75% crude purity (R*t* 5.26 min), purified by preparative RP-HPLC using a gradient of 15–45% MeOH (0.1% FA) in water (0.1% FA) to afford **3d** as the FA salt: 4.1 mg, 10% yield, >99% purity (R*t* 5.40 min) 15–45% MeOH (0.1% FA) in water (0.1% FA); (R*t* 6.19 min) 10–30% MeCN (0.1% FA) in water (0.1% FA) for 7.5 min]. HRMS (ESI) m/z calculated for C_43_H_56_N_12_NaO_8_S [M + Na]^+^ 923.3957; found 923.3936.

H-His-D-Trp-Ala-AsNal-D-Phe-Lys-NH_2_ (**3e**) was synthesized from resin **24e** using the same protocols described above for **3c** [61% crude purity (R*t* 5.92 min) 5–80% MeOH (0.1% FA) in water (0.1% FA) for 7.5 min, purified by preparative RP-HPLC using a gradient of 20–50% MeOH (0.1% FA) in water (0.1% FA) to afford **3e** as the FA salt: 4.2 mg, 10% yield, >99% purity (R*t* 3.89 min) 25–55% MeOH (0.1% FA) in water (0.1% FA) for 7.5 min; (R*t* 4.83 min) 15–35% MeCN (0.1% FA) in water (0.1% FA) for 7.5 min]. HRMS (ESI) *m*/*z* calculated for C_46_H_56_N_12_NaO_7_S [M + Na]^+^ 943.4008; found 943.4007.

H-Ala-D-Trp-Ala-AsF(4-F)-D-Phe-Lys-NH_2_ (**3f**) was synthesized from resin **24c** using the same protocols described above for **3c** except replacing Fmoc-Ala-OH for Fmoc-His(Tr)-OH [79% purity (R*t* 5.96 min) 20–80% MeOH (0.1% FA) in water (0.1% FA) for 14 min, purified by preparative RP-HPLC using 35–50% MeOH (0.1% FA) in water (0.1% FA) to afford the **3f** as the FA salt: 6.5 mg, 23% yield, >99% purity (R*t* 6.79 min) 20–80% MeOH (0.1% FA) in water (0.1% FA) for 7.5 min; (R*t* 7.65 min) 10–80% MeCN (0.1% FA) in water (0.1% FA) for 7.5 min]. HRMS (ESI) m/z calculated for C_39_H_51_FN_10_O_7_S [M + Na]^+^ 845.3647; found 845.3539.

### 2.11. N-(Fmoc)Alanine-N’-4-fluorobenzylhydrazide *(**29**)*

A solution of Fmoc-alanine (1.2 eq., 3.11 g, 9.99 mmol) in THF (50 mL) at –15°C was treated slowly with isobutyl chloroformate (1.2 eq., 1.36 g, 1.3 mL, 9.99 mmol) and *N*-methyl morpholine (1.5 eq., 1.26 g, 1.37 mL, 12.5 mmol), stirred for 15 min, treated slowly with a solution of *tert*-butyl *N*-(4-fluorobenzyl)carbazate (**28**, 1 eq., 2 g, 8.32 mmol) in THF (50 mL), stirred for 3 h, diluted with EtOAc and washed with water and brine. The organic phases were combined, dried over Na_2_SO_4_, filtered and evaporated to a residue that was purified by flash chromatography on silica gel eluting with 20–40% EtOAc in hexane to afford *N*-(Fmoc)alanine-*N*’-4-fluorobenzyl *N*’-(Boc)hydrazide (3.82 g, 7.16 mmol, 86%) as white solid: mp 68−69 °C; R*_f_* 0.25 (EtOAc:hexane 3:2); ^1^H NMR (500 MHz, CDCl_3_) δ 7.76 (d, *J* = 7.6 Hz, 2H), 7.55 (d, *J* = 7.3 Hz, 2H), 7.40 (t, *J* = 7.4 Hz, 2H), 7.34–7.27 (m, 2H), 7.24–7.16 (m, 2H), 6.97–6.89 (m, 2H), 5.39–5.11 (m, 1H), 4.70–4.49 (m, 2H), 4.42–4.24 (m, 2H), 4.24–4.04 (m, 2H), 1.45 (s, 9H), 1.36 (d, *J* = 4.4 Hz, 3H); ^13^C NMR (CDCl_3_, 125 MHz) δ 163.5, 161.5, 156.1, 143.8, 141.5, 132.4, 130.5, 127.9, 127.2, 125.1, 120.2, 115.7, 115.5, 67.4, 60.5, 47.2, 28.3, 21.2, 18.1, 14.3; IR (neat) *v*_max_/cm^−1^ 1154, 1220, 1248, 1367, 1391, 1450, 1509, 1536, 1678, 2978, 3276; HRMS *m*/*z* calculated for C_30_H_32_FN_3_O_5_ [M + Na]^+^ 556.2218; found 556.2234. The hydrazide (1 eq., 3.5 g, 6.56 mmol) was dissolved in DCM (35 mL), treated with trifluoroacetic acid (20 mL) and stirred for 3 h. The volatiles were evaporated. The residue was dissolved in DCM and evaporated (3×), dissolved in EtOAc (150 mL), washed with sat. K_2_CO_3_ (150 mL) and H_2_O (100 mL), dried over MgSO_4_, filtered and evaporated to afford hydrazide **29** (2.22 g, 5.12 mmol, 78%) as white powder: mp 176 °C; ^1^H NMR (400 MHz, DMSO) δ 9.32 (s, 1H), 7.89 (d, *J* = 6.8 Hz, 2H), 7.78–7.55 (m, 2H), 7.55–7.40 (m, 3H), 7.40–7.20 (m, 4H), 7.20–6.96 (m, 2H), 5.23 (br s, 1H), 4.36–4.11 (m, 3H), 4.11–3.92 (m, 1H), 3.82 (s, 2H), 1.14 (d, *J* = 6.2 Hz, 3H); ^13^C NMR (DMSO, 100 MHz) δ 171.6, 155.6, 1439, 143.8, 140.7, 134.8, 130.53, 130.45, 127.6, 127.1, 125.3, 120.1, 114.8, 114.6, 65.6, 53.6, 48.7, 46.6, 18.3; IR (neat) *v*_max_/cm^−1^ 1105, 1266, 1330, 1453, 1509, 1542, 1651, 1696 3275, 3304; HRMS *m*/*z* calculated for C_25_H_24_FN_3_O_3_ [M + Na]^+^ 456.1694; found 456.1697.

### 2.12. N-(Fmoc)Alaninyl-4-fluoro-azasulfurylphenylalaninyl-D-phenylalanine tert-butyl ester *(**31**)*

Hydrazide **29** (1.3 eq., 1.4 g, 3.23 mmol) was added to a microwave vessel containing a solution of 4-nitrophenyl D-phenylalanine *tert*-butyl ester sulfamidate (**30**, 1 eq., 1.05 g, 2.48 mmol, prepared according to ref 20) in DCE (10 mL), treated with DIEA (1.3 eq., 0.534 mL, 3.23 mmol), at which point the solution turned yellow. The vessel was sealed and heated to 60 °C using microwave irradiation for 2.5 h. The volatiles were evaporated. The residue was purified by flash chromatography eluting with a solution of 7:3 Et_2_O:pet. ether. The collected fractions were evaporated to a residue, which was dissolved in DCM (25 mL), washed with sat. NaHCO_3_, dried over Na_2_SO_4_, filtered and evaporated to afford azasulfuryltripeptide **31** (0.944 g, 1.32 mmol, 53%) as white solid: mp 74–76 °C; R*_f_* 0.35 (7:3 Et_2_O:petroleum ether); ^1^H NMR (500 MHz, CDCl_3_) δ 7.75 (d, *J* = 7.6 Hz, 2H), 7.67 (s, 1H), 7.57–7.50 (m, 2H), 7.42–7.35 (m, 2H), 7.35–7.27 (m, 3H), 7.25–7.16 (m, 6H), 6.97 (t, *J* = 8.6 Hz, 2H), 5.49 (d, *J* = 6.6 Hz, 1H), 5.13 (d, *J* = 6.9 Hz, 1H), 4.51 (s, 2H), 4.47–4.40 (m, 1H), 4.39–4.28 (m, 2H), 4.19–4.12 (m, 1H), 4.08–3.99 (m, 1H), 3.21–3.09 (m, 2H), 1.40 (s, 9H), 1.20 (d, *J* = 6.5 Hz, 3H); ^13^C NMR (CDCl_3_, 125 MHz) δ 171.3, 170.3, 143.7, 141.5, 135.9, 131.3, 131.2, 130.3, 130.1, 128.5, 127.9, 127.3, 127.2, 125.2, 120.1, 115.7, 115.6, 83.3, 67.5, 57.3, 54.5, 49.3, 47.1, 38.8, 28.1, 17.5; IR (neat) *v*_max_/cm^−1^ 1151, 1222, 1365, 1450, 1479, 1601, 1695, 2978, 3280; HRMS *m*/*z* calculated for C_38_H_41_FN_4_O_7_S [M + Na]^+^ 739.2572; found 739.2587. 

### 2.13. N-(Fmoc)Alaninyl-4-fluoro-azasulfurylphenylalaninyl-D-phenylalanine *(**32**)*

*tert*-Butyl ester **31** (1 eq., 0.9 g, 1.26 mmol) was treated with TFA:DCM 1:1 (9 mL) for 1.5 h at room temperature. The volatiles were then evaporated, residue obtained was dissolved in DCM and evaporated (3×) to afford acid **32** (0.75 g, 1.14 mmol, 91%) as white solid: mp 74–75 °C; ^1^H NMR (400 MHz, CDCl_3_) δ 8.43 (s, 1H), 7.79 (d, *J* = 7.5 Hz, 2H), 7.67–7.51 (m, 1H), 7.51–7.46 (m, 1H), 7.48–7.39 (m, 2H), 7.39–7.31 (m, 3H), 7.09–6.95 (m, 2H), 6.39 (br s, 3H), 6.06–5.72 (m, 2H), 4.79–4.54 (m, 2H), 4.43–4.27 (m, 2H), 4.27–4.09 (m, 2H), 4.09–3.96 (m, 1H), 3.42–3.15 (m, 2H), 1.00 (s, 3H); ^13^C NMR (CDCl_3_, 125 MHz) δ 173.4, 164.1, 161.6, 157.0, 141.3, 135.3, 131.5, 131.4, 130.0, 128.5, 128.01, 127.99, 127.3, 125.1, 120.2, 115.6, 115.4, 67.8, 57.0, 54.7, 49.3, 46.9, 38.8, 16.9; IR (neat) *v*_max_/cm^−1^ 1103, 1159, 1218, 1347, 1391, 1449, 1477, 1509, 1697, 3276; HRMS *m*/*z* calculated for C_34_H_33_FN_4_O_7_S [M + Na]^+^ 683.1946; found 683.1956. 

### 2.14. N-(Fmoc)-Ala-(4-F)AsF-D-Phe-Lys(Boc)-NH-Rink resin *(**33**)*

Acid **32** (1.5 eq., 0.445 g, 0.673 mmol) was coupled to resin **11** (1 eq., 0.9 g, 0.449 mmol) according to the protocol described for the synthesis of resin **22**. Analysis of a cleaved aliquot showed (Rt 4.97 min) 10–90% MeOH (0.1% FA) for 10 min. Following the protocols above for **3a**, the resin was capped with acetic anhydride, elongated, cleaved and purified to give (4-F)AsF-peptide **3c** having identical chromatographic and spectrometric properties as described above. 

### 2.15. In Vitro Assessment of Anti-inflammatory Activity of Azasulfurylpeptide in Macrophages

#### 2.15.1. Modulation of TLR2 Agonist-induced Overproduction of Nitric Oxide (NO) in J774 Macrophage Cell Line by Azasulfurylpeptides 

The murine J774A.1 macrophage cell lines were obtained from American Type Cell Collection (ATCC #TIB-67), seeded at 1.5 × 10^5^ cells/well in Dulbecco’s modified eagle media (DMEM) containing 100U/mL of penicillin (Pen) and 100 μg/mL of streptomycin (Strep) on a 48-well plate (Costar^®^ #3548), and incubated at 37 °C with 5% CO_2_. After 2 h, the medium of adhered cells was changed to DMEM-Pen/Strep containing 0.2% of bovine serum albumin (BSA), and supplemented with either azasulfurylpeptide **3a**–**f** or [azaK^6^]-GHRP-6 as a negative control. After 2 h of pre-incubation, the cells were stimulated overnight with 300 ng/mL of R-FSL-1. Supernatants were collected for fluorescence determination of nitrite using 2,3-diaminonaphtalene (DAN). Briefly, 25 μL of sample was incubated with 0.5 μg of DAN in a 100 μL final volume of phosphate buffer (50 mM, pH 7.5) at rt in the dark. After 15 min, the reaction was stopped with 20 µL of NaOH (2.8N) and the plate was read using a fluorescence plate reader (TECAN^®^ Safire, λ_exc_ 365 nm and λ_em_ 430 nm).

#### 2.15.2. Modulation of NF-κB Activation and Proinflammatory Cytokine and Chemokine Production in Macrophages by Azasulfurylpeptides

Peritoneal macrophage cells from C57BL/6 mice were harvested from sterile DMEM cell-culture medium (Wisent # 319-005-CL), allowed to adhere for 1 h at 37 °C in a 5% CO_2_ atmosphere, and washed twice with PBS to remove non-adherent cells. Macrophages (0.5 × 10^6^ cells/well) were plated in 48 well-culture plates with DMEM containing 0.2% of bovine serum albumin (BSA), pretreated for 2 h with azasulfurylpeptides **3b**–**e** or [azaK^6^]-GHRP-6 (1 µM), before stimulation with a TLR2 ligand: R-FSL-1 (Invivogen^®^ #L7022) at 300 ng/mL interferon gamma (IFNγ R&D Systems^®^ Minneapolis, MN, USA # 172-5201) at 20 ng/mL for 4 or 24 h. The supernatants were recovered, and ELISA (eBioscience^®^ #88-7324; 88-7391) was performed to determine the levels of proinflammatory cytokine and chemokine (TNFα, MCP-1). The effect on NF-κB activation was documented on cell lysates after 0, 5, 10, 15 and 30 min stimulation with R-FSL1, using NF-κB p65/RelA specific ELISA-based assay (eBioscience^®^ #85-86083). The results were expressed as mean ± SEM and analyzed statistically by a one-way analysis of variance (ANOVA) with a Dunnett as a post-test. Level of significance was set at *P* < 0.05.

### 2.16. In Vivo Validation of Anti-inflammatory Activity of Azasulfurylpeptide in ApoE-deficient Murine Model 

#### 2.16.1. Animals

Apo E deficient (*ApoE^−/−^*) mice were obtained from The Jackson Laboratory (Bar Harbor, ME, USA). Animals were housed under 12 h light/dark cycles and fed ad libitum with a standard rodent diet. All experimental procedures were done in accordance with the Animal Ethics Committee at Université de Montreal (CDEA, project 12-012 approved on 26 January 2013) and the Canadian Council on Animal Care guidelines for use of experimental animals.

#### 2.16.2. Blue-light Illumination Model

Three to four-month-old ApoE^−/−^ mice were exposed to blue LED-light (Yescom USA, Inc., City Of Industry, CA, USA) for 5 d at 1000 lux illumination without previous dark-adaptation. For pupil dilatation, ophthalmic atropine solution 1% (Alcon, Fort Worth, TX, USA) was applied to both eyes daily. Aza- and azasulfurylpeptides **1**, **2** and **3c** or [azaK^6^]-GHRP-6 (289 nmol/kg) were administered subcutaneously at 24 h following blue-light exposure for 7 consecutive days. At the end of the blue-light exposure period, the mice were maintained on a 12:12 h light: dark cycle for 3 days, before being sacrificed. Enucleated eyes were fixed in 4% paraformaldehyde (PFA) for 15 min at rt and sectioned at the limbus; the anterior segments were discarded. The posterior eye cups consisting of retinal pigment epithelium (RPE)/choroid/sclera complex were collected and prepared for immunofluorescence analysis. 

#### 2.16.3. Retinal Tissue Preparation and Immunofluorescence Staining

RPE/choroid/sclera flat-mounts were treated with PBS solution containing 0.1% Triton 100× and 10% fetal bovine serum (FBS) or 5% BSA (saturation buffer) for 45 min. Specimens were incubated overnight at 4 °C with primary antibodies diluted in saturation buffer. Immunofluorescence was performed using polyclonal rabbit anti-IBA-1 as marker of activated cells. The corresponding secondary antibody, AlexaFluor 488-conjugated antibody was used to reveal the primary antibody. Flatmounts were analyzed using an IX81 Olympus fluorescence microscope (Richmond Hill, ON, Canada). All immunostainings were repeated at least three times and staining that omitted the primary antibody served as negative control. 

### 2.17. Quantification of Activated Microglia/Macrophages in the Subretinal Space 

IBA-1–stained cells were counted on flatmounts from control and illuminated mice injected daily with 0.9% NaCl or with aza- and azasulfurylpeptides **1**, **2** and **3c** or [azaK^6^]-GHRP-6 (289 nmol/kg). Cell numbers were expressed as the mean number of IBA-1–positive cells in subretinal space. Statistical analysis was performed by using a one-way ANOVA with a Dunnett as a post-test. Level of significance was set at *P* < 0.05.

### 2.18. Modulatory Effect of Azasulfuryl on Microvascular Sprouting as Index of Angiogenesis on Choroid Explant 

Choroidal explants were set as described previously [15]. Briefly, enucleated mice eyes with and without treatment with aza- and azasulfurylpeptides **1**, **2** and **3c** or [azaK^6^]-GHRP-6 were collected and dissected to obtain the complex consisting of the RPE/choroid/sclera, which was cut in 16 explant fragments and cultured in 20 µL of Matrigel (BD biosciences, San Jose, CA, USA) in 24-wells plates (Sarstedt Inc., St. Leonard, QC, Canada). Explant growth was performed in endothelial cell growth medium (EGM) over 3 days, until the endothelial cells were found to proliferate around the explant. Phototographs of explants were taken under microscopy (T0 and T24 h). The explants were washed with endothelial basal medium (EBM) and incubated in EBM with NaCl or CD36 ligands at 10^−6^ M for 24 h. The sprouting surface was assessed by image analysis using imagen J software. Ratios (sprouting surface at T24 h, sprouting surface at T0)/sprouting surface at T0 were determined. Numerical results were expressed as mean ± SEM. Data were compared using one-way ANOVA with Dunnett’s post-test. *P* < 0.05 was considered statistically significant (Prism software version 5.01; graphpad Software, San Diego, CA, USA).

## 3. Results and Discussion 

**Scheme 1:**
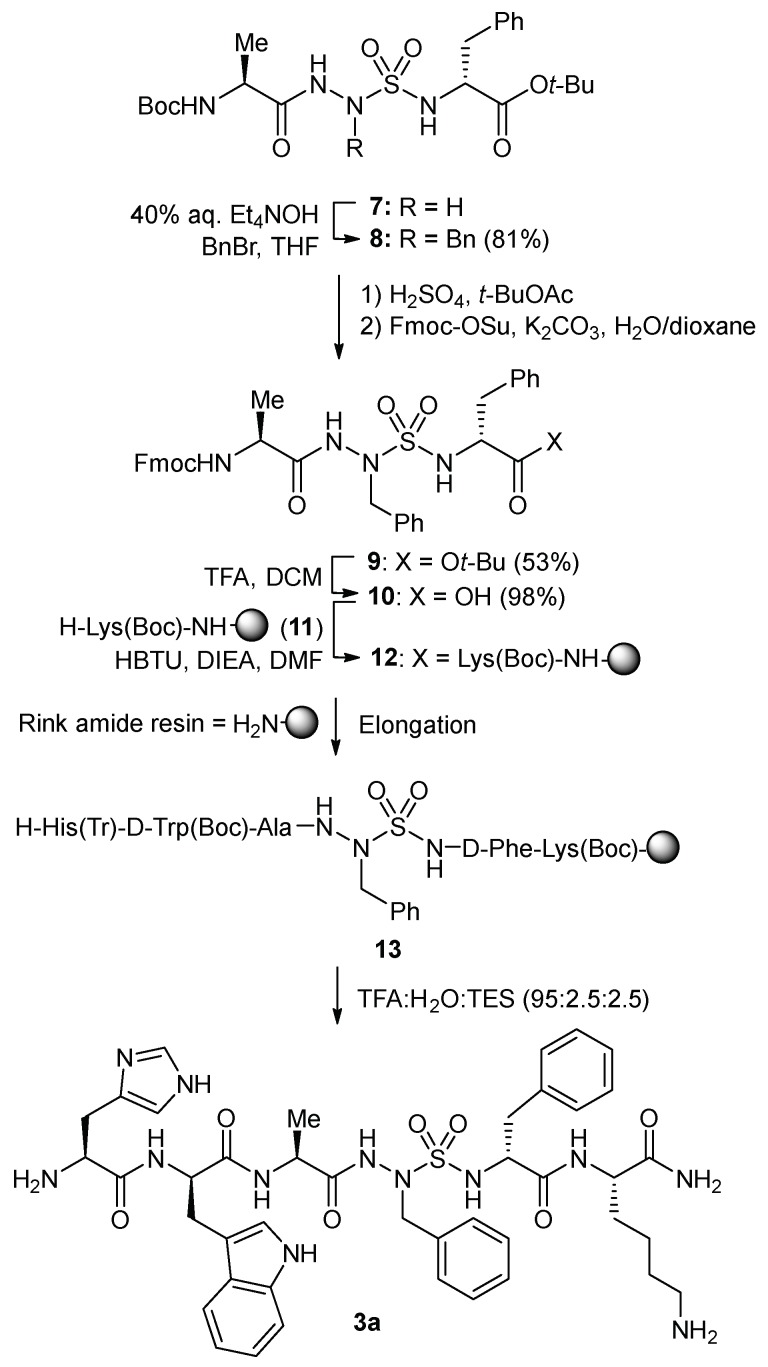
Synthesis of [AsF^4^]-GHRP-6 (**3a**).

**Scheme 2:**
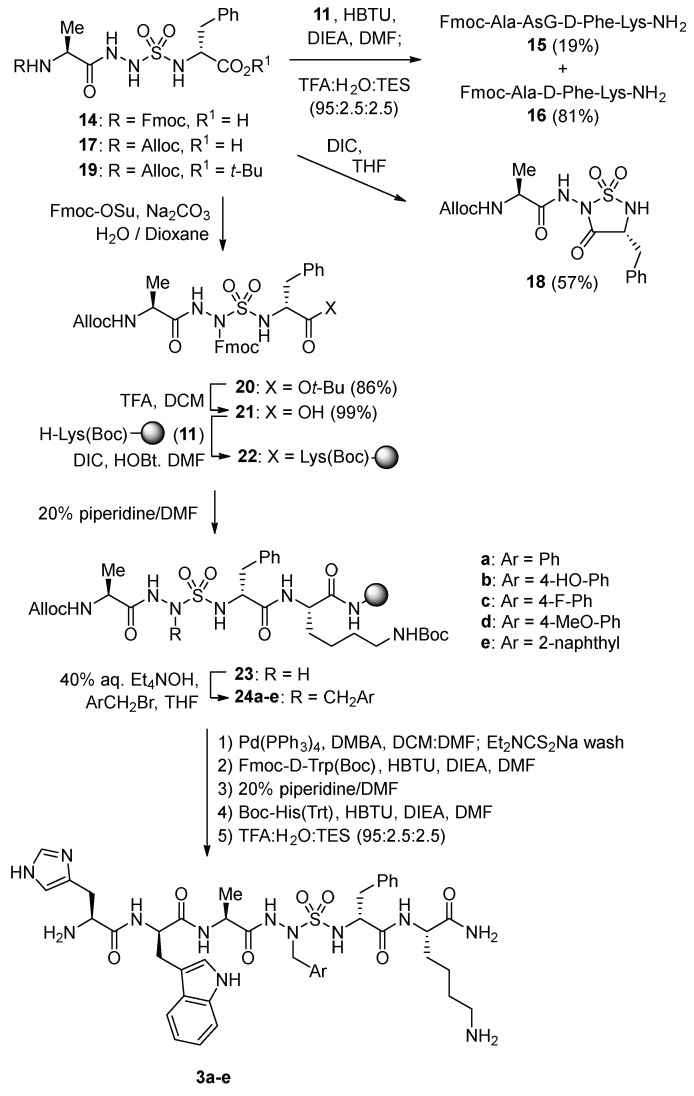
Diversity-oriented solid-phase synthesis of azasulfurylpeptides **3a**–**e.**

**Scheme 3:**
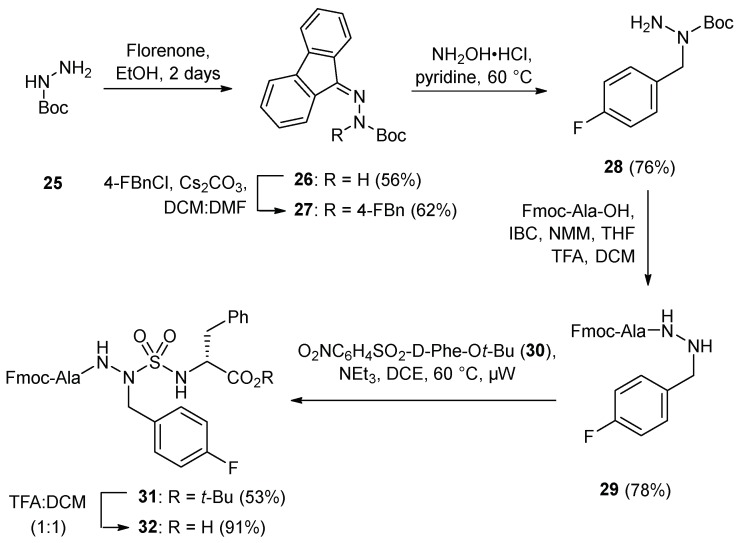
Synthesis of (4-F)AsF tripeptide 32.

*N*-Aminosulfamide residues were introduced at the 4-position of GHRP-6 analogs by solid-phase synthesis strategies featuring incorporation of azasulfuryl tripeptide building blocks that were prepared in solution. Initially, azasulfurylphenylalanine (AsF) tripeptide **10** was linked to the solid support and elongated to prepare azasulfurylpeptide **3a** (Scheme 1). Subsequently, a diversity-oriented strategy was developed in which an azasulfurylglycine (AsG) residue was introduced into the resin-bound peptide and alkylated on solid-support to prepare the set of azasulfurylpeptides **3a**–**e** (Scheme 2). Finally, a procedure was developed for scaling up the synthesis of azasulfurylpeptide **3c** (Scheme 3).

To install an AsF residue at the 4-position of GHRP-6, the protected azasulfuryl tripeptide Fmoc-Ala-AsF-D-Phe-OH (**10**) was first synthesized in solution by a route featuring alkylation of Boc-Ala-AsG-D-Phe-O*t*-Bu (**7**, Scheme 1) [20]. Treatment of AsG peptide **7** with benzyl bromide and tetraethylammonium hydroxide in THF provided AsF peptide **8** in 81% yield [20]. The Boc protection was selectively cleaved from peptide **8** without loss of the *tert*-butyl ester using sulfuric acid in *tert*-butylacetate [30], and amine protection with Fmoc-OSu and K_2_CO_3_ gave Fmoc-Ala-AsF-D-Phe-O*t*-Bu (**9**) in 53% yield. Cleavage of the *tert*-butyl ester with 1:1 TFA/CH_2_Cl_2_ gave acid **10**, which was coupled to *N*^ε^-(Boc)lysinyl Rink amide resin **11** using HBTU and DIEA in DMF. After capping the resin with acetic anhydride, conventional Fmoc-based solid-phase synthesis was used to elongated peptide **12** by amino acid couplings and Fmoc group removals, employing Fmoc-D-Trp(Boc)-OH and Boc-His(Trt)-OH [31]. Cleavage of peptide **13** from the resin using a solution of TFA:H_2_O:TES (95:2.5:2.5) in a cold room (4 °C), and purification by HPLC afforded [AsF^4^]-GHRP-6 (**3a**) in 12% overall yield from tripeptide **10**.

Alkylation of a resin-bound AsG peptide was pursued to facilitate introduction of side-chain diversity for structure–activity relationship studies (Scheme 2). Initial attempts failed however to couple AsG tripeptide **14** onto Lys(Boc) amide resin **11** and LC-MS analysis of a resin cleaved aliquot indicated little conversion to the desired AsG peptide **15** (19%); instead, the major cleaved product was a deletion sequence, Fmoc-Ala-Lys-NH_2_ (**16**, 81%).

Treatment of AsG tripeptide **17** with DIC in THF afforded sulfahydantoin **18** in 57% yield [32]. Considering azasulfuryltripeptide **10** coupled effectively, the absence of a sulfamide substituent was hypothesized to account for the degradation of AsG tripeptides **14** and **17** leading, respectively, to the deletion sequence **16** and sulfahydantoin **18**. To avoid removal of the sulfamide proton leading to side-reactions of the AsG tripeptide, a protection strategy was pursued. Azasulfuryltripeptides bearing allyl and phenylsulfonylethyl side chains on the *N*-aminosulfamide coupled effectively, but attempts to liberate the AsG residue failed. On the other hand, acylation of *N*-Alloc-Ala-AsG-D-Phe-O*t*Bu (**19**) with Fmoc-OSu gave *N*-Alloc-Ala-AsG(Fmoc)-D-Phe-O*t*Bu (**20**) in 86% yield. Removal of the *tert*-butyl ester, provided acid **21**, which coupled to Lys(Boc) resin **11** using DIC and HOBt in DMF. Liberation of *N*-aminosulfamide **23** by removal of the Fmoc protection from AsG(Fmoc) residue was achieved by treating resin **22** with 20% piperidine in DMF. With **23** in hand, alkylation was performed using tetraethylammonium hydroxide in THF as base and five different arylmethyl bromides: benzyl bromide, *p*-fluoro-, *p*-methoxy- and *p*-*tert*-butyldimethylsilyloxybenzyl bromides, and 2-naphthylmethyl bromide. Alkylation conversion to produce mono-alkylated sulfamide **24** was assessed to be between 53–77% by LC-MS analysis of cleaved resin aliquots. Minor amounts of *bis*-alkylated product due likely to alkylation of both sulfamide nitrogen were also detected in 6–12% conversion. Removal of the Alloc protection was performed using palladium tetrakis triphenylphosphine and *N*,*N*-dimethylbarbituric acid (DMBA) in a DCM/DMF mixture. A wash with sodium diethyldithiocarbamate to remove metallic impurities proved important for success in subsequent elongation of the azasulfurylpeptide sequence. After cleavage as described above, azasulfurylpeptides **3a**–**e** were obtained in 61–75% crude purities and isolated by HPLC purification in 3–12% overall yields (Table 1, Supplementary Materials). 

Azasulfuryl-4-fluorophenylalanine peptide **3c** exhibited notable activity (see below) and was prepared on 100-mg scale by a route commencing with alkylation of fluorenylidene *tert*-butyl carbazate (**26**) [33] semicarbazone cleavage with hydroxylamine hydrochloride in pyridine at 60 °C [34] coupling to Fmoc-Ala using *iso*-butyl chloroformate and *N*-methyl morpholine, and Boc group removal using TFA in DCM (Scheme 3). Azasulfuryl-4-fluorophenylalanine tripeptide **31** was then prepared by coupling resulting hydrazide **29** to sulfamidate **30** under microwave irradiation in 53% yield. After cleavage of the *tert*-butyl ester, (4-F)AsF tripeptide **32** was coupled to resin **11** using DIC and HOBt in DMF, and azasulfurylpeptide **3c** was prepared by elongation and cleavage using the protocols described above. In addition, by terminating the sequence with Fmoc-Ala-OH instead of Fmoc-His(Trt)-OH, the importance of the side chain of the terminal residue for activity was evaluated by the synthesis and examination of [Ala^1^, As(4-F)F^4^]-GHRP-6 (**3f**, Table 1). 

### 3.1. Conformational Analysis by Circular Dichroism Spectroscopy

**Figure 2:**
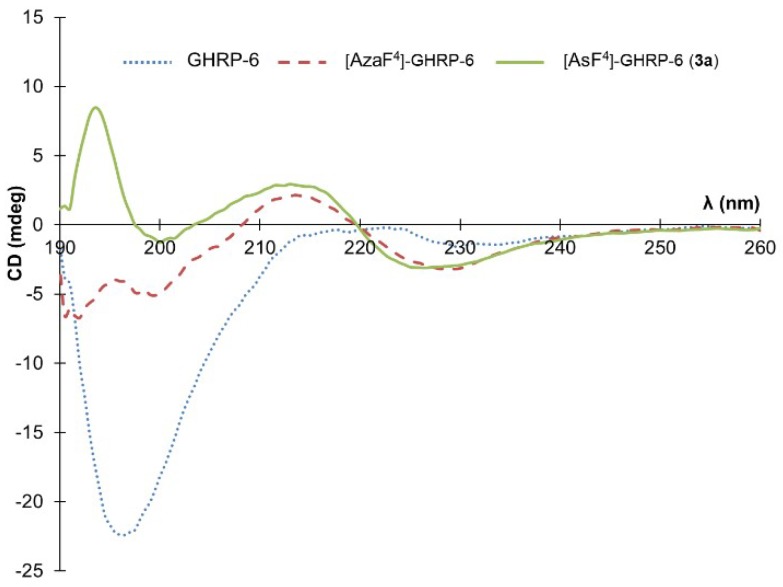
Circular dichroism spectra in water for GHRP-6 (blue dots), [AzaF^4^]-GHRP-6 (red dashes) and [AsF^4^]-GHRP-6 (**3a**, green solid line).

Due to our interest in the effect of the *N*-aminosulfamide residue on the peptide conformation in solution, a circular dichroism (CD) spectroscopic analysis was made to compare GHRP-6, [azaF^4^]-GHRP-6 and [AsF^4^]-GHRP-6 in water (Figure 2). Previously CD spectroscopy showed the parent peptide (e.g., GHRP-6) to adopt a random coil characterized by a spectrum with a negative maximum located around 190 nm. In contrast, [azaF^4^]-GHRP-6 and related azapeptide analogs have exhibited curve shapes indicative of β-turn-like conformations with negative maximum values located around 190 nm and 230 nm and a positive maximum near 215 nm [18].

Examination of the conformation of [AsF^4^]-GHRP-6 (**3a**) by CD spectroscopy in water gave a curve shape like that of its azapeptide counterpart [azaF^4^]-GHRP-6 indicative that the turn conformation may be retained in the azasulfurylpeptide. The positive maximum observed at 194 nm in the spectrum of **3a** deviates however from the ideal β-turn CD spectrum, as well as spectra of other secondary structures [35].

### 3.2. Biology

The six azasulfurylpeptides (e.g., **3a**–**f**) were first examined for the ability to modulate NO overproduction in macrophage cells elicited by a TLR-2 agonist (R-FSL-1). Inhibition of NO production was examined in J774 macrophage cell lines, and compared with the activity of azapeptide counterparts (e.g., **1** and **2**, Table 2). Azapeptides **1** and **2** exhibited comparable NO inhibition activity to that previously reported [15]. Azasulfurylpeptides **3a**–**f** demonstrated significant differences in activity contingent on the 4-position side chain. For example, [AsY^4^]-GHRP-6 (**3b**) exhibited only 0.2-fold of the NO inhibitory effect of [azaY^4^]-GHRP-6 (**1**), which was shown to inhibit significantly (*R*)-fibroblast-stimulating lipopeptide (R-FSL) elicited NO overproduction in macrophages (Table 2). In contrast, azasulfuryl analog [AsF^4^]-GHRP-6 (**3a**) reduced significantly NO production compared to its azapeptide analog [azaF^4^]-GHRP-6 which was shown to display weak inhibitory effect on R-FSL mediated NO release [15]. Moreover, [(4-F)AsF^4^]- and [(4-MeO)AsF^4^]-GHRP-6 (**3c** and **3d**) displayed greater inhibitory effects than their azapeptide counterparts. On the other hand, placement of a bulky naphthylmethyl side chain on the azasulfuryl residue in [2-AsNal]-GHRP-6 (**3e**), and replacement of His by Ala in [Ala^1^, (4-F)AsF^4^]-GHRP-6 (**3f**), both produced analogs with limited inhibitory effect on R FSL-elicited NO production.

Azasulfurylpeptides **3c**–**d** were further examined for their potential to decrease the secretion of the proinflammatory cytokine tumor necrosis factor-α (TNFα) and chemokine monocyte chemoattractant protein-1 (MCP-1) downstream from NF-κB activation by R-FSL in peritoneal macrophages (Table 2). Relative to the effects of azapeptide **1**, pretreatment of macrophages with [(4-F)AsF^4^]-GHRP-6 (**3c**) and [(4-MeO)AsF^4^]-GHRP-6 (**3d**) significantly reduced secretion of TNFα induced by R-FSL by 1.5-fold. Moreover, **3c** significantly reduced the R-FSL-1-induced secretion of MCP-1 by 2.2-fold compared to [azaY^4^]-GHRP-6 (**1**) (*P* < 0.05). 

**Figure 3:**
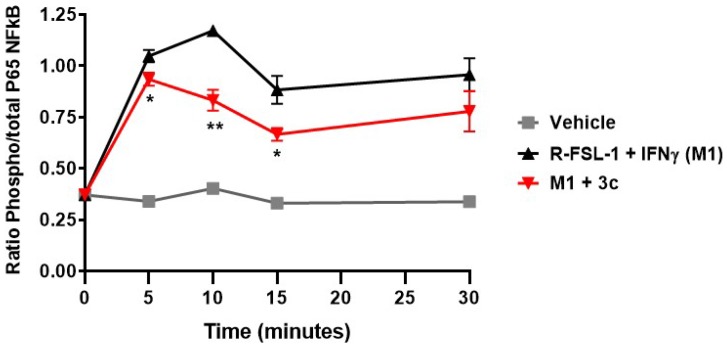
Kinetic profile of NF-κB signaling pathway modulation by azasulfurylpeptide **3c** in peritoneal macrophages, in response to TLR2 ligand (R-FSL-1). * *P* < 0.05, ** *P* < 0.01 compared to R-FSL-1 + IFNγ (M1).

Considering the significant ability of [(4-F)AsF^4^]-GHRP-6 (**3c**) to reduce NO overproduction and cytokine/chemokine release in agonist-stimulated macrophage cells, the kinetic profile of modulation of TLR-2-mediated inflammation through the nuclear factor-κB (NF-κB) pathway was measured in peritoneal macrophages from C57BL/6 mice. After treatment with azasulfurylpeptide **3c** (1 µM), the plated macrophages were stimulated with R-FSL-1 (300 ng/mL) and IFNγ (20 ng/mL) for 0, 5, 10, 15 and 30 min. Notably, azasulfurylpeptide **3c** reduced significantly NF-κB activity elicited by the R-FSL by 11% (*P* < 0.05) at 5 min, by 29% (*P* < 0.01) at 10 min and by 25% (*P* < 0.05) at 15 min after exposure to the TLR2-agonist (Figure 3).

The ability of [(4-F)AsF^4^]-GHRP-6 (**3c**) to compete with the radiolabeled [^125^I]-Tyr-Bpa-Ala-hexarelin used as tracer for CD36 binding was tested on purified membranes from rat heart as a source of CD36 [36]. The binding affinity of azasulfurylpeptide **3c** (IC_50_ 2.53 µM) was 1.3-fold lower and 2.4-fold higher than [azaY^4^]- and [aza(4-F)F^4^]-GHRP-6 (**1** and **2**), respectively [15].

**Figure 4:**
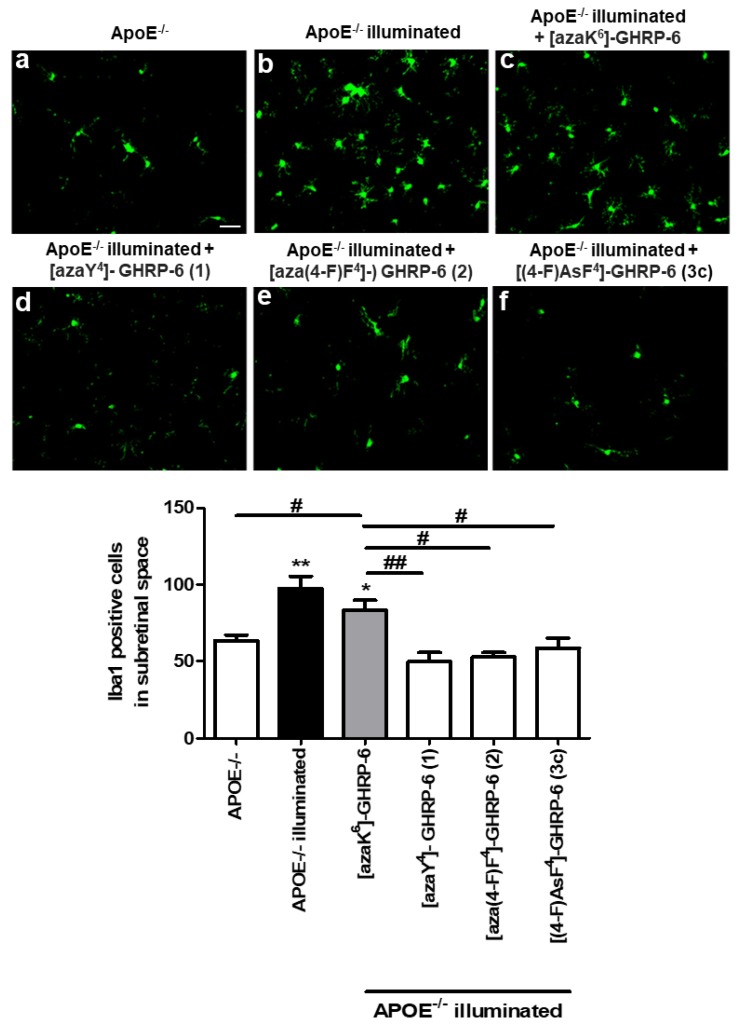
Anti-inflammatory effect of [(4-F)AsF^4^]-GHRP-6 (**3c**), [azaY^4^]- GHRP-6 (**1**), [aza(4-F)F^4^]-GHRP-6 (**2**), and [azaK^6^]-GHRP-6 (negative control) in blue light illumination model. Representative complex RPE/choroid/sclera flat-mounted illustrating the accumulation of positive IBA-1 cells (green) in the subretinal space of ApoE^−/−^ mice non-illuminated, exposed to 1000 Lux blue-light treated or not with CD36 ligands. Quantification of IBA-1 positive cells in the subretinal space of the mouse are presented in histogram (* *P* < 0.05, ** *P* < 0.01 compared to APOE^−/−^, ^#^ *P* < 0.05 ^##^ *P* < 0.01 compared to [azaK^6^]-GHRP-6, *n* = 3–5. Scale bar = 30 μm.

In order to validate the anti-inflammatory activity of azasulfuryl analogs observed in vitro, an in vivo comparative study of azasulfuryl derivative [(4-F)AsF^4^]-GHRP-6 (**3c**) with azapeptides [azaY^4^]- and [aza(4-F)F^4^]-GHRP-6 (**1** and **2**), and [azaK^6^]-GHRP-6 as a negative control, was performed by measuring sub-retinal macrophage and microglia infiltration in a mouse model of retinal inflammation induced by blue-light exposure. Since the apolipoprotein E (ApoE) deficiency was shown to feature a pro-inflammatory phenotype [37,38], ApoE-null mice were used as model [39]. Macrophage infiltration into the retinal pigment epithelium (RPE) and subretinal space are hallmarks of the late phases of age-related macular degeneration, which is the leading cause of adult vision loss [19,40]. Upon illumination of *ApoE*^−/−^ mice with a 1000-lux blue LED-light for 5 day, infiltration of activated macrophages and microglia migrating into the subretinal space was analyzed by IBA-1 staining on RPE flat-mounts. Confirming the inflammatory potential of the ApoE-deficient model, a basal lower accumulation of IBA-1-positive cells was observed in *ApoE*^−/−^ mice that was not dependent on the blue-light intensity (Figure 4) A significant increase of subretinal IBA-1+ cells was observed in the blue light-challenged *ApoE*^−/−^ mice (Figure 4b) relative to their non-illuminated counterparts (Figure 4a) [41]. Compared to illuminated vehicle-treated *ApoE*^−/−^ mice, treatment of blue light-challenged *ApoE*^−/−^ mice with [azaY^4^]-, [aza(4-F)F^4^]-, and [AsF^4^]-GHRP-6 (Figure 4d–f) all decreased significantly (50%) IBA-1+ cell accumulation in the subretina. 

**Figure 5:**
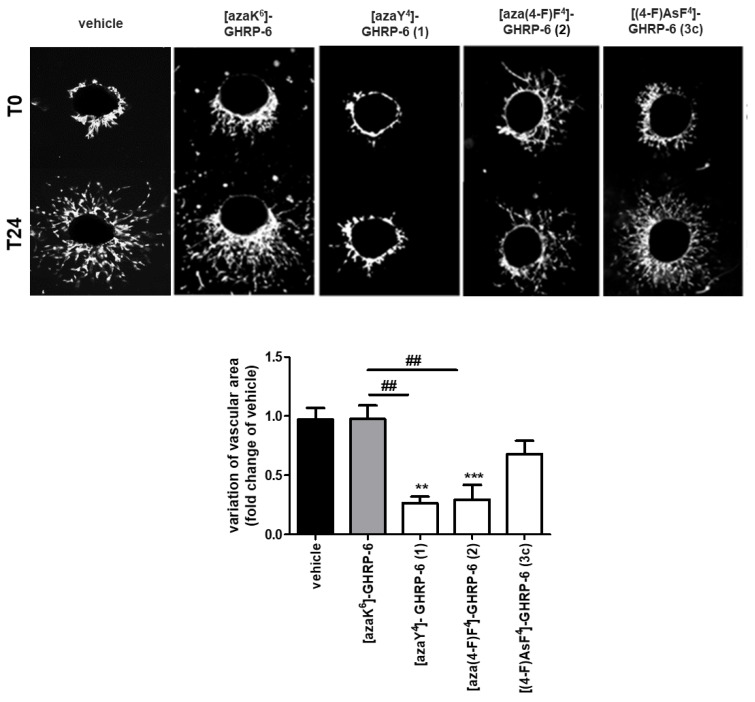
Angiogenic effects of [(4-F)AsF^4^]-GHRP-6 (**3c**), [azaY^4^]- GHRP-6 (**1**) and [aza(4-F)F^4^]-GHRP-6 (**2**) in a mouse choroidal explant model. Representative microvascular sprouting from Matrigel-embedded choroidal explants treated for 24 h with CD36 ligands (10^−6^ M). Histogram represents the quantification of the variation of choroidal sprouting areas according to the ratio (T24−T0)/T0. Values are mean ± SEM ** *P* < 0.01, *** *P* < 0.001 compared to untreated (vehicle), ^##^ *P* < 0.01 compared to [azaK^6^]-GHRP-6 (negative control), *n* = 3–4.

In addition to the oxidative stress and inflammation involved in the development of age-related macular degeneration, CD36-mediated angiogenesis with choroidal neovascularization has been identified as a dominant feature of advanced AMD [42]. Examination of the modulatory effect on angiogenesis of [(4-F)AsF^4^]-GHRP-6 (**3c**) was performed on the RPE/choroid/sclera complex and compared to azapeptides **1** and **2** (Figure 5). We have previously shown that [azaY^4^]- and [aza(4-F)F^4^]-GHRP-6 (**1** and **2**) to be the most effective inhibitors of choroidal vascular sprouting among a set of 25 azapeptides in an evaluation that illustrated the combination of a small electronegative para-substituent on the azaPhe^4^ residue and a His^1^ residue for reducing angiogenic activity [15]. Although featuring structural similarity with azapeptide analogs, [(4-F)AsF^4^]-GHRP-6 (**3c**) did not affect choroidal vascular growth suggesting the requirement of a carbonyl group instead of a sulfuryl moiety for antiangiogenic activity (Figure 5). 

## 4. Conclusions

In the context of research on modulators of the CD36 receptor, a solid-phase method was developed to prepare azasulfurylpeptides. Six [azasulfuryl^4^]-GHRP-6 analogs were synthesized employing azasulfuryltripeptide building blocks that were prepared in solution. Application of the Fmoc group to protect the α-nitrogen of azasulfurylglycine in the tripeptide Alloc-Ala-AsG(Fmoc)-D-Phe-OH (**21**) enabled attachment to the solid support. After Fmoc group removal, alkylation of the *N*-aminosulfamide could be used to introduce a variety of side chains onto the azasulfuryl residue. The power of this novel diversity-oriented solid-phase method for the synthesis of azasulfurylpeptides was demonstrated by the synthesis of five related ligands from a common AsG-peptide.

Examination of the activity of the six azasulfurylpeptides demonstrated that [(4-F)AsF^4^]-GHRP-6 (**3c**) exhibited comparable anti-inflammatory effect as related azapeptides **1** and **2** in a CD36 dependent manner. In macrophages, azasulfurylpeptide **3c** suppressed TLR-agonist-induced overproduction of NO and pro-inflammatory cytokine- and chemokine- production. Similarly, **3c** reduced significantly TLR2-agonist-induced NF-κB in peritoneal macrophages. Moreover, azasulfurylpeptide **3c** exhibited comparable CD36 binding affinity as related azapeptides **1** and **2**. In a mouse model of retinal inflammation, **3c** behaved like **1** and **2** reducing significantly the infiltration of activated/microglia/macrophages into the subretinal space upon photo-oxidative stress. On the other hand, in contrast to azapeptides **1** and **2**, which inhibited microvascular sprouting mediated through CD36 in the mouse choroidal explant model, azasulfurylpeptide **3c** preserved microvascular survival. 

In spite of the structural similarity between azapeptide [aza(4-F)F4]-GHRP-6 (**2**) and azasulfurypeptide [As(4-F)F4]-GHRP-6 (**3c**), the latter did not affect choroidal vascular growth suggesting the requirement for a carbonyl group instead of a sulfuryl moiety for anti-angiogenic activity. Notably, such a structural change influences backbone geometry [22], the Brønsted acidity of the d-Phe NH and the number of Lewis basic oxygens for interaction with the receptor. For modulation of microvascular activity, CD36 has been shown to associate with the Src family of tyrosine protein kinases [43,44]. On binding to CD36, azapeptides have been observed to associate with Fyn kinase and caspase-3 like proteases, inducing apoptotic signaling with a negative effect on angiogenesis [44,45]. In contrast, [As(4-F)F4]-GHRP-6 (**3c**) which displayed no anti-angiogenic effect upon binding to CD36 may associate with Src kinase to promote signaling by way of a vascular endothelial growth factor-driven Akt phosphorylation pathway that ensures microvascular survival [44]. 

Highlighting the delicate relationship between the structure of CD36 ligands and their influences on allosteric modulation of the pleotropic effects mediated by the scavenger receptor, the subtle change from a carbonyl (CO) group in semicarbazide **2** to a sulfuryl (SO_2_) moiety in *N*-aminosulfamide **3c** has obliterated influences on neovascularization without effect on CD36 binding affinity nor CD36-mediated anti-inflammatory activity. As a selective regulator of inflammation, [As(4-F)F^4^]-GHRP-6 (**3c**) represents a novel probe for specifically studying CD36-mediated allosteric modulation of TLR-2 heterodimer complex signaling. Moreover, with capacity to dissociate the antiinflammatory activity from the inhibitory activity on microvascular sprouting, azasulfuryl analog **3c** is a useful prototype for developing CD36 ligands that are devoid of antiangiogenic profile. The conception of azasulfurylpeptide **3c** has thus provided valuable insight into the structural relationships and useful tools for governing CD36 activity that could find their application in the treatment of inflammation underlying ischemic diseases.

## Figures and Tables

**Table 1:** Yields and purities of [azasulfuryl^4^]-GHRP 6 analogs.

Azasulfurylpeptides	Crude Purity (%) ^a^	Isolated Purity (%) ^b^	Isolated Yields (%) ^c^	HRMS	
				*m*/*z* (cal)	*m*/*z* (obs)
His-D-Trp-Ala-AsF-D-Phe-Lys-NH_2_ (**3a**)	73%	>99%	12%	893.3851	893.3832
His-D-Trp-Ala-AsY-D-Phe-Lys-NH_2_ (**3b**)	70%	>99%	9%	909.3801	909.3805
His-D-Trp-Ala-(4-F)AsF-D-Phe-Lys-NH_2_ (**3c**)	65%	>99%	3%	911.3757	911.3769
His-D-Trp-Ala-(4-MeO)AsF-D-Phe-Lys-NH_2_ (**3d**)	75%	>99%	10%	923.3957	923.3936
His-D-Trp-Ala-2-AsNal-D-Phe-Lys-NH_2_ (**3e**)	61%	>99%	10%	943.4008	943.4007
Ala-D-Trp-Ala-(4-F)AsF-D-Phe-Lys-NH_2_ (**3f**)	79%	>99%	23%	845.3647	845.3539

^a^ Crude purities of peptide obtained after an aliquot cleavage of resin using TFA:H_2_O:TES (95:2.5:2.5), followed by LC-MS analysis using 5–80% MeOH (0.1% FA) in H_2_O (0.1% FA) as eluant. ^b^ Isolated purities of peptide obtained by a LC-MS analysis using two separate gradients of MeOH (0.1% FA) in H_2_O (0.1% FA) or MeCN (0.1% FA) in H_2_O (0.1% FA). ^c^ Isolated yields calculated from resin loading.

**Table 2:** In vitro assessment of anti-inflammatory activity of azasulfurylpeptide in macrophages.

Aza-(aza)- and azasulfuryl-(As)-peptides	NO inhibition Fold of 1	TNFα inhibition Fold of 1	MCP-1 inhibition Fold of 1
His-D-Trp-Ala-azaTyr-D-Phe-Lys-NH_2_ (**1**)	1 ^a^	1 ^b^	1 ^c^
His-D-Trp-Ala-aza(4-F)Phe-D-Phe-Lys-NH_2_ (**2**)	1.4	0.87	0.97
His-D-Trp-Ala-AsF-D-Phe-Lys-NH_2_ (**3a**)	1.6	NA	NA
His-D-Trp-Ala-AsY-D-Phe-Lys-NH_2_ (**3b**)	0.2	NA	NA
His-D-Trp-Ala-(4-F)AsF-D-Phe-Lys-NH_2_ (**3c**)	2.2	1.5	2.2
His-D-Trp-Ala-(4-MeO)AsF-D-Phe-Lys-NH_2_ (**3d**)	1.5	1.5	NA
His-D-Trp-Ala-2-AsNal-D-Phe-Lys-NH_2_ (**3e**)	0.06	NA	NA
Ala-D-Trp-Ala-(4-F)AsF-D-Phe-Lys-NH_2_ (**3f**)	-0.04	NT	NT

Against R-FSL-1, [azaY^4^]-GHRP-6 (**1**) inhibited ^a^ NO (31%); [15] ^b^ TNF-α (13%); ^c^ CCL-2 (13%). NA: no activity. NT: not tested.

## Supplementary Materials

Supplementary materials are available online at http://www.mdpi.com/2227-9059/6/4/98/s1.

## Abbreviations

AMD	Age-related macular degeneration
AsF	Azasulfurylphenylalanine
AsG	Azasulfurylglycine
CD36	Cluster of differentiation
GHRP-6	Growth hormone releasing peptide-6
HIV-1	Human immunodeficiency virus-1 proteinase
LTA	lipoteichoic acid
NF-κB	nuclear factor kappa-light-chain-enhancer of activated B cells
NO	Nitric oxide
FSL	Fibroblast-stimulating lipopeptide
MCP-1	monocyte chemotactic protein-1
TLR	Toll-Like Receptor
TNFα	Tumor necrosis factor alpha
