# Supplementary material for: Azasulfurylpeptide Modulation of CD36-Mediated Inflammation Without Effect on Neovascularization
Source: Biomedicines. 2018 Oct 22;6(4):98. doi: 10.3390/biomedicines6040098 (PMC6315388; doi:10.3390/biomedicines6040098)
Supplement: Supplementary file 1 [file biomedicines-06-00098-s001.pdf]

Supporting Information

Azasulfurylpeptide Modulation of CD-36 Mediated Inflammation Without Effect on Neovascularization

Stéphane Turcotte,[1,§] Katia Mellal,[2,§] Ramesh Chingle,[1] Mukandila Mulumba,[2] Samy Omri,[3] Lylia Dif-Yaiche,[1] Sylvain Chemtob[3,4,] Huy Ong,[2,*] William D. Lubell[1,*]

[1]Departments of Chemistry, [2]Faculty of Pharmacy, [4]Pediatrics, Ophthalmology and Pharmacology, Université de Montréal, Montréal, Canada

[3]Maisonneuve-Rosemont Hospital, Montréal, Canada

§ Equal contribution

* Correspondence: william.lubell@umontreal.ca; Tel.: +1-514-343-7339

* Equal contribution

huy.ong@umontreal.ca; *william.lubell@umontreal.ca*

Table of Contents

Solution Phase Synthesis 2
General Methods 2
tert-Butyl 3-fluorenylidene *N*-(4-fluorobenzyl)carbazate (**27**) 2
tert-Butyl *N*-(4-fluorobenzyl)carbazate (**28**) 2
N-(Fmoc)Alanine *N*'-(Boc)hydrazide (**SI-1**) 3
N-(Alloc)Alanine *N*'-(Boc)hydrazide (**SI-2**) 3
N-(Fmoc)-Alanine Hydrazide (**SI-3**) 3
N-(Alloc)Alanine hydrazidium trifluoroacetate (**SI-4**) 3
N-(Fmoc)Alaninyl-azasulfurylglycinyl-D-phenylalanine *tert*-Butyl Ester (**SI-5**) 4
N-(Fmoc)-Alaninyl-azasulfurylglycinyl-D-phenylalanine (**14**) 4
N-(Alloc)Alaninyl-azasulfurylglycinyl-D-phenylalanine *tert*-Butyl Ester (**19**) 4
N-(Alloc)Alaninyl-azasulfurylglycinyl-D-phenylalanine (**17**) 4
N-(Alloc)Alaninyl-(2-amino-(*R*)-4-benzyl)sulfahydantoin (**18**) 5
H-Lys(Boc)-NH-Rink resin (**11**) 5
4-(TBDMSO)benzaldehyde (**SI-6**) 5
4-(TBDMSO)benzyl alcohol (**SI-7**) 5
4-(TBDMSO)benzyl bromide (**SI-8**) 6

Solution Phase Synthesis

General Methods

4-Nitrophenyl D-phenylalanine *tert*-butyl ester sulfamidate,[20] *N*-(Alloc)alanine,[20] fluorenone hydrazone,[31] and *tert*-butyl 3-fluorenylidene carbazate,[31] all were synthesized according to literature methods; references concord with the publication text. *Iso*-butyl chloroformate, 4-methylmorpholine, *tert*-butyl carbazate, potassium carbonate, sodium carbonate, 4-fluorobenzyl bromide, triethylamine, 40% tetraethylammonium hydroxide in water, all were purchased from Aldrich® and used as received. Benzyl bromide was purchased from Aldrich® and filtered through a small plug of silica gel prior to use. Fmoc-Ala-OH was purchased from GL Biochem® (Shanghai, China) Ltd. 1,2-Dichloroethane (DCE), trifluoroacetic acid (TFA), 1,4-dioxane, Fmoc-OSu, sulfuric acid and *tert*-butyl acetate were respectively purchased from Aldrich®, A&C Chemicals®, J. T. Baker®, GenScript® Corporation, A&C Chemicals® and Aldrich®, and used as received. Anhydrous solvents [tetrahydrofuran (THF) and dichloromethane (DCM)] were obtained by passage through a solvent filtration system (GlassContour®, Irvine, CA). Ethyl acetate (EtOAc) and hexanes were purchased from Fisher Chemical® and fractionally distilled prior to use. Microwave irradiation was accomplished using a 300 MW Biotage® apparatus on the high-absorption level; temperature was monitored automatically. Flash chromatography was performed (according to Still, W. C.; Kahn, M.; Mitra, A. *J. Org. Chem.* **1978**, *43*, 2923) on 230–400 mesh silica gel, and thin-layer chromatography was performed on silica gel 60 F254 plates from Merck®. Melting points were measured using a Gallankamp® apparatus and are uncorrected. Specific rotations, $[\alpha]_D$ values, were calculated from optical rotations measured at 20 °C in $CHCl_3$ or MeOH at the specified concentrations (*c* in g/100 mL) using a 1-dm cell (*l*) on a PerkinElmer Polarimeter 341, using the general formula: $[\alpha]^{20}_D = (100 \times \alpha)/(l \times c)$. Accurate mass measurements were performed on a LC-MSD instrument from Agilent technologies in positive electrospray ionisation (ESI) mode at the Université de Montréal Mass Spectrometry facility. Sodium and proton adducts {$[M+Na]^+$ and $[M+H]^+$} were used for empirical formula confirmation. ^1H NMR spectra were measured in $CDCl_3$ (7.26 ppm) or CD_3OD (3.34 ppm) or DMSO-d_6 (2.5 ppm). ^{13}C NMR spectra were measured in $CDCl_3$ (77.36 ppm) or CD_3OD (49.86 ppm) or DMSO-d_6 (39.52 ppm). When distinguishable, proton and carbon resonances for minor isomer are respectively reported in brackets and parentheses. Coupling-constant *J* values are measured in Hertz (Hz) and chemical shift values are reported in parts per million (ppm). Infrared spectra were recorded in the neat on an ATR Bruker® apparatus.

***tert*-Butyl 3-fluorenylidene *N*-(4-fluorobenzyl)carbazate (27)**

A solution of *tert*-Butyl 3-fluorenylidene carbazate (**26**, 3 g, 10.2 mmol, prepared according to reference 31) in 40 mL of anhydrous THF at 0 °C was treated with 40% tetraethylammonium hydroxide in H_2O (5.49 mL, 15.3 mmol), stirred for 30 min, treated with 4-fluorobenzyl bromide (3.26 mL, 15.3 mmol), allowed to warm to room temperature (rt), stirred for 16 h, diluted with CH_2Cl_2, washed with H_2O (2 X), dried with $MgSO_4$, filtered, and evaporated. The residue was purified by flash chromatography using 1:4 EtOAc:hexane as solvent system. Evaporation of the collected fractions gave carbazate **27** as yellow solid (2.56 g, 52% yield): *Rf* 0.61 (4:1 hexane/EtOAc); mp 140 °C; ^1H NMR ($CDCl_3$, 400 MHz) δ 1.31 (9H, s), 4.99 (2H, s), 7.10-7.30 (4H, m), 7.30-7.45 (4H, m), 7.51 (1H, d, *J* = 11.0), 7.54 (1H, d, *J* = 11.1), 7.73 (1H, d, *J* = 10.2), 7.84 (1H, d, *J* = 10.0); ^{13}C NMR ($CDCl_3$, 75 MHz) δ: 28.5, 56.3, 82.1, 120.1 (d, $J\text{-}^{19}F$ = 22.5) 123.4, 127.8 (d, $J\text{-}^{19}F$ = 41.0), 128.43, 128.45, 128.6, 131.58, 131.85 (d, $J\text{-}^{19}F$ = 41.1), 137.1, 139.9 (d, $J\text{-}^{19}F$ = 214.5), 142.9, 152.9; HRMS (ESI) m/z calculated for $C_{25}H_{24}FN_2O_2$ $[M+H]^+$ 403.1744; found 403.1816.

***tert*-Butyl *N*-(4-fluorobenzyl)carbazate (28)**

tert-Butyl *N*-(4-fluorobenzyl)fluorenylidene carbazate (**27**, 2.52 g, 6.27 mmol) was treated with a solution of $NH_2OH{\cdot}HCl$ (1.7 g, 25.11 mmol) in pyridine (17 mL) at 60 °C for 12 h. The volatiles were evaporated and the residue was purified by flash chromatography eluting with 1:9 EtOAc:Hexane. Carbazate **28** was obtained as an oil (1.08 g, 72% yield): *Rf* 0.1 (hexane:EtOAc 4:1); ^1H NMR ($CDCl_3$, 400 MHz) δ 1.46 (9H, s), 3.96 (2H, br), 4.48 (2H, s), 6.90-7.05 (2H, m), 7.15-7.30 (2H, m); ^{13}C NMR ($CDCl_3$, 75 MHz) δ 29.0, 54.0, 81.2, 115.6 (d, $J\text{-}^{19}F$ = 21.2), 129.9 (d, $J\text{-}^{19}F$ = 8.0), 134.1 (d, $J\text{-}^{19}F$ = 3.2), 157.0, 162.5 (d, $J\text{-}^{19}F$ = 243.8); HRMS (ESI) m/z calculated for $C_{12}H_{18}FN_2O_2$ $[M+H]^+$ 241.1274; found 241.1346.

N-(Fmoc)Alanine N'-(Boc)hydrazide (SI-1)

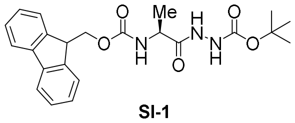

SI-1

A solution of N-(Fmoc)alanine (1.87 g, 6.00 mmol) in dry THF (35 mL) at –15 °C was treated with iso-butyl chloroformate (780 μL, 6.00 mmol) and 4-methylmorpholine (820 μL, 15.0 mmol), stirred for 15 min, treated with a solution of *tert*-butyl carbazate (0.66 g, 5.00 mmol) in dry THF (5 mL), and stirred for 2h. The volatiles were evaporated. The residue was dissolved in DCM (100 mL), washed with water (2 x 100 mL), dried over MgSO4, filtered and evaporated. The residue was purified by flash chromatography eluting with 1:1 hexane:EtOAc to afford *N*-(Fmoc)alanine *N'*-(Boc)-hydrazide as a solid (1.94 g, 91%): R_f 0.32 (hexane:EtOAc 1:1); mp 73 °C; $[\alpha]^{20}_{D}$ –23.9° ($CHCl_3$, *c* 1.04); ^1H NMR ($CDCl_3$, 400 MHz) δ 1.35-1.50 (12H, m), 4.10-4.20 (1H, m), 4.30-4.40 (3H, m), 5.79 (1H, br), 6.82 (1H, br), 7.20-7.30 (2H, m), 7.30-7.40 (2H, m), 7.50-7.60 (2H, m), 7.70-7.80 (2H, m), 8.64 (1H, br); ^{13}C NMR ($CDCl_3$, 100 MHz) δ 18.7, 28.4, 47.3, 49.3, 67.5, 82.1, 120.3, 125.4, 127.4, 128.0, 141.6, 144.0, 155.7, 156.5, 172.6; IR (neat) ν_{max}/cm^{-1} 1161, 1245, 1368, 1450, 1531, 1695, 2979, 3290; HRMS (ESI) m/z calculated for $C_{23}H_{27}N_3NaO_5$ $[M+Na]^+$ 448.1842; found 448.1836.

N-(Alloc)Alanine N'-(Boc)hydrazide (SI-2)

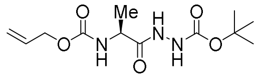

SI-2

N-(Alloc)Alanine *N'*-(Boc)hydrazide was synthesized using the same protocol above from *N*-(Alloc)alanine (2.08 g, 12.0 mmol), *iso*-butyl chloroformate (1.57 mL, 12.0 mmol) and 4-methylmorpholine (1.65 mL, 15.0 mmol) in dry THF (70 mL), and purified by flash chromatography eluting with 11:9 hexane:EtOAc to afford a solid (2.04 g, 71%): R_f 0.19 (hexane:EtOAc 3:2); mp 54 °C; $[\alpha]^{20}_{D}$ –36.8° ($CHCl_3$, *c* 1.06); ^1H NMR ($CDCl_3$, 400 MHz) δ 1.41 (3H, d, *J* = 7.1), 1.45 (9H, s), 4.25-4.35 (1H, m), 4.50-4.60 (2H, m), 5.21 (1H, dq, *J* = 1.2, 10.4), 5.30 (1H, dq, *J* = 1.5, 17.2), 5.53 (1H, br), 5.80-5.95 (1H, m), 6.69 (1H, br), 8.42 (1H, br); ^{13}C NMR ($CDCl_3$, 100 MHz) δ 18.7, 28.4, 49.2, 66.2, 81.9, 118.2, 132.7, 155.8, 156.4, 172.8; IR (neat) ν_{max}/cm^{-1} 1156, 1236, 1367, 1452, 1501, 1679, 2978, 3279; HRMS (ESI) m/z calculated for $C_{12}H_{21}N_3NaO_5$ $[M+Na]^+$ 310.1373; found 310.1377.

N-(Fmoc)-Alanine Hydrazide (SI-3)

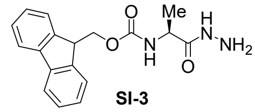

SI-3

N-(Fmoc)Alanine *N'*-(Boc)-hydrazide (399 mg, 0.94 mmol) was treated with TFA:DCM 1:1 (1 mL) at rt for 1 h. The volatiles were evaporated, the residue was dissolved in DCM and co-evaporated several times to remove TFA. The residue was partitioned between sat. $NaHCO_3$ (20 mL) and $CHCl_3$ (20 mL), and the aqueous phase was extracted with $CHCl_3$ (4 x 20 mL). The combined organic layers were dried over $MgSO_4$, filtered and evaporated to afford *N*-(Fmoc)alanine hydrazide as a solid (302 mg, 99%), that was used without further purification: R_f 0.20 (hexane:EtOAc 3:7); mp 141 °C; $[\alpha]^{20}_{D}$ –15.3° (THF, *c* 0.68); ^1H NMR (DMSO-d_6, 400 MHz) δ 1.24 (3H, d, *J* = 7.1), 4.00-4.10 (1H, m), 4.20-4.35 (5H, m), 7.30-7.40 (2H, m), 7.45 (2H, t, *J* = 7.2), 7.53 (1H, d, *J* = 7.8), 7.77 (2H, d, *J* = 6.8), 7.92 (2H, d, *J* = 7.5), 9.11 (1H, s); ^{13}C NMR (DMSO-d_6, 100 MHz) δ 18.4, 46.6, 48.8, 65.6, 120.1, 125.3, 127.1, 127.6, 140.7, 143.8, 143.9, 155.6, 171.8. IR (neat) ν_{max}/cm^{-1} 1104, 1251, 1323, 1381, 1537, 1660, 1655, 1688, 2974, 3303; HRMS (ESI) m/z calculated for $C_{18}H_{19}N_3NaO_3$ $[M+Na]^+$ 348.1319; found 348.1318.

N-(Alloc)Alanine hydrazidium trifluoroacetate (SI-4)

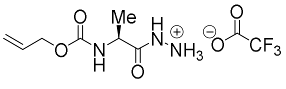

SI-4

N-(Alloc)Alanine hydrazidium trifluoroacetate was synthesized using the above protocol as an oil (1.49 g, 99%): $[\alpha]^{20}_D$ –66.5° (MeOH, *c* 0.92); ^{1}H NMR (CD_3OD, 400 MHz) showed an 1:1 mixture of hydrazide salt isomers:[1] δ 1.417 (3H, d, *J* = 7.2) [1.419 (3H, d, *J* = 7.2)], 4.20-4.30 (1H, m), 4.50-4.60 (2H, m), 5.20 (1H, dq, *J* = 1.3, 10.5), 5.33 (1H, dq, *J* = 1.4, 17.2), 5.90-6.00 (1H, m); ^{13}C NMR (CD_3OD, 100 MHz)[6] δ 19.2 (18.6) 51.5, 67.5 (67.6), 118.2 (d, $J\text{-}^{19}F$ = 287.9), 118.54 (118.60), 135.05 (134.95), 158.9 (159.0), 162.5 (d, $J\text{-}^{19}F$ = 36.9), 175.1 (172.2). IR (neat) ν_{max}/cm^{-1} 1129, 1179, 1201, 1249, 1450, 1521, 1655, 3270; HRMS (ESI) m/z calculated for $C_7H_{14}N_3O_3$ $[M]^+$ 188.1030; found 188.1033.

N-(Fmoc)Alaninyl-azasulfurylglycinyl-D-phenylalanine *tert*-Butyl Ester (SI-5)

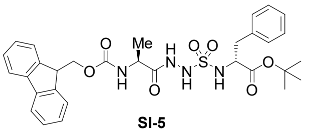

SI-5

A solution of 4-nitrophenyl D-phenylalanine *tert*-butyl ester sulfamidate (976 mg, 2.31 mmol) in THF (10.0 mL) was added to *N*-(Fmoc)alanine hydrazide (976 mg, 3.00 mmol) in a microwave vessel. The mixture was treated with DIEA (517 µL, 3.00 mmol), at which point the solution turned yellow. Gentle heating with a heat gun may be required to facilitate dissolution. The vessel was sealed and heated to 60 °C using microwave irradiation for 2.5 h. The volatiles were then evaporated and the residue was purified by flash chromatography eluting with 3:2 hexane:EtOAc. The collected fractions were combined and evaporated to a residue that was dissolved in DCM (25 mL). The organic phase was washed with sat. $NaHCO_3$ (3 x 25 mL), dried over $MgSO_4$, filtered and evaporated to afford Fmoc-Ala-AsG-D-Phe-O*t*-Bu as a solid (1.22 g, 87%): R_f 0.24 (hexane:EtOAc 3:2); mp 82 °C; $[\alpha]^{20}_D$ 48.9° ($CHCl_3$, *c* 0.79); ^{1}H NMR ($CDCl_3$, 400 MHz) δ 1.35-1.45 (12H, m), 3.09 (1H, dd, *J* = 7.4, 13.9), 3.15 (1H, dd, *J* = 5.6, 14.0), 4.15-4.25 (1H, m), 4.30-4.45 (4H, m), 5.65 (1H, d, *J* = 7.4), 5.77 (1H, d, *J* = 6.9), 7.20-7.35 (8H, m), 7.42 (2H, t, *J* = 7.4), 7.61 (2H, t, *J* = 7.5), 7.78 (2H, d, *J* = 7.5), 8.64 (1H, s); ^{13}C NMR ($CDCl_3$, 100 MHz) δ 18.2, 28.2, 39.1, 47.3, 49.4, 57.6, 67.6, 83.5, 120.2, 125.4, 127.4, 128.0, 128.7, 130.1, 135.9, 141.6, 143.9, 144.1, 156.5, 171.2, 172.3; IR (neat) ν_{max}/cm^{-1} 1105, 1154, 1251, 1367, 1451, 1523, 1709, 2979, 3065, 3275; HRMS (ESI) m/z calculated for $C_{31}H_{36}N_4NaO_7S$ $[M+Na]^+$ 631.2197; found 631.2187.

N-(Fmoc)-Alaninyl-azasulfurylglycinyl-D-phenylalanine (14)

Acid **10** was synthesized from Fmoc-Ala-AsG-D-Phe-O*t*-Bu (150 mg, 0.25 mmol) as described for **10**, which afforded **14** as a solid (135 mg, 99%): R_f 0.17 (hexane:EtOAc 3:7); mp 185 °C; $[\alpha]^{20}_D$ –15.3° ($CHCl_3$, *c* 0.84); ^{1}H NMR ($CDCl_3$, 400 MHz) δ 1.24 (3H, d, *J* = 6.3), 3.00-3.15 (2H, m), 4.05-4.15 (1H, m), 4.20-4.25 (1H, m), 4.25-4.35 (2H, m), 4.41 (1H, t, *J* = 5.1), 5.78 (1H, br), 5.89 (1H, br), 7.00-7.15 (6H, m), 7.21 (2H, t, *J* = 7.4), 7.32 (2H, t, *J* = 7.4), 7.45-7.60 (2H, m), 7.68 (2H, d, *J* = 7.6), 8.76 (1H, br); ^{13}C NMR ($CDCl_3$, 100 MHz) δ 18.2, 38.8, 47.2, 49.4, 56.9, 67.9, 120.3, 125.4, 127.4, 128.1, 128.9, 130.0, 135.3, 141.5, 143.7, 144.0, 157.0, 173.4, 174.3; IR (neat) ν_{max}/cm^{-1} 1161, 1239, 1261, 1353, 1457, 1525, 1689, 3221; HRMS (ESI) m/z calculated for $C_{27}H_{29}N_4O_7S$ $[M+H]^+$ 553.1752; found 553.1750.

N-(Alloc)Alaninyl-azasulfurylglycinyl-D-phenylalanine *tert*-Butyl Ester (19)

N-(Alloc)Alaninyl-azasulfurylglycinyl-D-phenylalanine *tert*-Butyl Ester (**19**) was synthesized from *N*-(Alloc)alanine hydrazidium trifluoroacetate (563 mg, 1.87 mmol) using the protocol above with sulfamidate (718 mg, 1.70 mmol), NEt_3 (496 µL, 3.57 mmol) in DCE (7.5 mL), purified by flash chromatography eluting with 3:2 hexane:EtOAc, to afford a solid (557 mg, 70%): R_f 0.33 (hexane:EtOAc 3:2); mp 52 °C; $[\alpha]^{20}_D$ –70.9° ($CHCl_3$, *c* 1.00); ^{1}H NMR ($CDCl_3$, 400 MHz) δ 1.36 (3H, d, *J* = 7.1), 1.39 (9H, s), 3.06 (1H, dd, *J* = 6.6, 13.9), 3.11 (1H, dd, *J* = 5.5, 13.9), 4.20-4.30 (2H, m), 4.45-4.60 (2H, m), 5.19 (1H, dd, *J* = 1.2, 10.4), 5.27 (1H, dd, *J* = 1.2, 17.2), 5.51 (1H, d, *J* = 6.9), 5.64 (1H, d, *J* = 7.8), 5.80-5.95 (1H, m), 7.20-7.30 (6H, m), 8.56 (1H, s); ^{13}C NMR ($CDCl_3$, 100 MHz) δ 18.2, 28.2, 39.1, 49.5, 57.6, 66.5, 83.5, 118.4, 127.4, 128.7, 130.1, 132.7, 135.9, 156.4, 171.2, 172.4; IR (neat) ν_{max}/cm^{-1} 1150, 1249, 1364, 1454, 1515, 1688, 2977, 3219; HRMS (ESI) m/z calculated for $C_{20}H_{30}N_4NaO_7S$ $[M+Na]^+$ 493.1727; found 493.1728.

N-(Alloc)Alaninyl-azasulfurylglycinyl-D-phenylalanine (17)

N-(Alloc)Alaninyl-azasulfurylglycinyl-D-phenylalanine (**17**) was synthesized as described for **10** from *tert*-butyl ester **19** (100 mg, 0.21 mmol), which afforded a solid (87 mg, 99%): mp 51 °C; $[\alpha]^{20}_D$ –62.4° ($CHCl_3$, *c* 1.00);

^{1}H NMR ($CDCl_3$, 400 MHz δ 1.33 (3H, d, *J* = 6.5), 3.05-3.25 (2H, m), 4.20-4.35 (1H, m), 4.35-4.50 (2H, m), 4.52 (1H, dd, *J* = 5.1, 12.9), 5.18 (1H, d, *J* = 10.4), 5.27 (1H, d, *J* = 17.2), 5.80-5.90 (2H, m), 5.95-6.05 (1H, br), 7.15-7.30 (5H, m), 7.50-7.70 (1H, br), 8.00-8.20 (1H, br), 8.90 (1H, s); ^{13}C NMR ($CDCl_3$, 100 MHz) δ 17.9, 38.9, 49.8, 57.1, 66.9, 118.8, 127.6, 128.9, 130.0, 132.4, 135.4, 157.1, 173.8, 174.5; IR (neat) v_{max}/cm^{-1} 1157, 1215, 1343, 1455, 1521, 1678, 3249; HRMS (ESI) m/z calculated for $C_{16}H_{22}N_4NaO_7S$ $[M+Na]^+$ 437.1101; found 437.1092.

_N_-(Alloc)Alaninyl-(2-amino-(_R_)-4-benzyl)sulfahydantoin (18)

A solution of AsG-peptide **17** (70 mg, 0.17 mmol) in THF (1 mL) was treated with DIC (26.3 μL, 0.17 mmol) at rt for 18h. The precipitate was filtered through a plastic syringe filter and the filtrate was evaporated to a residue that was purified by flash chromatography eluting with 2:3 EtOAc:Hexane to afford sulfahydantoin **18** (38 mg; 57%): R_f 0.23 (EtOAc;Hexane 2:3); mp 72 °C; $[\alpha]^{20}_D$ 43.0° ($CHCl_3$, *c* 1.17); ^{1}H NMR ($CDCl_3$, 500 MHz δ 1.42 (3H, d, *J* = 6.2), 3.15 (1H, dd, *J* = 10.5, 14.4), 3.33 (1H, dd, *J* = 3.8, 14.5), 4.40-4.65 (4H, m), 5.22 (1H, dd, *J* = 0.9, 10.4), 5.30 (1H, dd, *J* = 1.3, 17.2), 5.54 (1H, d, *J* = 7.2), 5.80-5.95 (1H, m), 6.15 (1H, d, *J* = 6.6), 7.25-7.30 (3H, m), 7.30-7.35 (2H, m), 9.23 (1H, s); ^{13}C NMR ($CDCl_3$, 125 MHz) δ 18.0, 36.8, 49.2, 61.1, 66.8, 118.7, 127.8, 129.2, 129.7, 132.4, 135.6, 156.8, 167.1, 172.1; IR (neat) v_{max}/cm^{-1} 1189, 1240, 1346, 1455, 1498, 1690, 1765, 2949, 3673; HRMS (ESI) m/z calculated for $C_{16}H_{20}N_4NaO_6S$ $[M+Na]^+$ 419.0996; found 419.1013.

H-Lys(Boc)-NH-Rink resin (11)

Fmoc-Rink resin (**17**, 1.25 g, 0.64 mmol / g, 0.80 mmol or 1.00 g, 1.00 mmol / g, 1.00 mmol) was swollen in DMF (8 mL) in a 12 mL plastic filtration tube with a polyethylene filter, treated with 20% piperidine in DMF (8 mL) to remove the Fmoc group, coupled to Fmoc-Lys(Boc)-OH (respectively 1.12 g, 2.40 mmol or 1.41 g, 3.00 mmol) using HBTU (respectively 0.91 g, 2.40 mmol or 1.14 g, 3.00 mmol) and DIEA (respectively 0.83 mL, 4.80 mmol or 1.00 mL, 6.00 mL) as described for the synthesis of **3a**. After washing the resin with DMF (3 x 8 mL), MeOH (3 x 8 mL) and DCM (3 x 8 mL), the resin was treated with 20% piperidine in DMF (8 mL) to remove the Fmoc group, which afforded resin **11**.

4-(TBDMSO)benzaldehyde (SI-6)

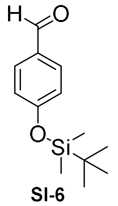

SI-6

Imidazole (5.11 g, 75.0 mmol) was dissolved in dry DCM (250 mL) in a 500 mL flamed dried flask under argon, cooled to 0 °C, treated with 4-hydroxybenzaldehyde (5.00 g, 40.9 mmol), followed by tert-butyldimethylsilyl chloride (6.78 g, 45.0 mmol), and allowed to warm to room temperature. After stirring for 18 h, the mixture was washed with water (3 x 100 mL). The organic layer was dried over $MgSO_4$, filtered and evaporated to a residue, that was purified by flash chromatography eluting with hexane:EtOAc 19:1 to afford 4-(TBDMSO)benzaldehyde as an oil (8.38 g, 87%): R_f 0.68 (hexane:EtOAc 4:1); ^{1}H NMR ($CDCl_3$, 400 MHz) δ 0.27 (6H, s), 1.02 (9H, s), 6.97 (2H, d, *J* = 8.3), 7.81 (2H, d, *J* = 8.3), 9.91 (1H, s); ^{13}C NMR ($CDCl_3$, 100 MHz) δ –4.0, 18.6, 25.9, 120.8, 130.7, 132.2, 161.8, 191.2; IR (neat) v_{max}/cm^{-1} 1157, 1212, 1275, 1473, 1508, 1599, 1698, 2859, 2933; HRMS (ESI) m/z calculated for $C_{13}H_{21}O_2Si$ $[M+H]^+$ 237.1305; found 237.1314.

4-(TBDMSO)benzyl alcohol (SI-7)

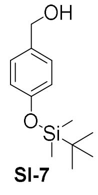

SI-7

4-(TBDMSO)Benzaldehyde (3.00 g, 12.7 mmol) was dissolved in EtOH (25 mL), cooled to 0 °C, and treated with $NaBH_4$ (0.96 g, 25.4 mmol). After stirring for 2 h, the solution was poured slowly into 150 mL of 1 N HCl at 0 °C. The aqueous solution was then extracted with Et_2O (4 x 150 mL) and the organic phase was dried

over $MgSO_4$, filtered and evaporated to a residue. The crude residue was purified by flash chromatography eluting with hexane: EtOAc 4:1 to afford 4-(TBDMSO)benzyl alcohol as an oil (2.68 g, 88%): R_f 0.32 (Hexane:EtOAc 4:1); ^{1}H NMR ($CDCl_3$, 400MHz) δ 0.24 (6H, s), 1.03 (9H, s), 2.43 (1H, br) 4.58 (2H, s), 6.86 (2H, d, *J* = 8.1), 7.23 (2H, d, *J* = 8.1); ^{13}C NMR ($CDCl_3$, 100 MHz) δ −4.1, 18.5, 26.0, 65.2, 120.4, 128.8, 134.0, 155.5; IR (neat) $ν_{max}$/cm^{-1} 1254, 1472, 1510, 1610, 2857, 2930, 3339; HRMS (ESI) m/z calculated for $C_{13}H_{23}O_2Si$ $[M+H]^+$ 239.1462; found 239.1457.

4-(TBDMSO)benzyl bromide (SI-8)

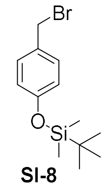

SI-8

Phosphorus tribromide (35 μL, 0.38 mmol) was dissolved in dry DCM (30 mL), cooled to 0 °C, and treated drop-wise with a solution of 4-(TBDMSO)benzyl alcohol (180 mg, 0.76 mmol) in dry DCM (40 mL). After stirring at 0 °C for 30 min, the mixture was filtered through a small plug of silica gel to afford benzyl bromide **51** as an oil (215 mg, 95%: R_f 0.77 (hexane: EtOAc, 9:1); ^{1}H NMR ($CDCl_3$, 400 MHz) δ 0.26 (6H, s), 1.04 (9H, s), 4.53 (2H, s), 6.85 (2H, d, *J* = 8.5), 7.31 (2H, d, *J* = 8.5); ^{13}C NMR ($CDCl_3$, 100 MHz) δ −4.1, 18.5, 26.0, 34.3, 120.6, 130.7, 130.8, 156.2; IR (neat) $ν_{max}$/cm^{-1} 1269, 1511, 1608, 2859, 2929, 2953.

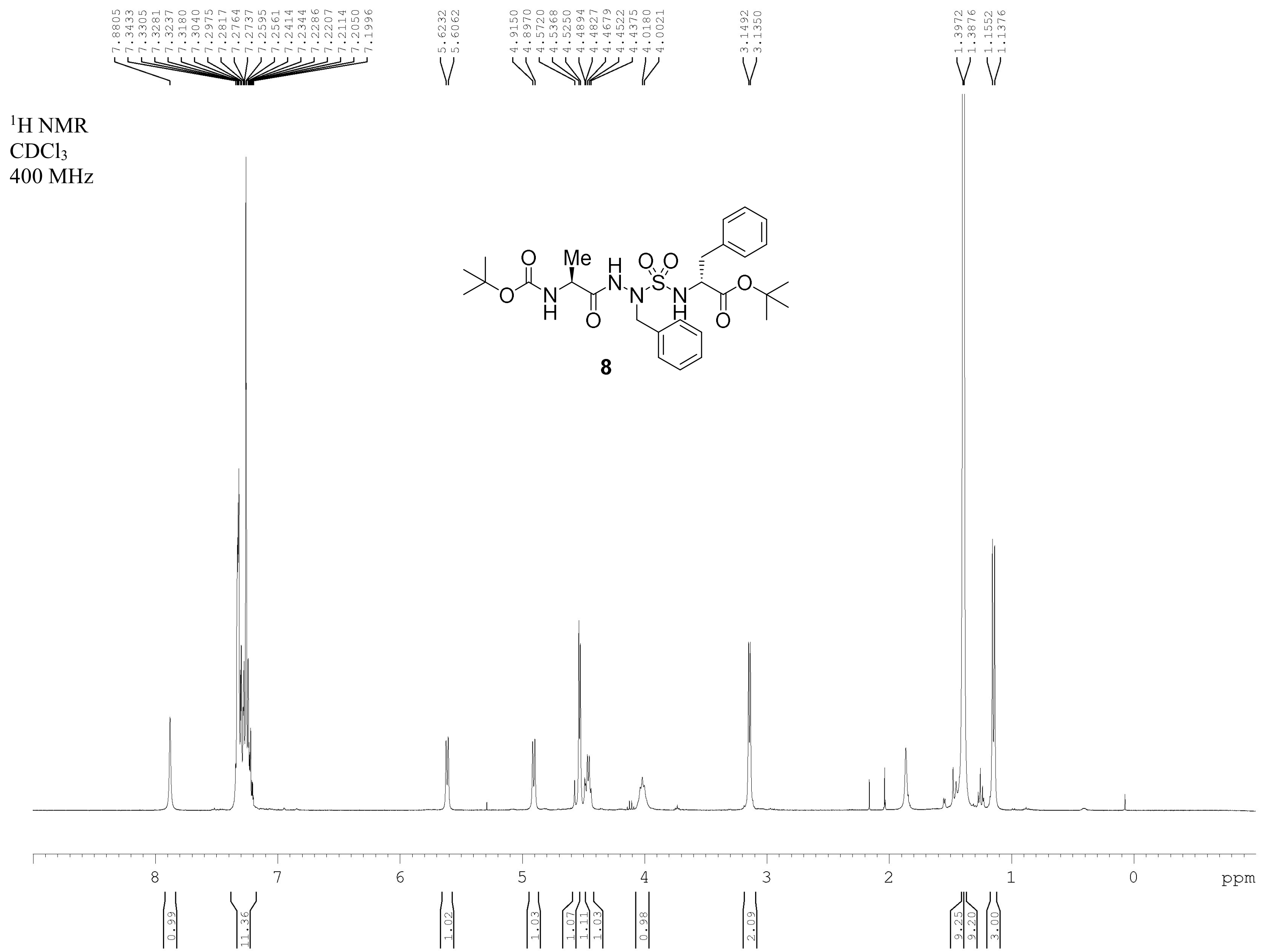
7.8805
7.3433
7.3305
7.3281
7.3237
7.3180
7.3040
7.2975
7.2817
7.2764
7.2737
7.2595
7.2561
7.2414
7.2344
7.2286
7.2207
7.2114
7.2050
7.1996
5.6232
5.6062
4.9150
4.8970
4.5720
4.5368
4.5250
4.4894
4.4827
4.4679
4.4522
4.4375
4.0180
4.0021
3.1492
3.1350
1.3972
1.3876
1.1552
1.1376
1H NMR
CDCl3
400 MHz
Me
O
H
N
S
8
ppm
0.99
11.36
1.02
1.03
1.07
1.11
1.03
0.98
2.09
9.25
9.20
3.00

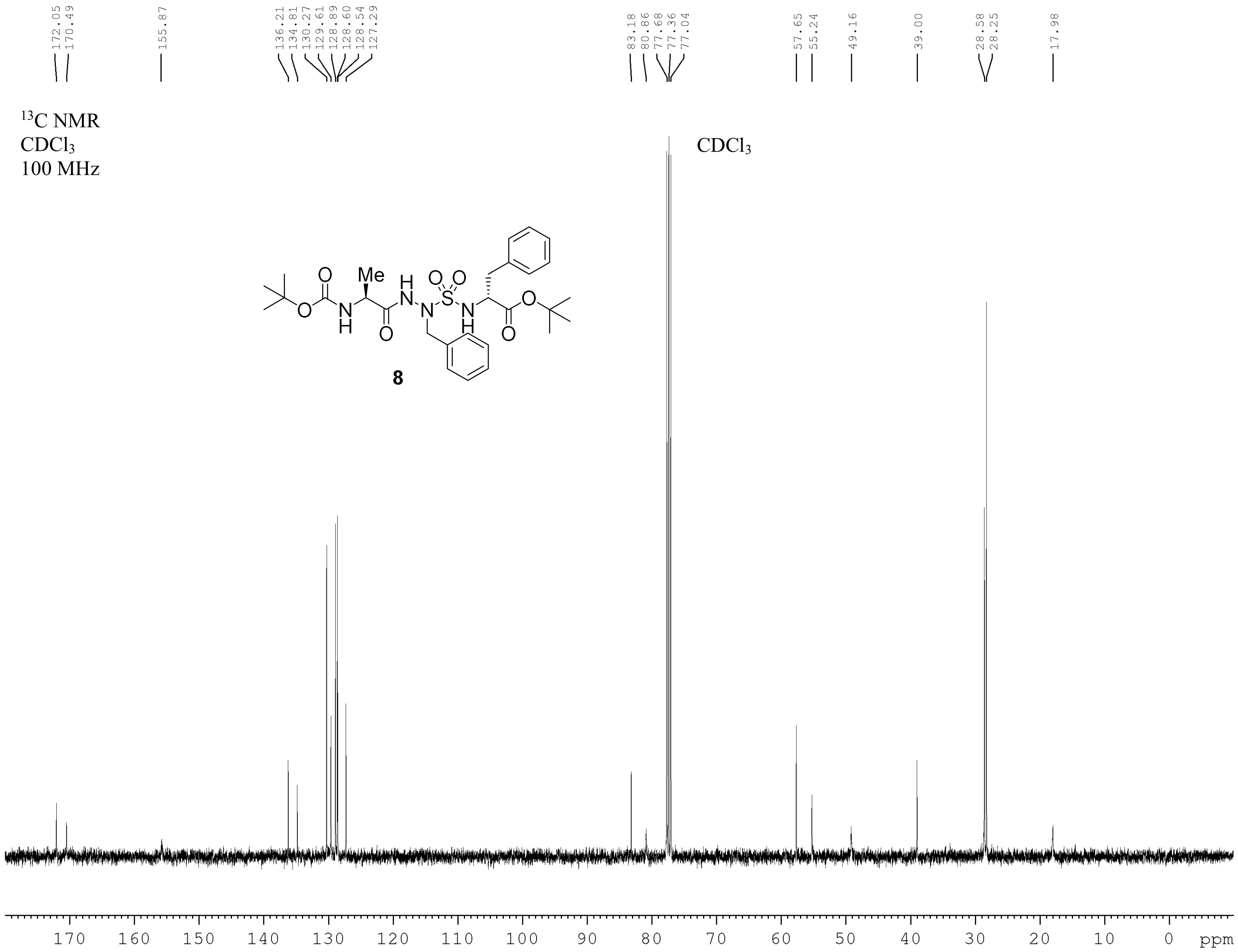

172.05
170.49
155.87
136.21
134.81
130.27
129.61
128.89
128.60
128.54
127.29
83.18
80.86
77.68
77.36
77.04
57.65
55.24
49.16
39.00
28.58
28.25
17.98
^{13}C NMR
$CDCl_3$
100 MHz
$CDCl_3$
Me
8
170 160 150 140 130 120 110 100 90 80 70 60 50 40 30 20 10 0 ppm

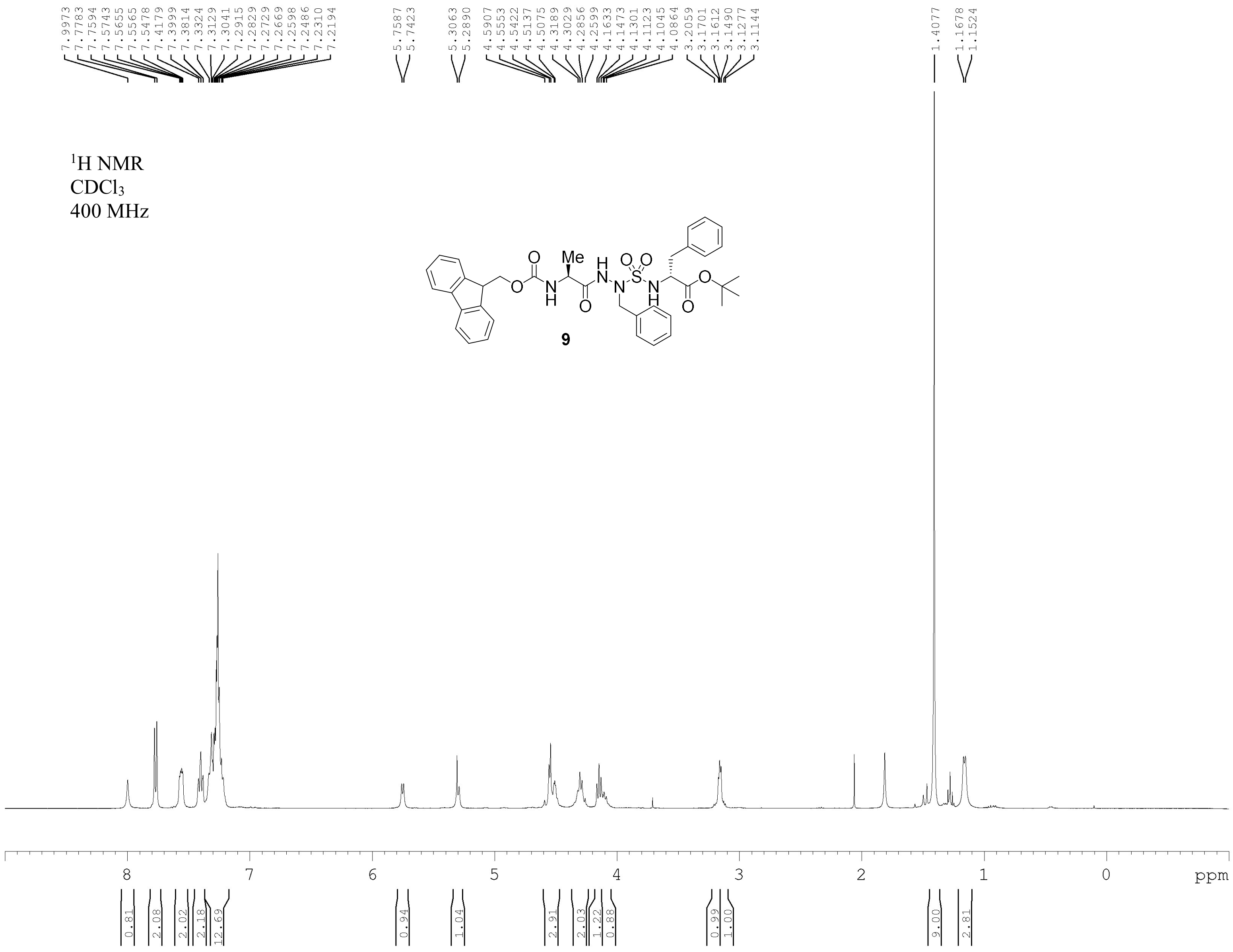
7.9973
7.7783
7.7594
7.5743
7.5655
7.5565
7.5478
7.4179
7.3999
7.3814
7.3324
7.3129
7.3041
7.2915
7.2829
7.2729
7.2669
7.2598
7.2486
7.2310
7.2194
5.7587
5.7423
5.3063
5.2890
4.5907
4.5553
4.5422
4.5137
4.5075
4.3189
4.3029
4.2856
4.2599
4.1633
4.1473
4.1301
4.1123
4.1045
4.0864
3.2059
3.1701
3.1612
3.1490
3.1277
3.1144
1.4077
1.1678
1.1524
^{1}H NMR
$CDCl_3$
400 MHz
Me
9
ppm
0.81
2.08
2.02
2.18
12.69
0.94
1.04
2.91
2.03
1.22
0.88
0.99
1.00
9.00
2.81

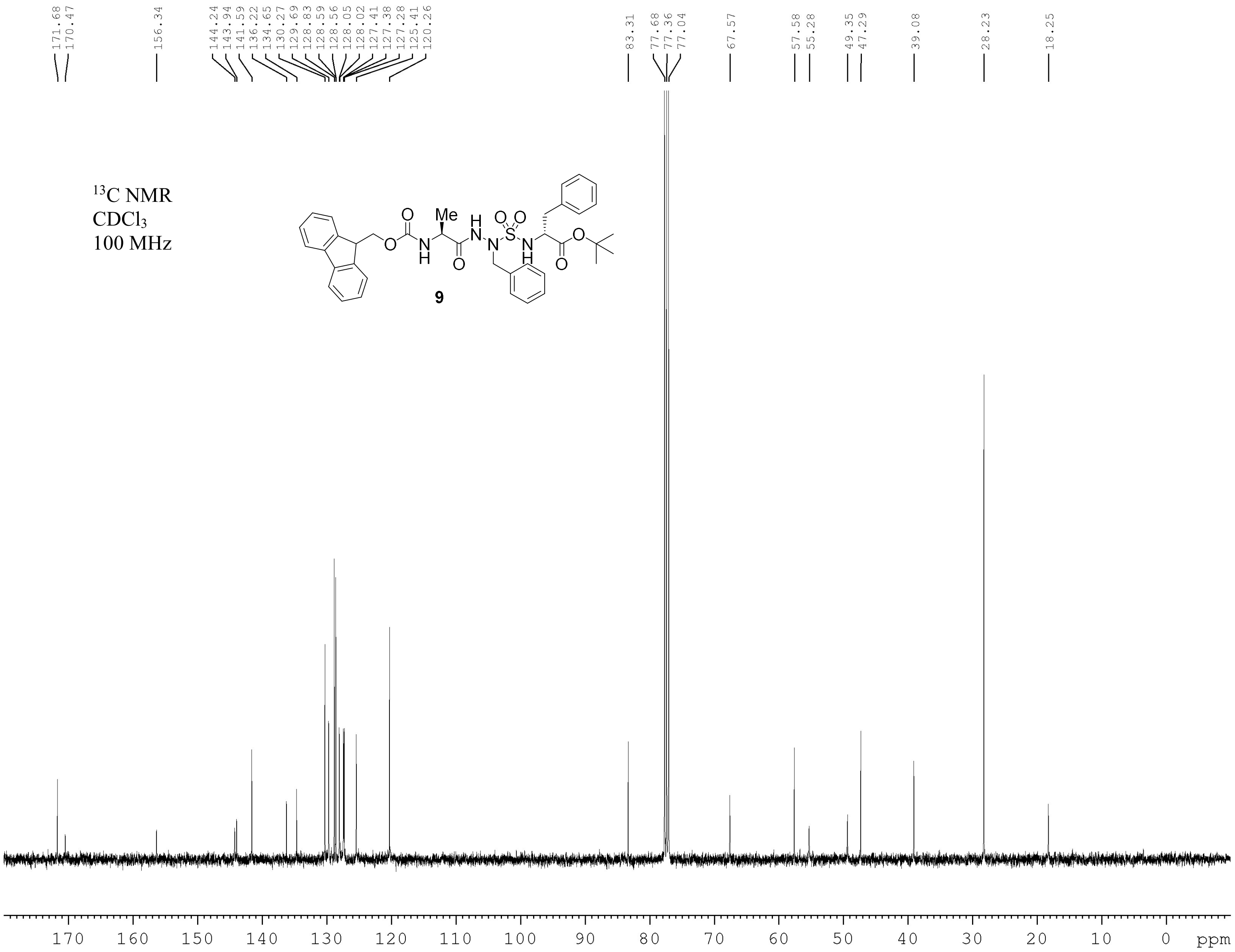
171.68
170.47
156.34
144.24
143.94
141.59
136.22
134.65
130.27
129.69
128.83
128.59
128.56
128.05
128.02
127.41
127.38
127.28
125.41
120.26
83.31
77.68
77.36
77.04
67.57
57.58
55.28
49.35
47.29
39.08
28.23
18.25
^{13}C NMR
$CDCl_3$
100 MHz
9
170 160 150 140 130 120 110 100 90 80 70 60 50 40 30 20 10 0 ppm

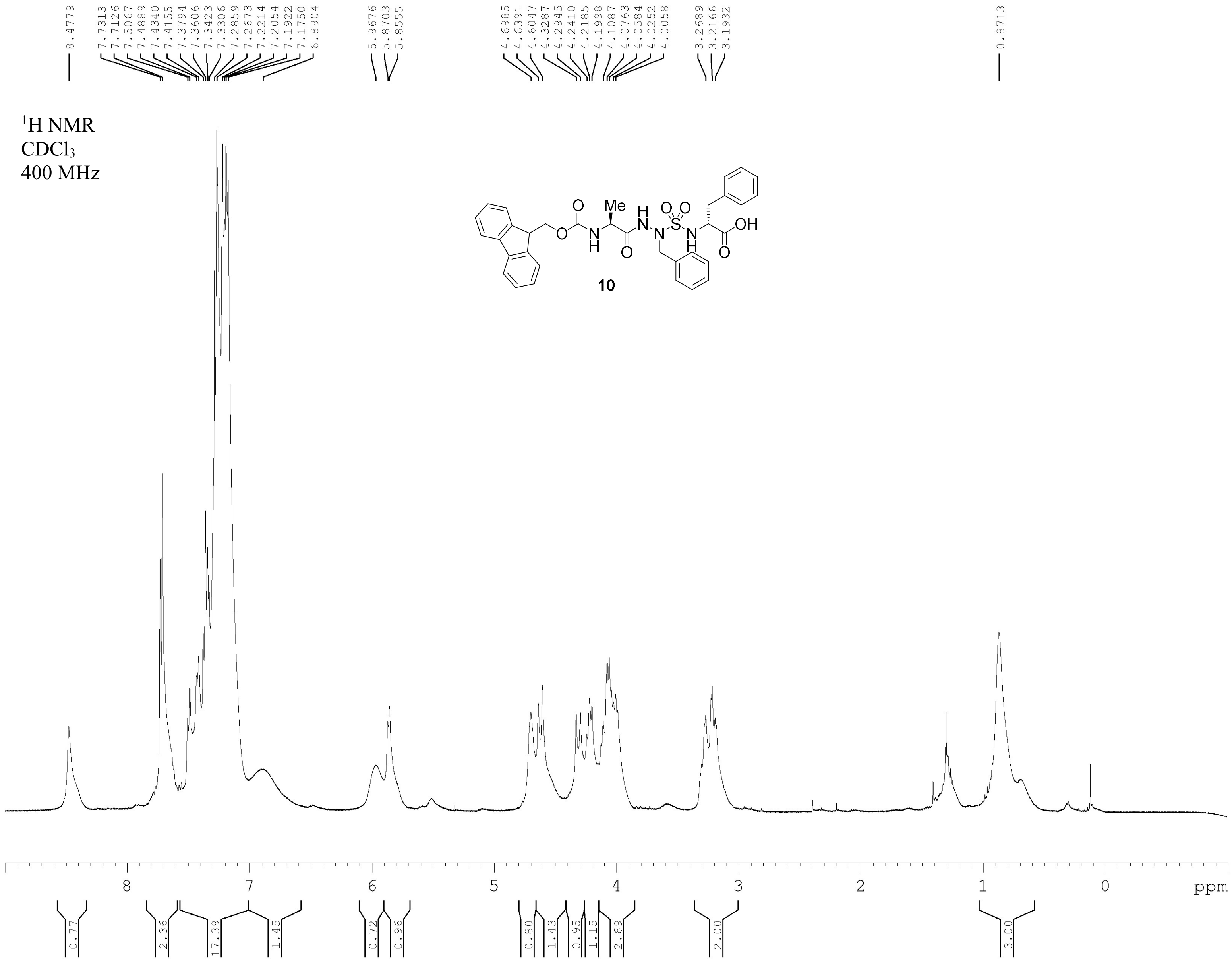
8.4779
7.7313 7.7126 7.5067 7.4889 7.4340 7.4155 7.3794 7.3606 7.3423 7.3306 7.2859 7.2673 7.2214 7.2054 7.1922 7.1750 6.8904
5.9676 5.8703 5.8555
4.6985 4.6391 4.6047 4.3287 4.2945 4.2410 4.2185 4.1998 4.1087 4.0763 4.0584 4.0252 4.0058
3.2689 3.2166 3.1932
0.8713
1H NMR
CDCl3
400 MHz
10
ppm
0.77 2.36 17.39 1.45 0.72 0.96 0.80 1.43 0.95 1.15 2.69 2.00 3.00

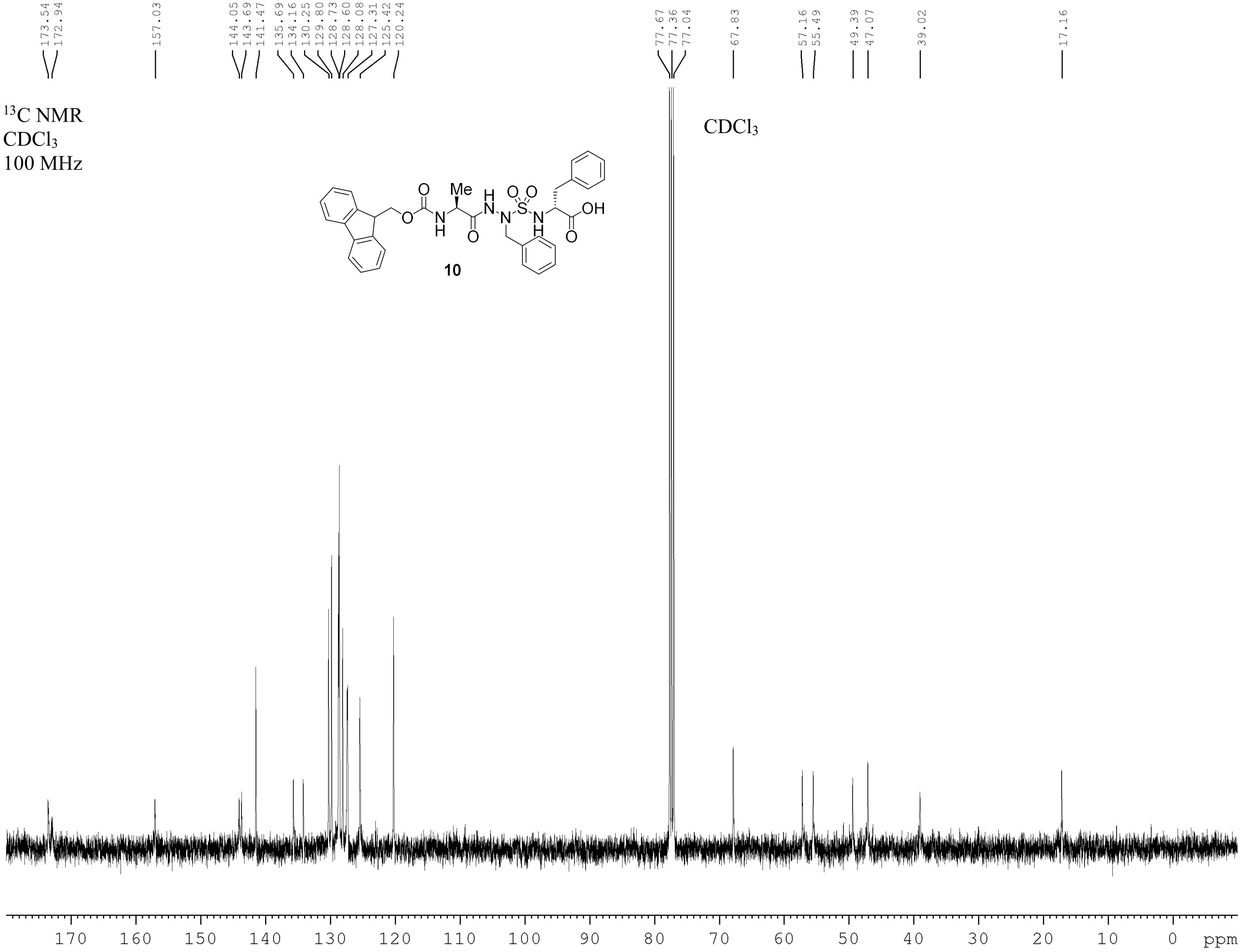
173.54
172.94
157.03
144.05
143.69
141.47
135.69
134.16
130.25
129.80
128.73
128.60
128.08
127.31
125.42
120.24
77.67
77.36
77.04
67.83
57.16
55.49
49.39
47.07
39.02
17.16
13C NMR
CDCl3
100 MHz
CDCl3
Me
O
H
N
O
OH
10
170 160 150 140 130 120 110 100 90 80 70 60 50 40 30 20 10 0 ppm

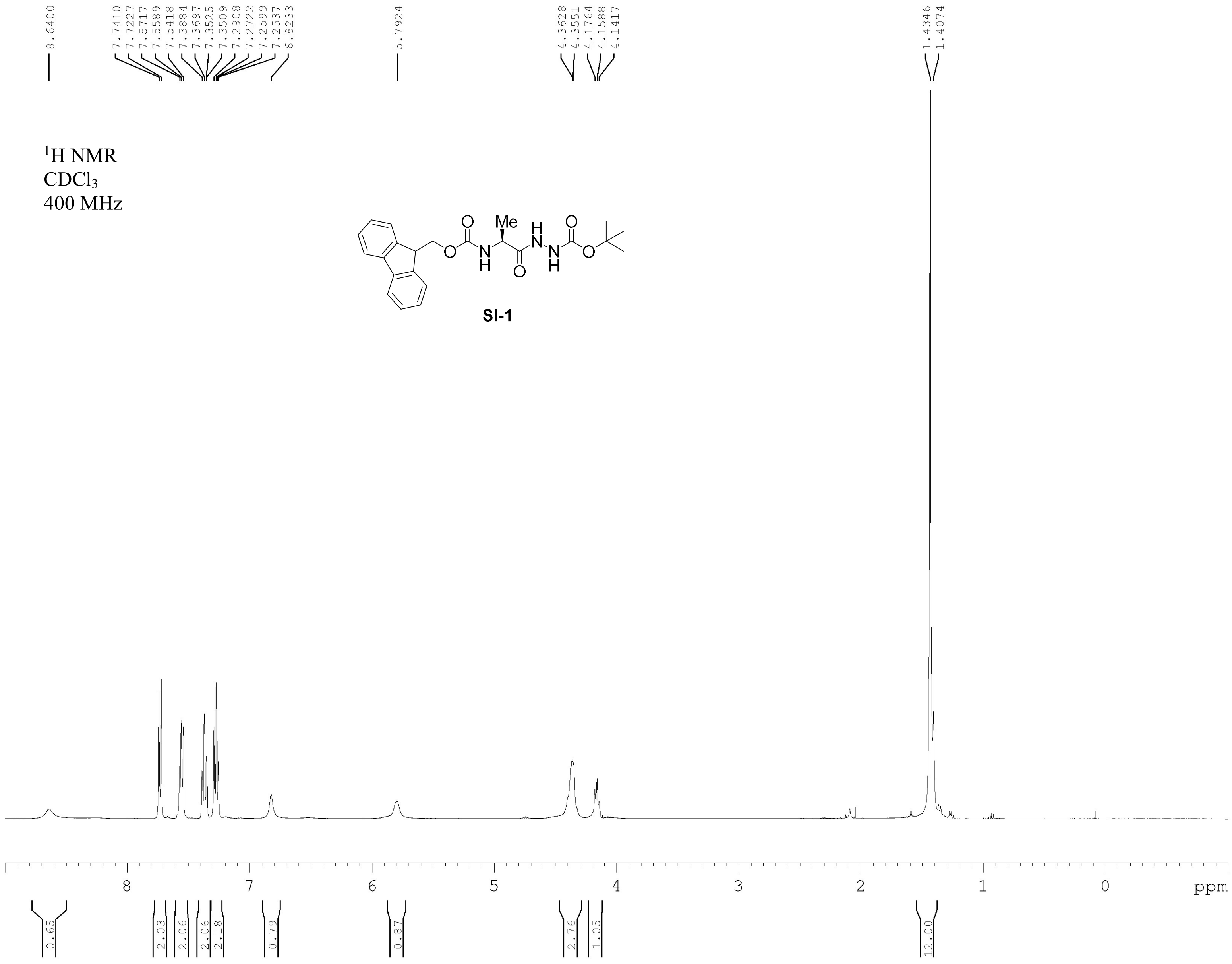

8.6400
7.7410
7.7227
7.5717
7.5589
7.5418
7.3884
7.3697
7.3525
7.3509
7.2908
7.2722
7.2599
7.2537
6.8233
5.7924
4.3628
4.3551
4.1764
4.1588
4.1417
1.4346
1.4074
^{1}H NMR
$CDCl_3$
400 MHz
Me
SI-1
8
7
6
5
4
3
2
1
0
ppm
0.65
2.03
2.06
2.06
2.18
0.79
0.87
2.76
1.05
12.00

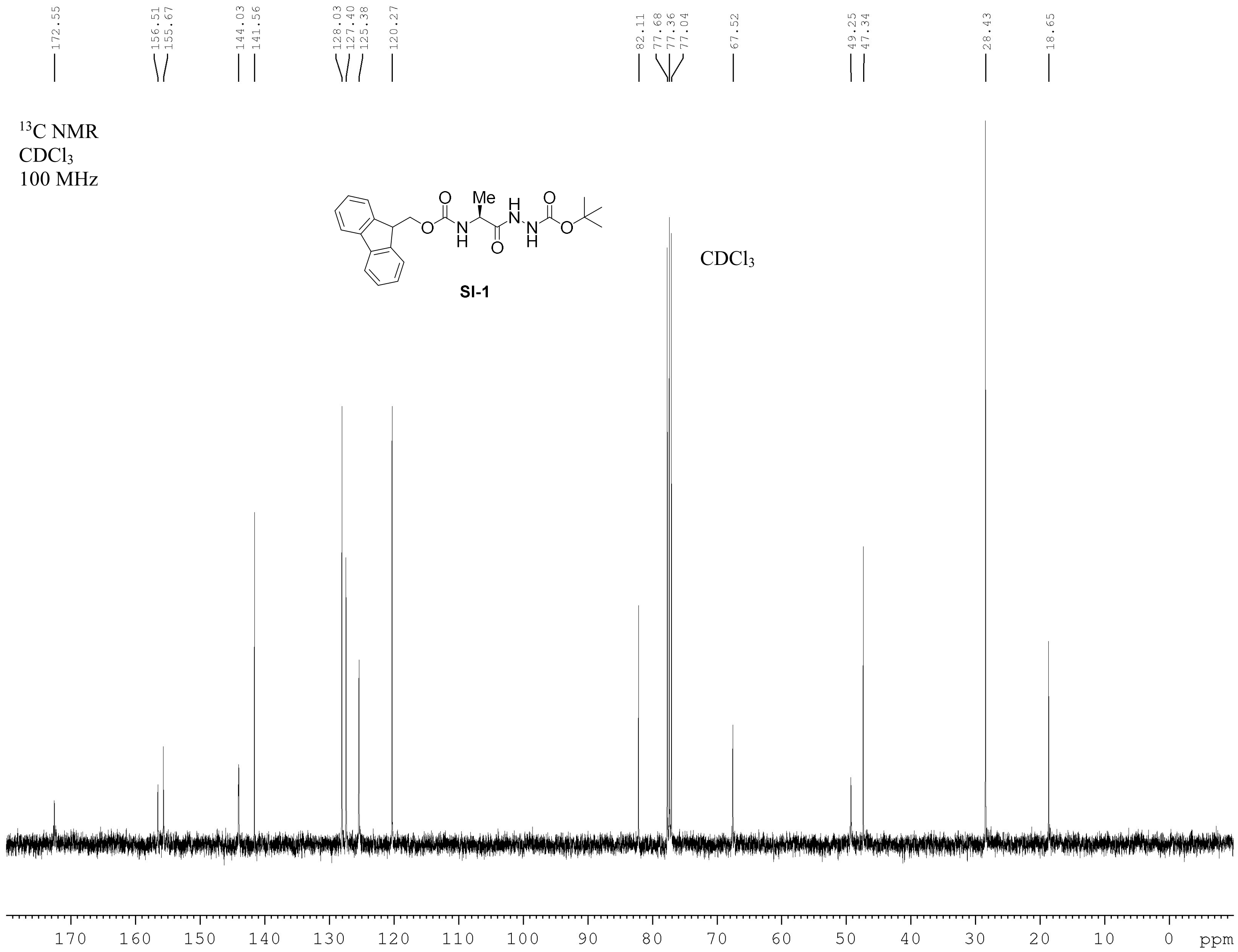
172.55
156.51
155.67
144.03
141.56
128.03
127.40
125.38
120.27
82.11
77.68
77.36
77.04
67.52
49.25
47.34
28.43
18.65
13C NMR
CDCl3
100 MHz
Me
SI-1
CDCl3
170 160 150 140 130 120 110 100 90 80 70 60 50 40 30 20 10 0 ppm

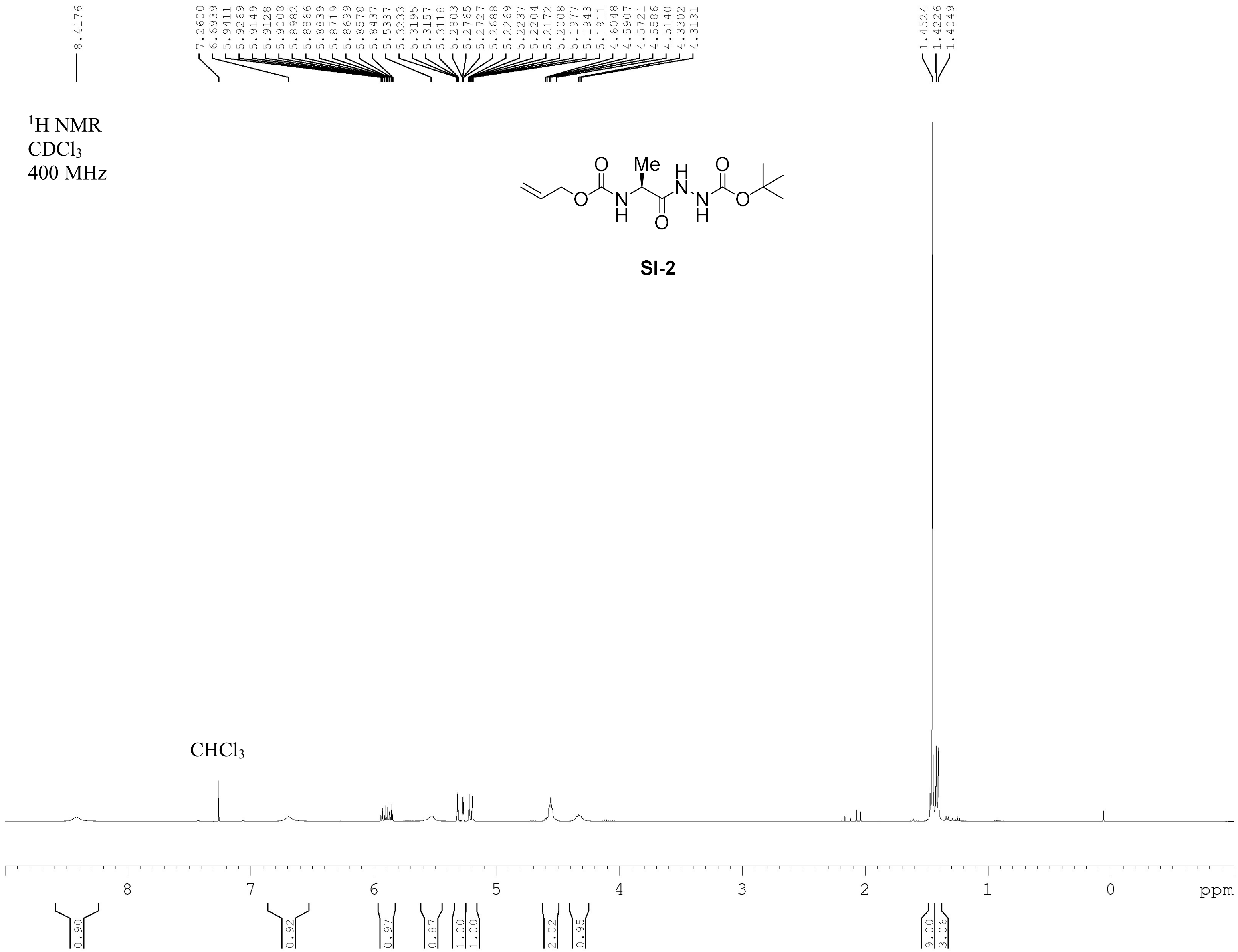

8.4176
7.2600
6.6939
5.9411
5.9269
5.9149
5.9128
5.9008
5.8982
5.8866
5.8839
5.8719
5.8699
5.8578
5.8437
5.5337
5.3233
5.3195
5.3157
5.3118
5.2803
5.2765
5.2727
5.2688
5.2269
5.2237
5.2204
5.2172
5.2008
5.1977
5.1943
5.1911
4.6048
4.5907
4.5721
4.5586
4.5140
4.3302
4.3131
1.4524
1.4226
1.4049
^{1}H NMR
$CDCl_3$
400 MHz
Me
H
N
O
SI-2
$CHCl_3$
8
7
6
5
4
3
2
1
0
ppm
0.90
0.92
0.97
0.87
1.00
1.00
2.02
0.95
9.00
3.06

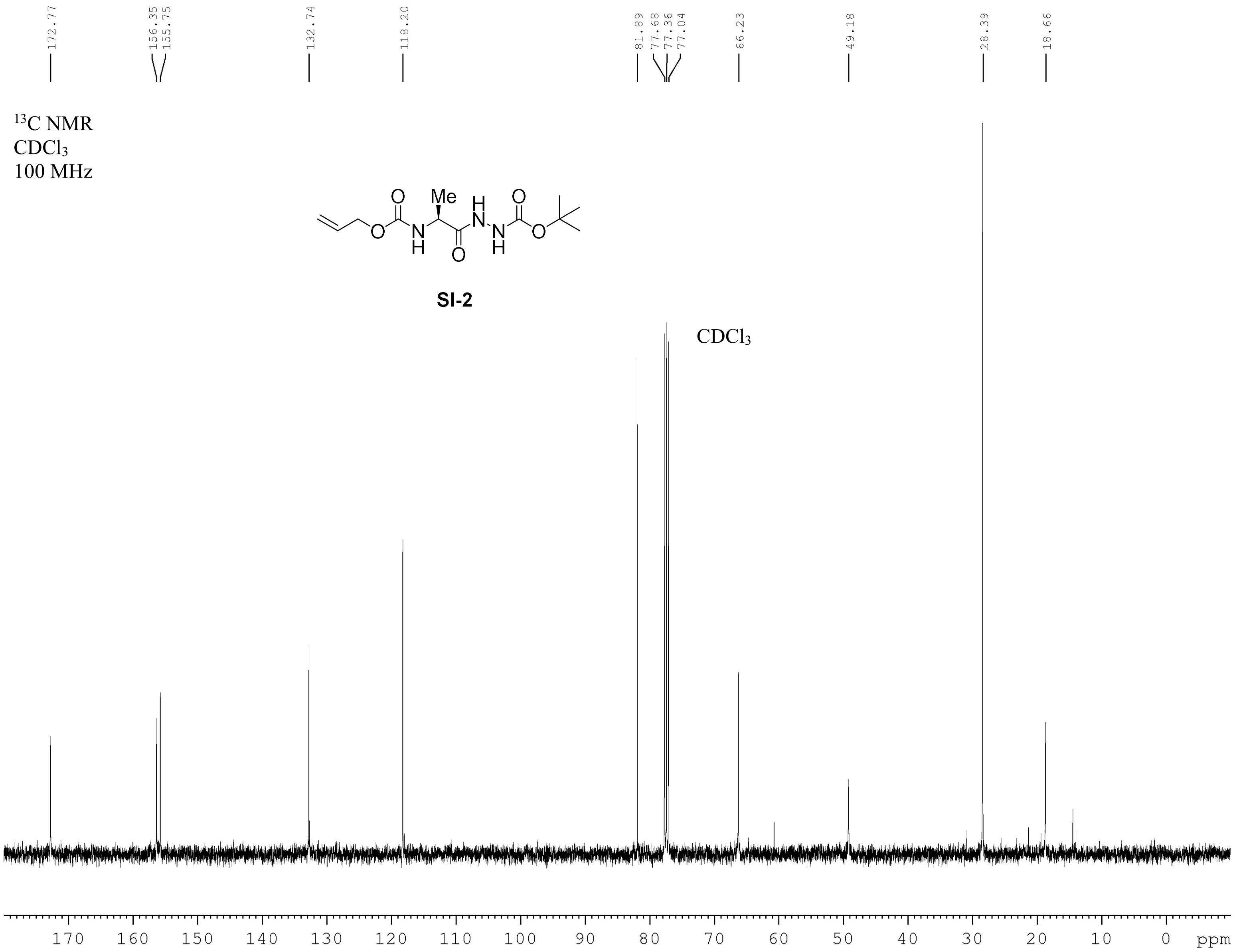
172.77
156.35
155.75
132.74
118.20
81.89
77.68
77.36
77.04
66.23
49.18
28.39
18.66
^{13}C NMR
$CDCl_3$
100 MHz
Me
O
N
H
SI-2
$CDCl_3$
170 160 150 140 130 120 110 100 90 80 70 60 50 40 30 20 10 0 ppm

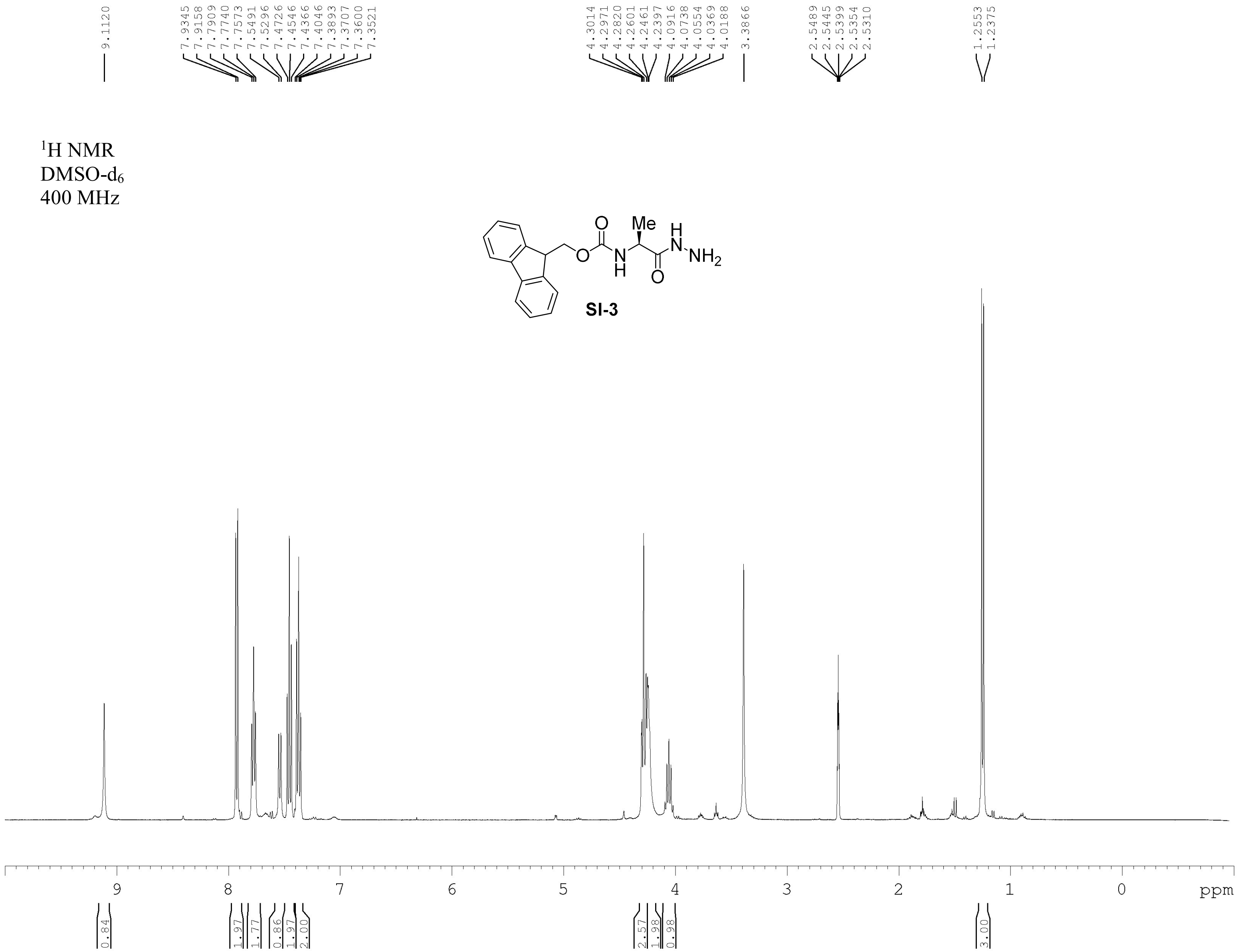

9.1120
7.9345
7.9158
7.7909
7.7740
7.7573
7.5491
7.5296
7.4726
7.4546
7.4366
7.4046
7.3893
7.3707
7.3600
7.3521
4.3014
4.2971
4.2820
4.2601
4.2461
4.2397
4.0916
4.0738
4.0554
4.0369
4.0188
3.3866
2.5489
2.5445
2.5399
2.5354
2.5310
1.2553
1.2375
^{1}H NMR
DMSO-d$_6$
400 MHz
Me
O
H
N
NH$_2$
SI-3
9
8
7
6
5
4
3
2
1
0
ppm
0.84
1.97
1.77
0.86
1.97
2.00
2.57
1.98
0.98
3.00

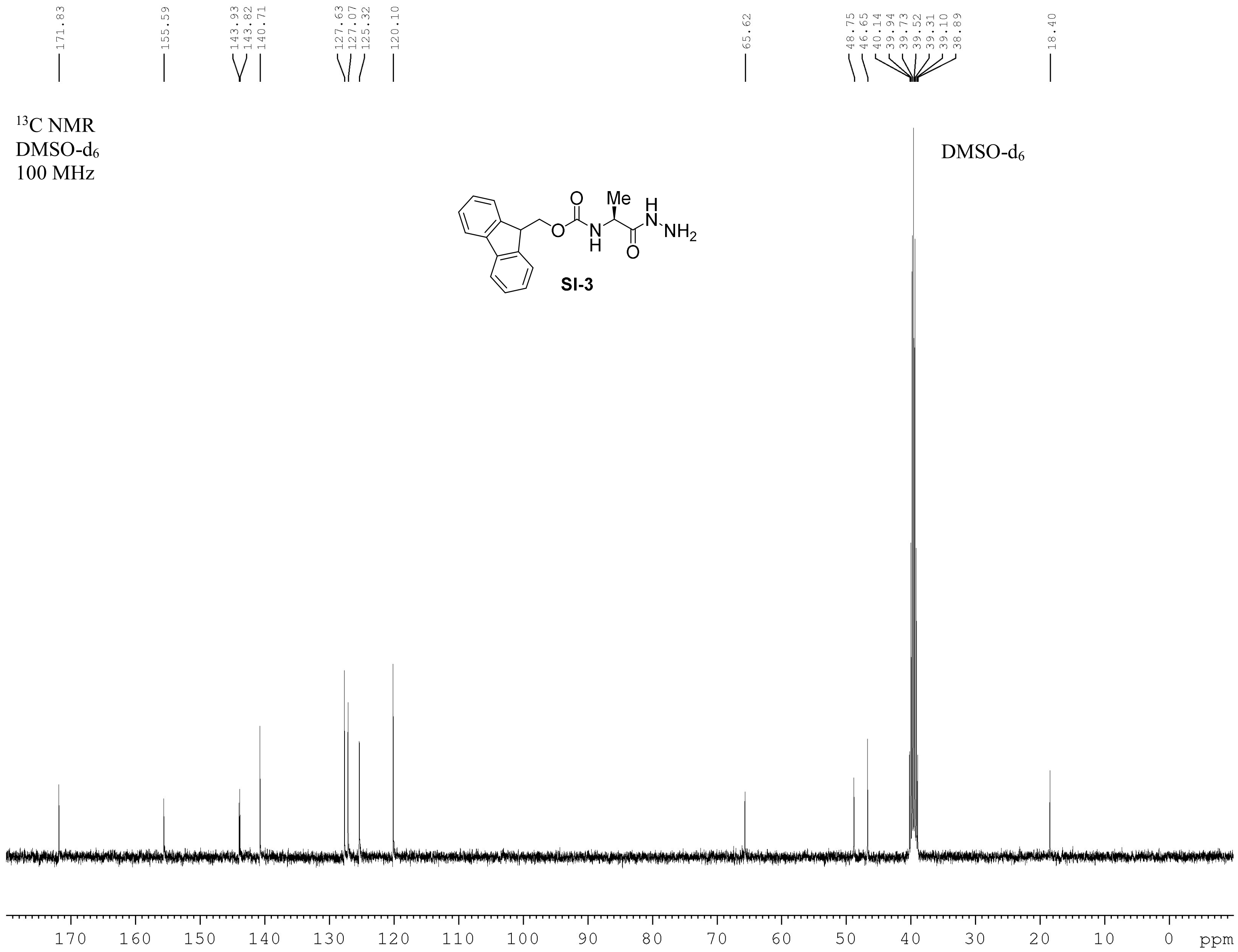
171.83
155.59
143.93
143.82
140.71
127.63
127.07
125.32
120.10
65.62
48.75
46.65
40.14
39.94
39.73
39.52
39.31
39.10
38.89
18.40
13C NMR
DMSO-d6
100 MHz
DMSO-d6
SI-3
170 160 150 140 130 120 110 100 90 80 70 60 50 40 30 20 10 0 ppm

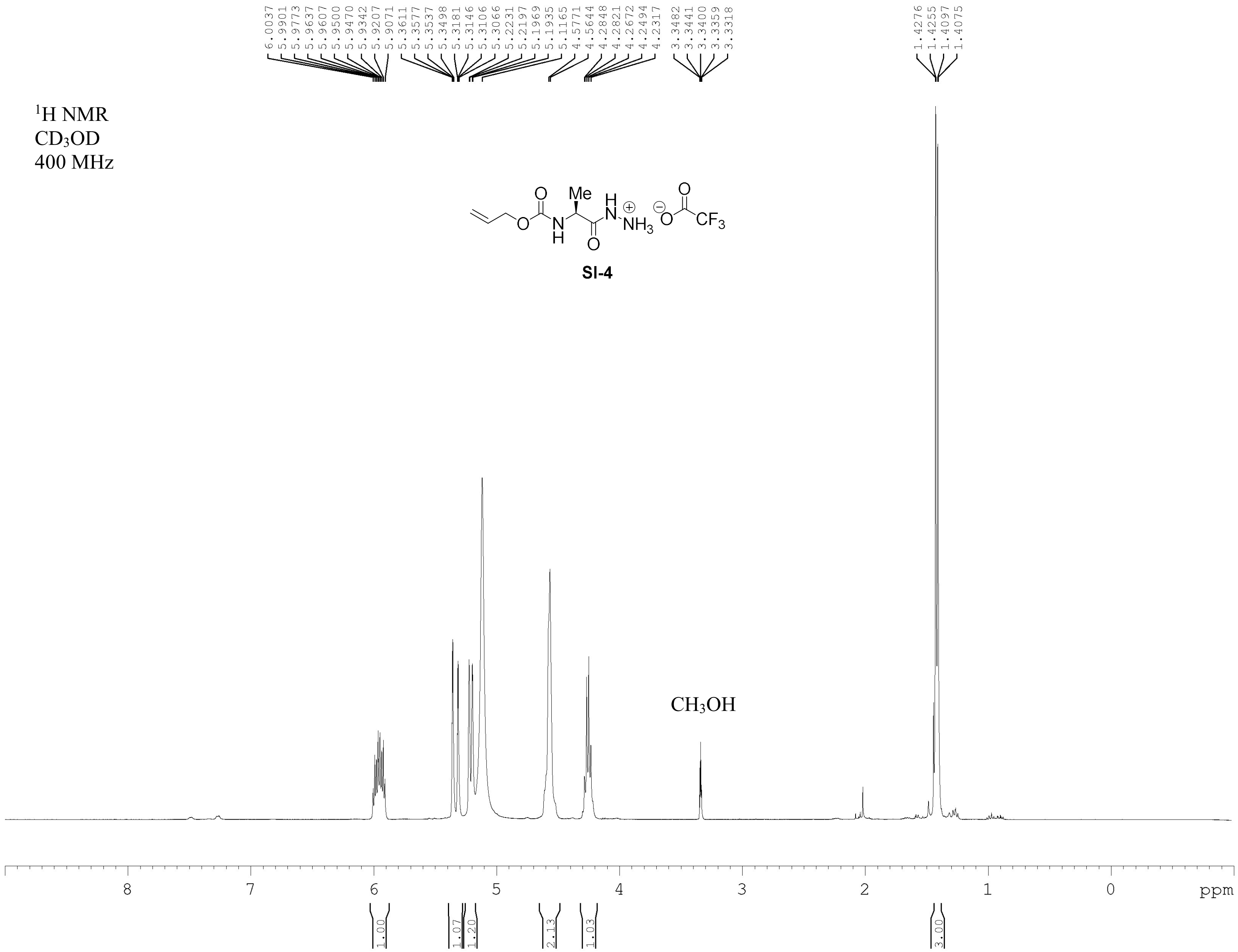

1H NMR
CD3OD
400 MHz
SI-4
CH3OH
6.0037
5.9901
5.9773
5.9637
5.9607
5.9500
5.9470
5.9342
5.9207
5.9071
5.3611
5.3577
5.3537
5.3498
5.3181
5.3146
5.3106
5.3066
5.2231
5.2197
5.1969
5.1935
5.1165
4.5771
4.5644
4.2848
4.2821
4.2672
4.2494
4.2317
3.3482
3.3441
3.3400
3.3359
3.3318
1.4276
1.4255
1.4097
1.4075
1.00
1.07
1.20
2.13
1.03
3.00
ppm

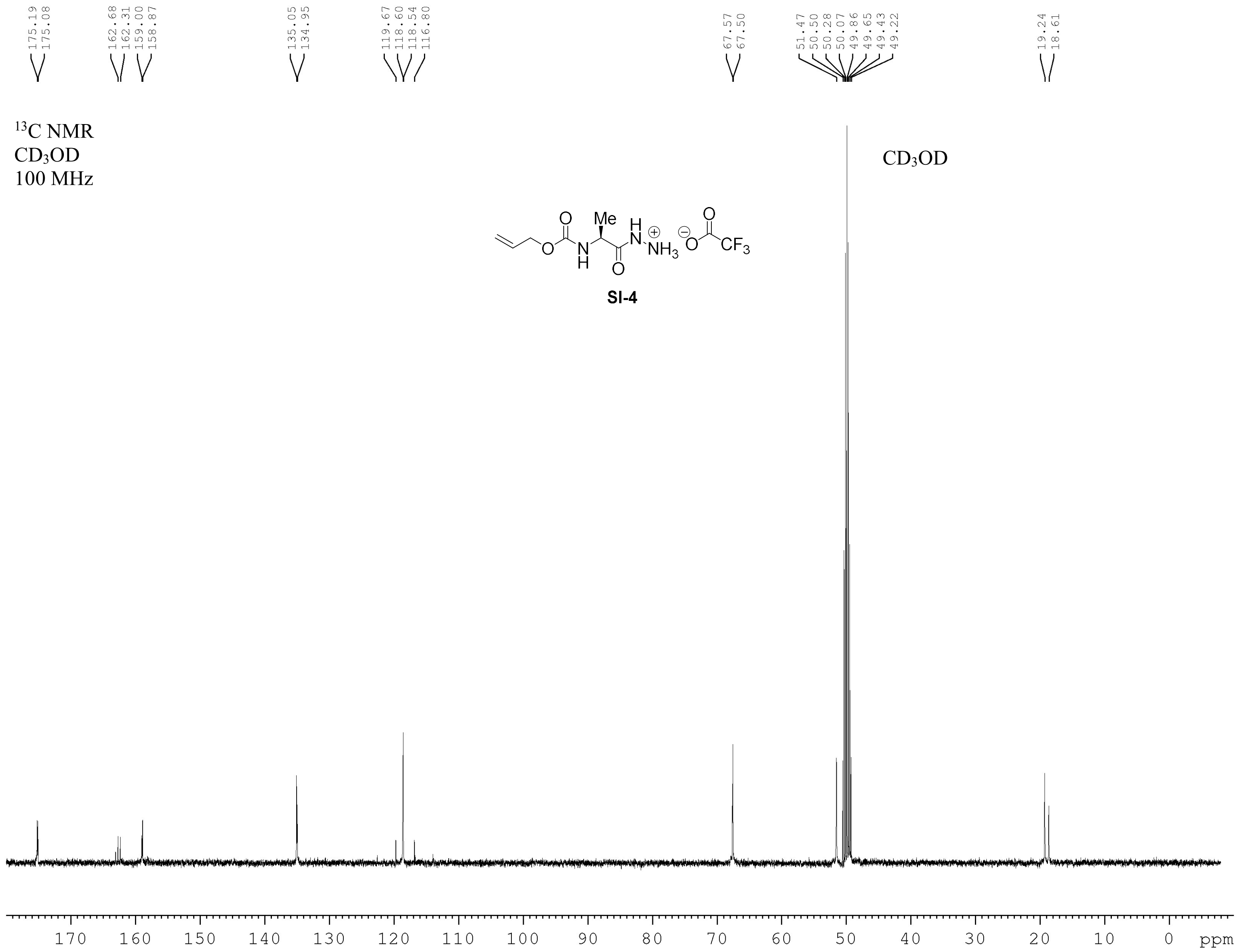
175.19
175.08
162.68
162.31
159.00
158.87
135.05
134.95
119.67
118.60
118.54
116.80
67.57
67.50
51.47
50.50
50.28
50.07
49.86
49.65
49.43
49.22
19.24
18.61
13C NMR
CD3OD
100 MHz
CD3OD
SI-4
170
160
150
140
130
120
110
100
90
80
70
60
50
40
30
20
10
0
ppm

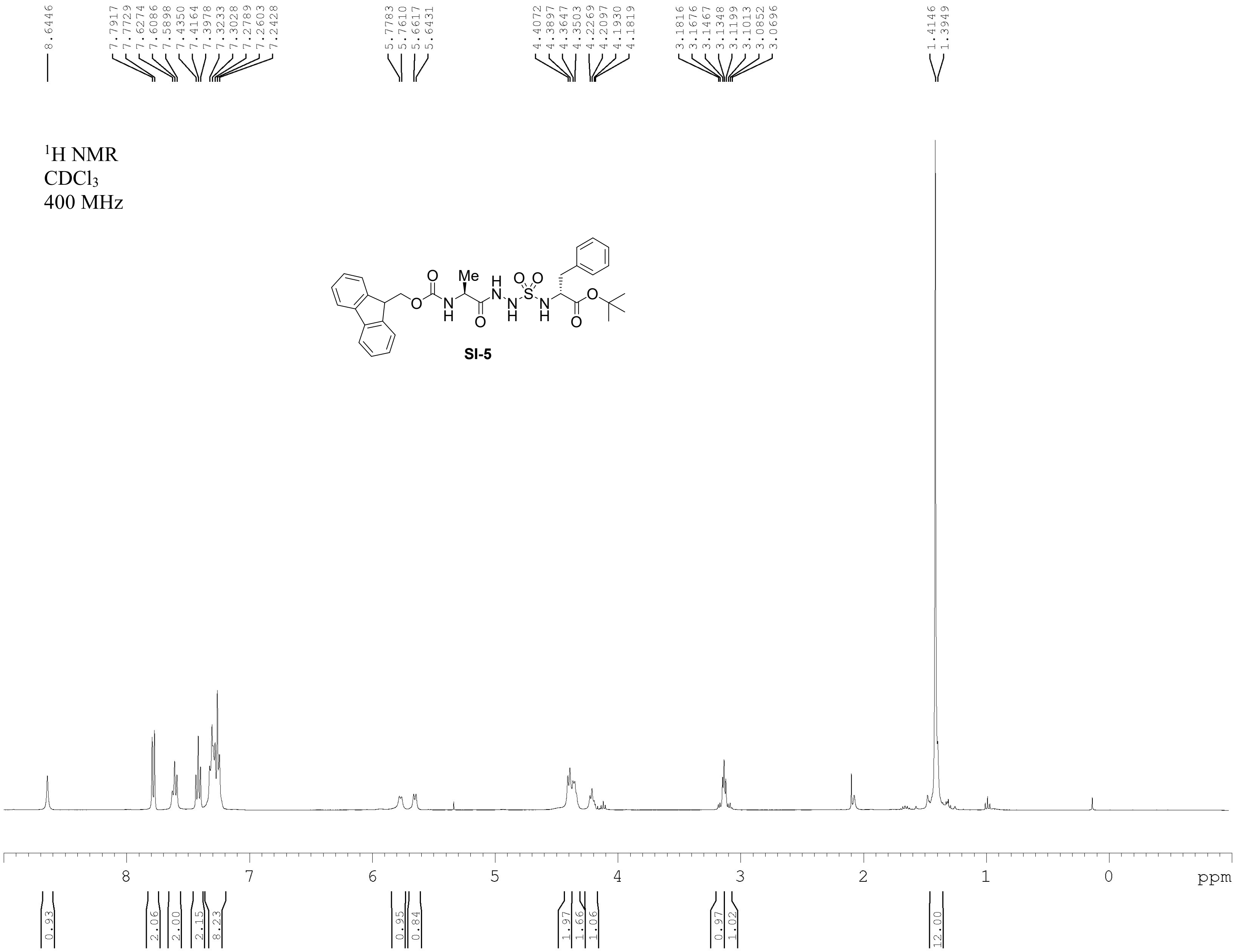

^{1}H NMR
$CDCl_3$
400 MHz

SI-5

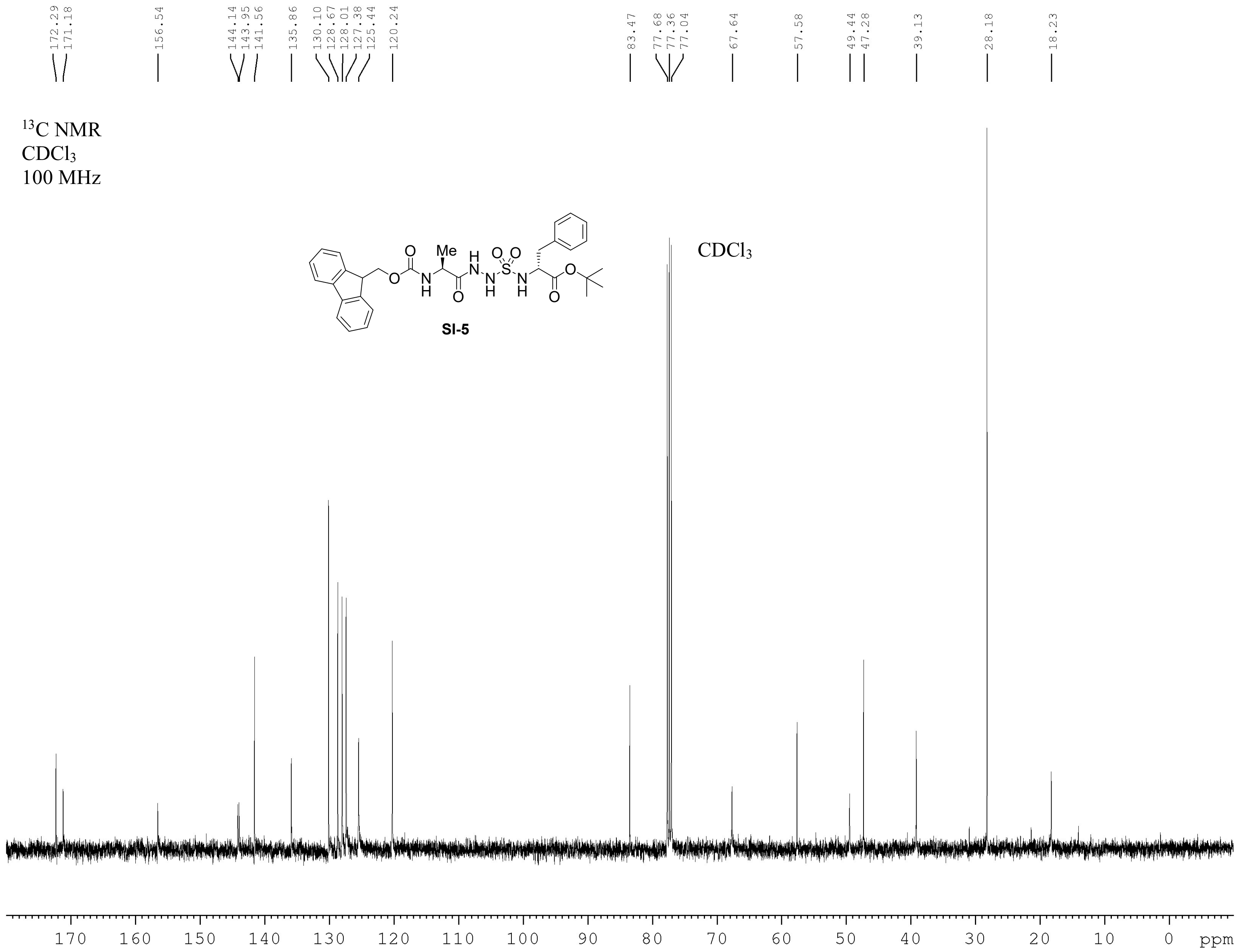
172.29
171.18
156.54
144.14
143.95
141.56
135.86
130.10
128.67
128.01
127.38
125.44
120.24
83.47
77.68
77.36
77.04
67.64
57.58
49.44
47.28
39.13
28.18
18.23
13C NMR
CDCl3
100 MHz
CDCl3
SI-5
170 160 150 140 130 120 110 100 90 80 70 60 50 40 30 20 10 0 ppm

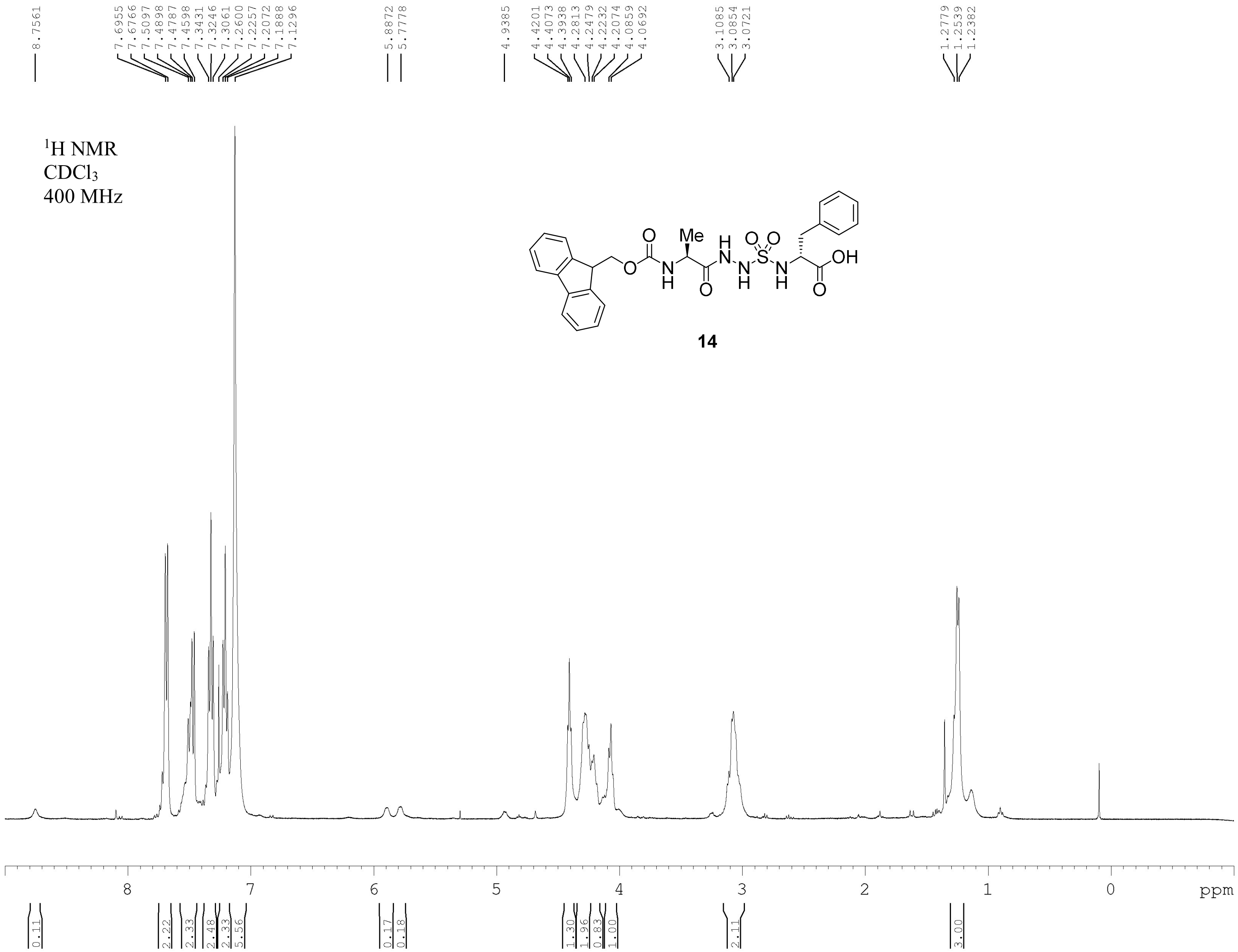

8.7561
7.6955
7.6766
7.5097
7.4898
7.4787
7.4598
7.3431
7.3246
7.3061
7.2600
7.2257
7.2072
7.1888
7.1296
5.8872
5.7778
4.9385
4.4201
4.4073
4.3938
4.2813
4.2479
4.2232
4.2074
4.0859
4.0692
3.1085
3.0854
3.0721
1.2779
1.2539
1.2382
^{1}H NMR
$CDCl_3$
400 MHz
O
Me
H
N
H
O
O
S
N
H
OH
O
14
8
7
6
5
4
3
2
1
0
ppm
0.11
2.22
2.33
2.48
2.33
5.56
0.17
0.18
1.30
1.96
0.83
1.00
2.11
3.00

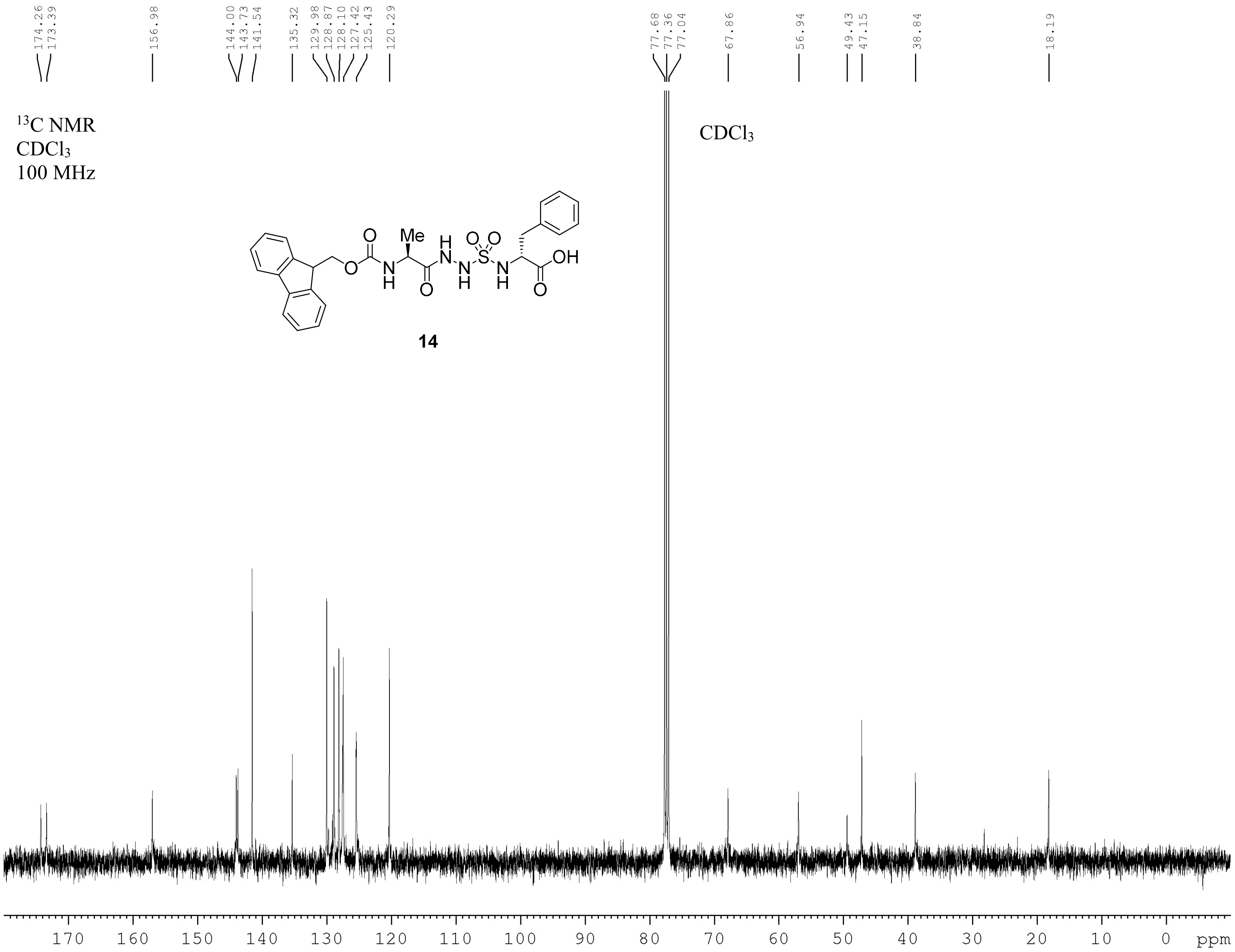
174.26
173.39
156.98
144.00
143.73
141.54
135.32
129.98
128.87
128.10
127.42
125.43
120.29
77.68
77.36
77.04
67.86
56.94
49.43
47.15
38.84
18.19
13C NMR
CDCl3
100 MHz
CDCl3
Me
OH
14
170 160 150 140 130 120 110 100 90 80 70 60 50 40 30 20 10 0 ppm

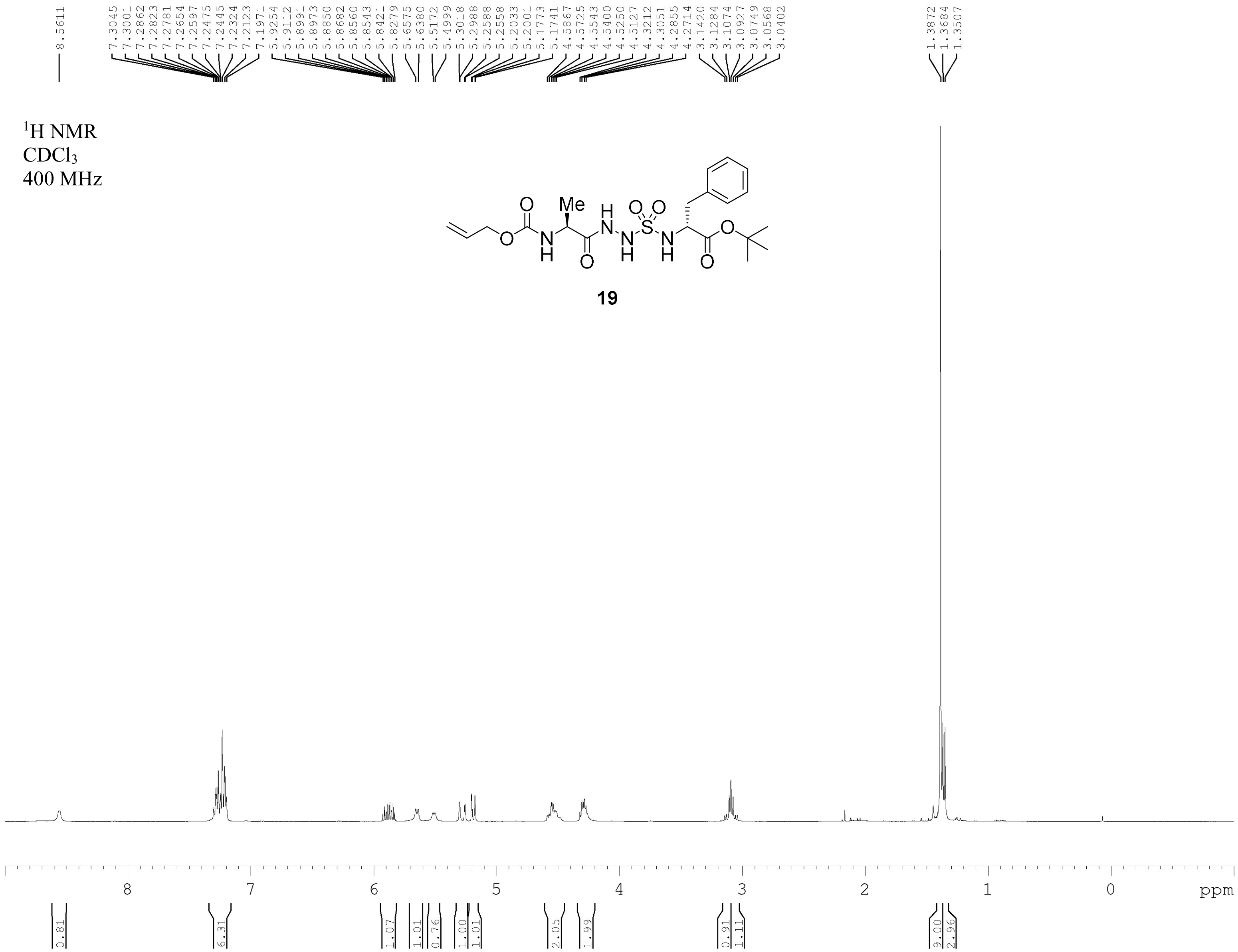
^{1}H NMR
$CDCl_3$
400 MHz
Me
19
8 7 6 5 4 3 2 1 0 ppm
0.81 6.31 1.07 1.01 0.76 1.00 1.01 2.05 1.99 0.91 1.11 9.00 2.96

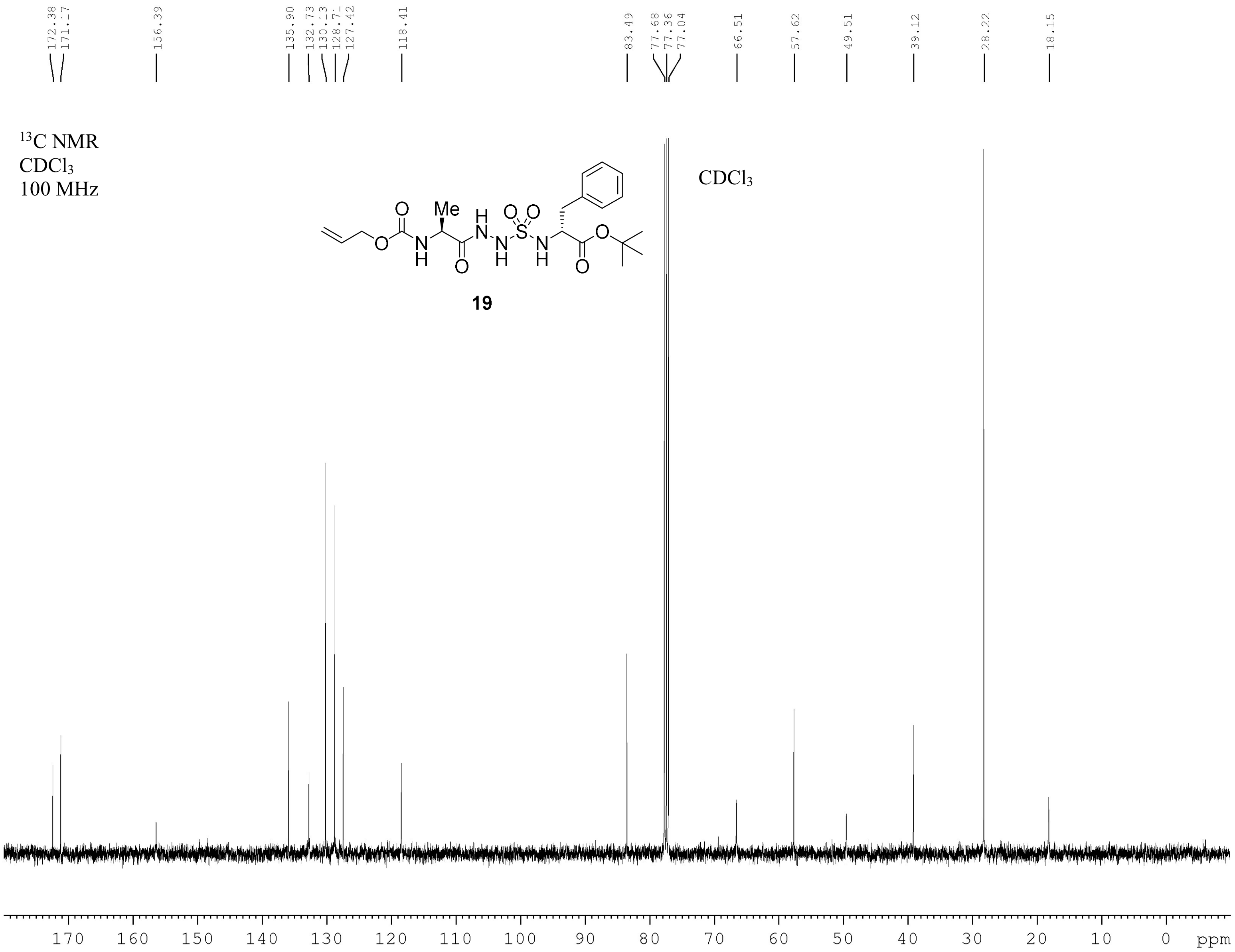
172.38
171.17
156.39
135.90
132.73
130.13
128.71
127.42
118.41
83.49
77.68
77.36
77.04
66.51
57.62
49.51
39.12
28.22
18.15
^{13}C NMR
$CDCl_3$
100 MHz
$CDCl_3$
19
170
160
150
140
130
120
110
100
90
80
70
60
50
40
30
20
10
0
ppm

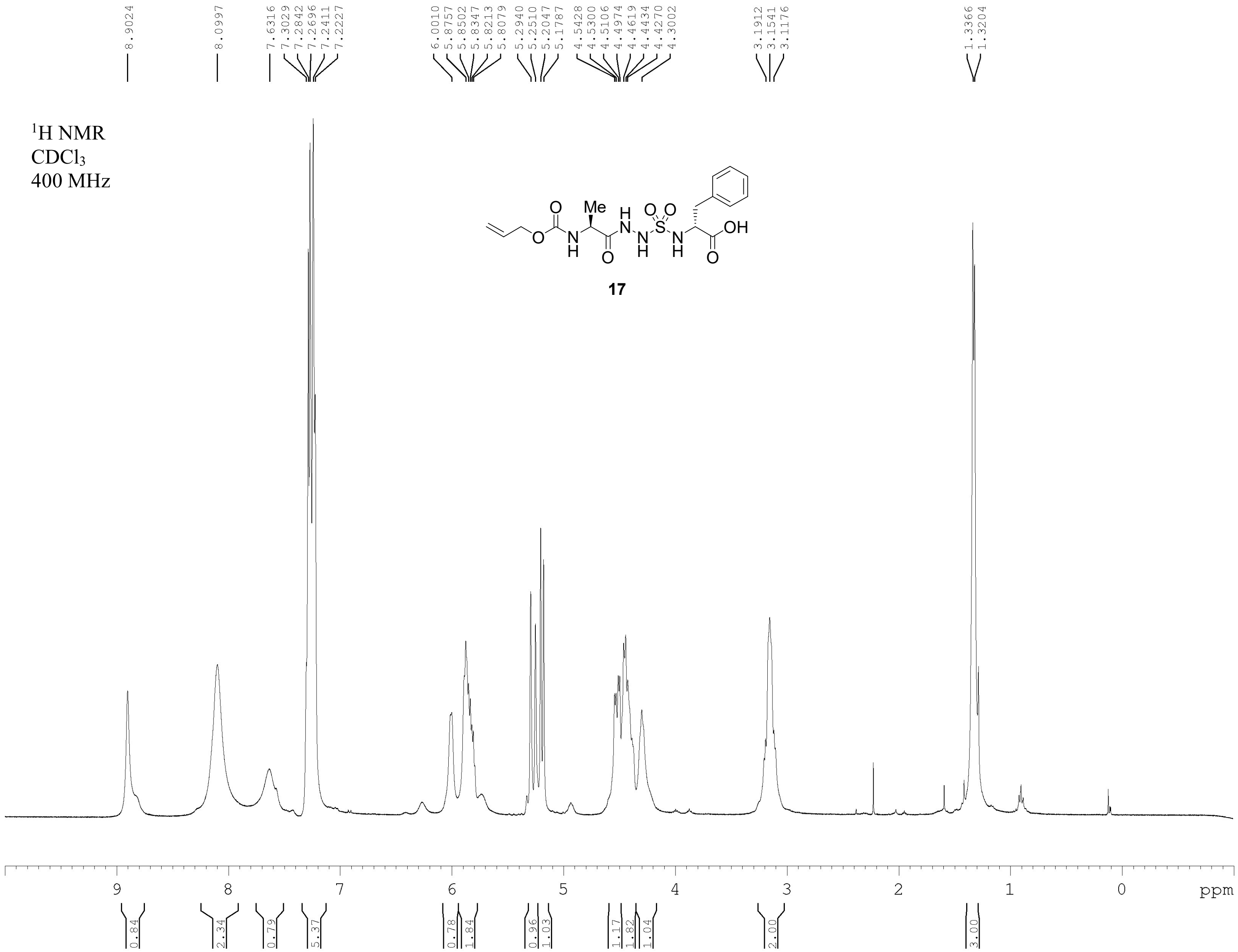

^{1}H NMR
$CDCl_3$
400 MHz
17
8.9024
8.0997
7.6316
7.3029
7.2842
7.2696
7.2411
7.2227
6.0010
5.8757
5.8502
5.8347
5.8213
5.8079
5.2940
5.2510
5.2047
5.1787
4.5428
4.5300
4.5106
4.4974
4.4619
4.4434
4.4270
4.3002
3.1912
3.1541
3.1176
1.3366
1.3204
ppm
0.84
2.34
0.79
5.37
0.78
1.84
0.96
1.03
1.17
1.82
1.04
2.00
3.00

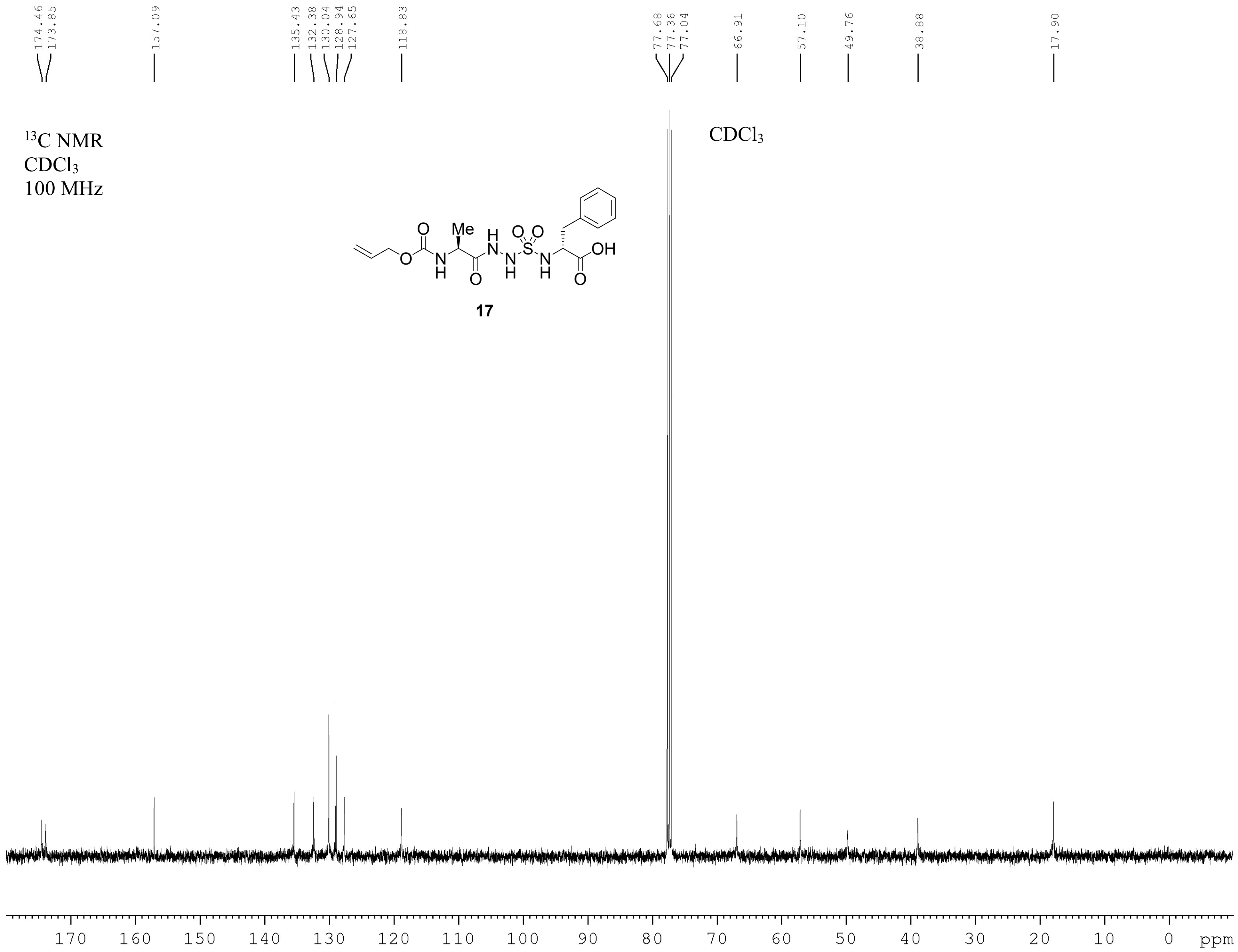
174.46
173.85
157.09
135.43
132.38
130.04
128.94
127.65
118.83
77.68
77.36
77.04
66.91
57.10
49.76
38.88
17.90
^{13}C NMR
$CDCl_3$
100 MHz
$CDCl_3$
Me
O
O
OH
17
170 160 150 140 130 120 110 100 90 80 70 60 50 40 30 20 10 0 ppm

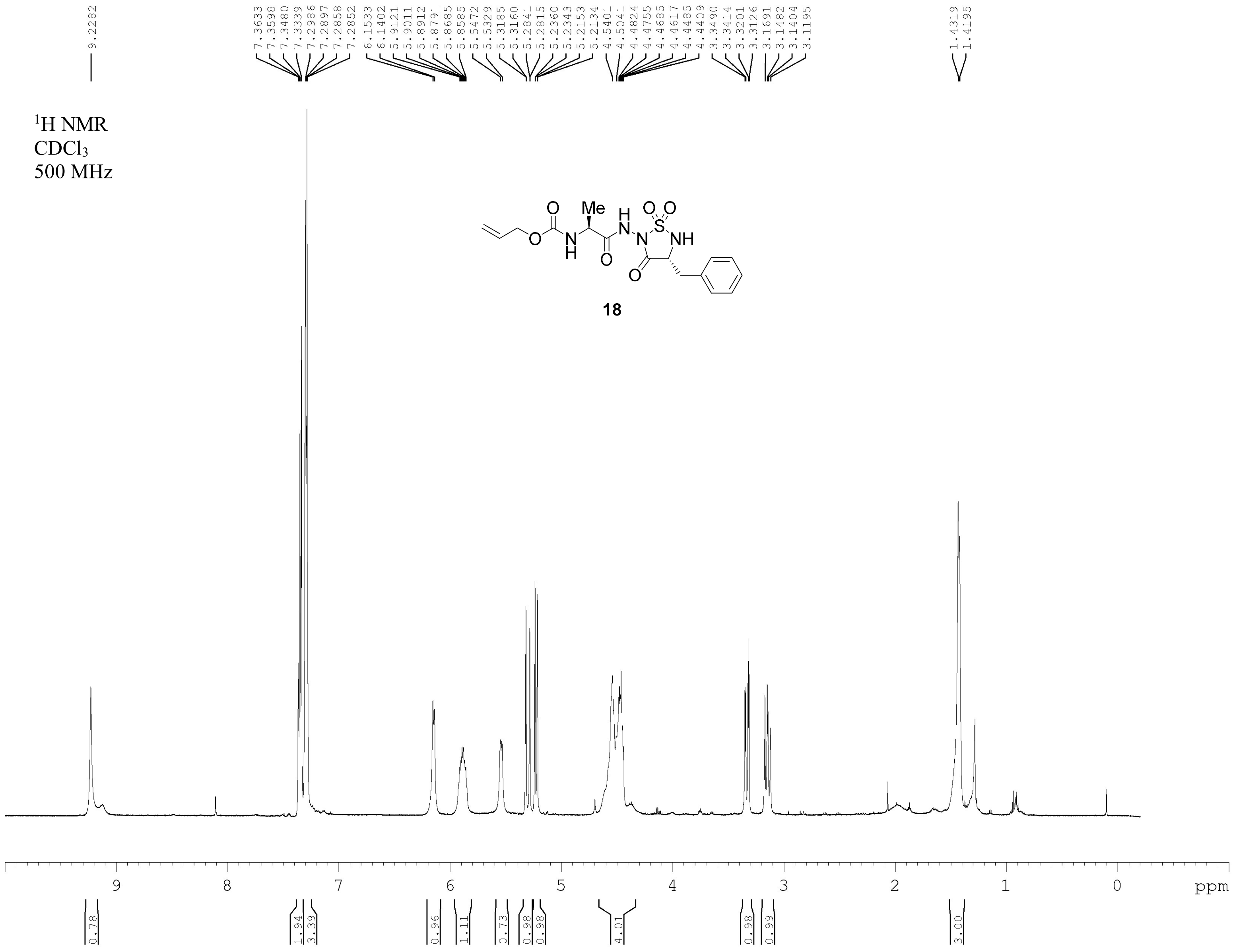
^{1}H NMR
$CDCl_3$
500 MHz
Me
18
ppm

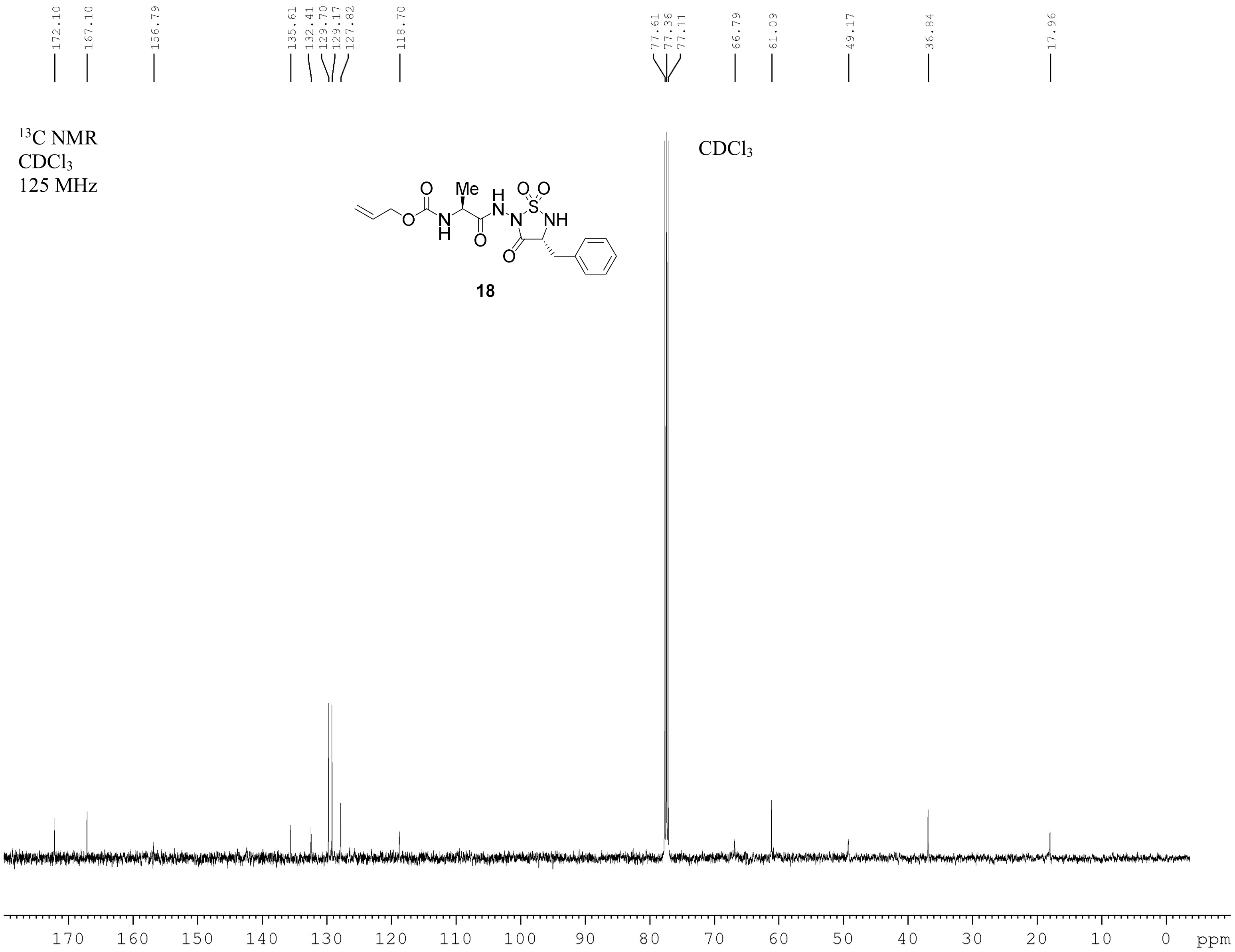
172.10
167.10
156.79
135.61
132.41
129.70
129.17
127.82
118.70
77.61
77.36
77.11
66.79
61.09
49.17
36.84
17.96
^{13}C NMR
$CDCl_3$
125 MHz
$CDCl_3$
Me
18
170 160 150 140 130 120 110 100 90 80 70 60 50 40 30 20 10 0 ppm

COSY NMR
$CDCl_3$
500 MHz

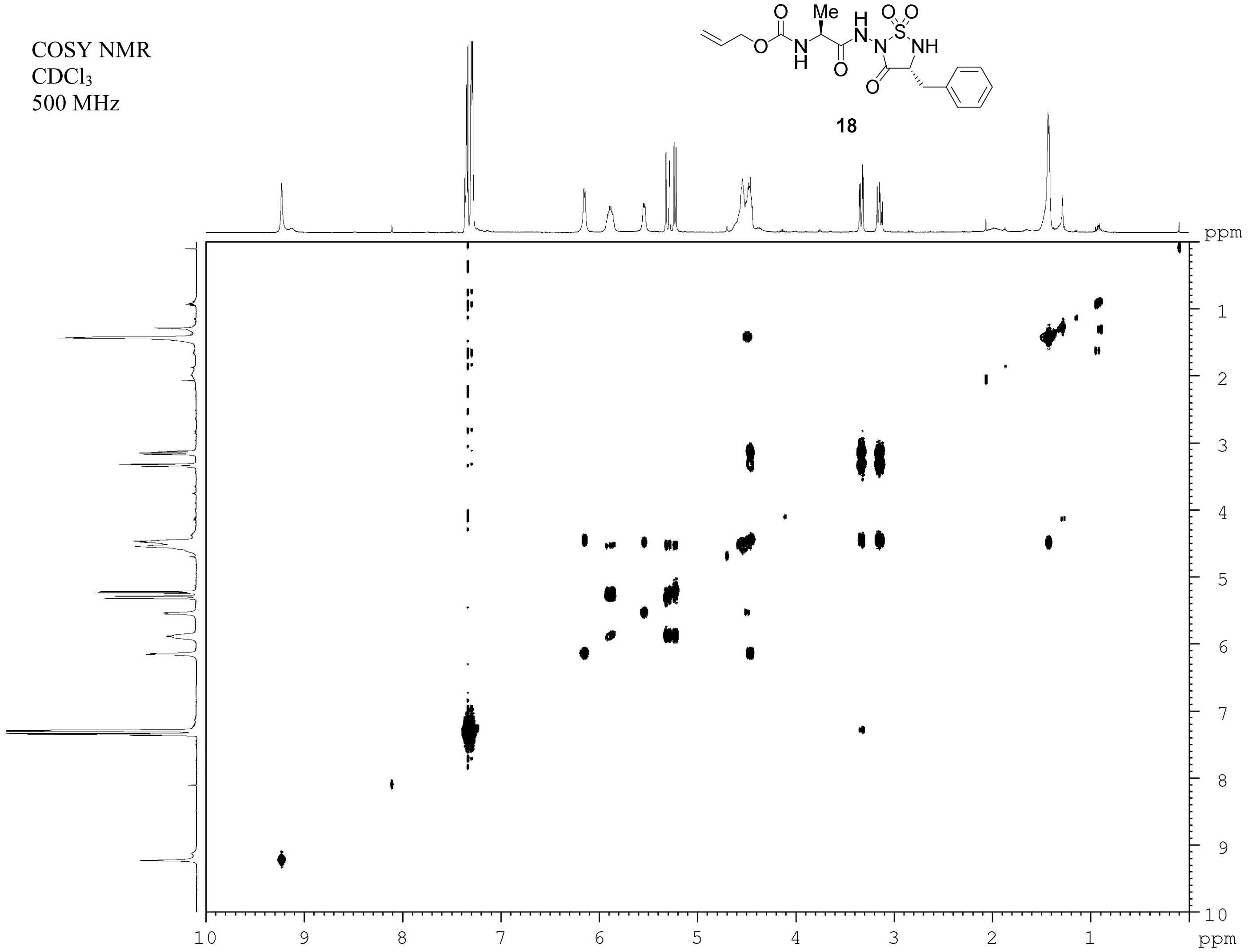

HSQC NMR
$CDCl_3$
500 MHz

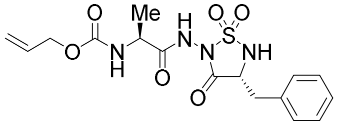

18

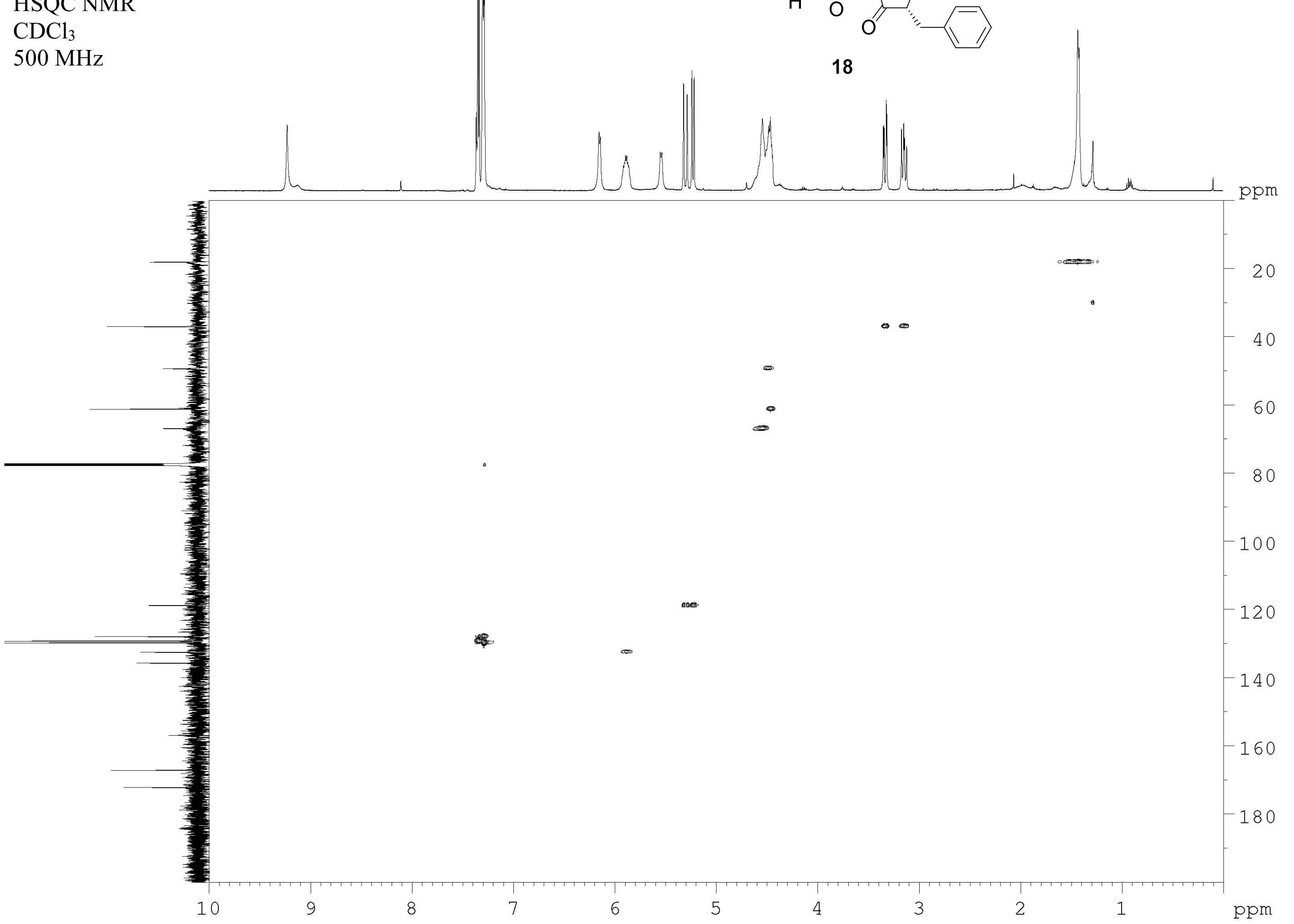

HMBC NMR
$CDCl_3$
500 MHz

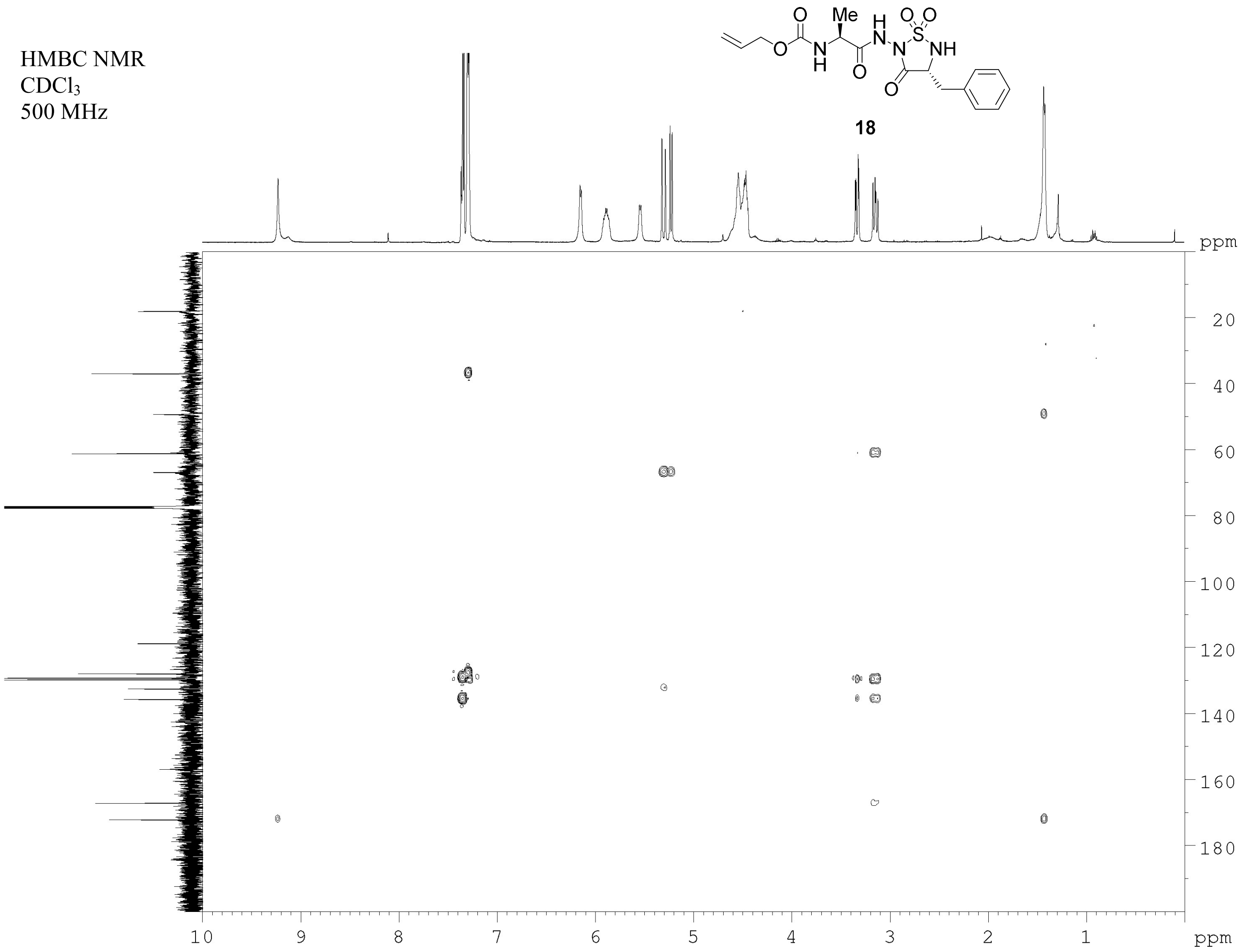

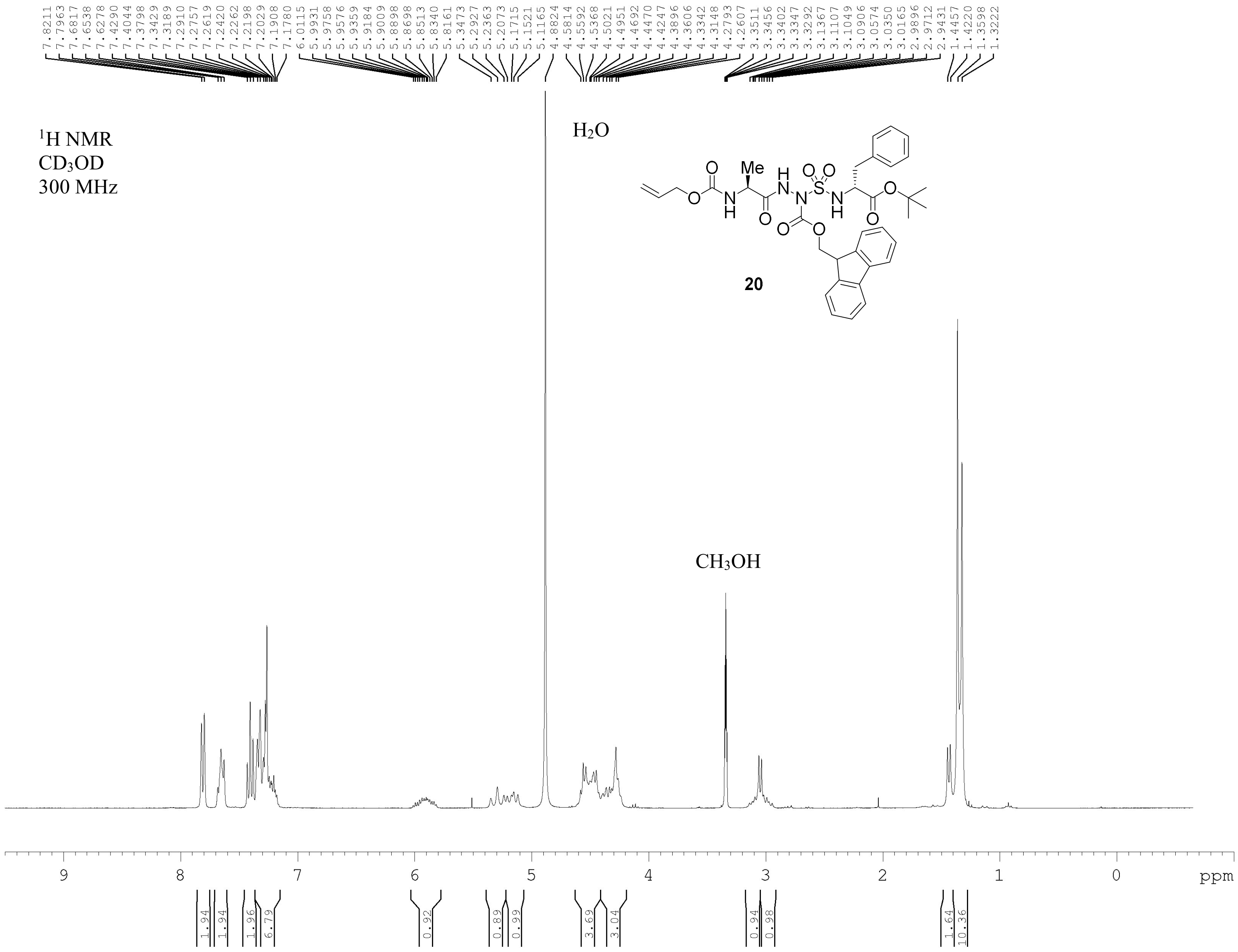

7.8211 7.7963 7.6817 7.6538 7.6278 7.4290 7.4044 7.3798 7.3429 7.3189 7.2910 7.2757 7.2619 7.2420 7.2262 7.2198 7.2029 7.1908 7.1780 6.0115 5.9931 5.9758 5.9576 5.9359 5.9184 5.9009 5.8898 5.8698 5.8513 5.8340 5.8161 5.3473 5.2927 5.2363 5.2073 5.1715 5.1521 5.1165 4.8824 4.5814 4.5592 4.5368 4.5021 4.4951 4.4692 4.4470 4.4247 4.3896 4.3606 4.3342 4.3148 4.2793 4.2607 3.3511 3.3456 3.3402 3.3347 3.3292 3.1367 3.1107 3.1049 3.0906 3.0574 3.0350 3.0165 2.9896 2.9712 2.9431 1.4457 1.4220 1.3598 1.3222
^{1}H NMR
CD_3OD
300 MHz
H_2O
Me
H
N
O
S
20
CH_3OH
9 8 7 6 5 4 3 2 1 0 ppm
1.94 1.94 1.96 6.79 0.92 0.89 0.99 3.69 3.04 0.94 0.98 1.64 10.36

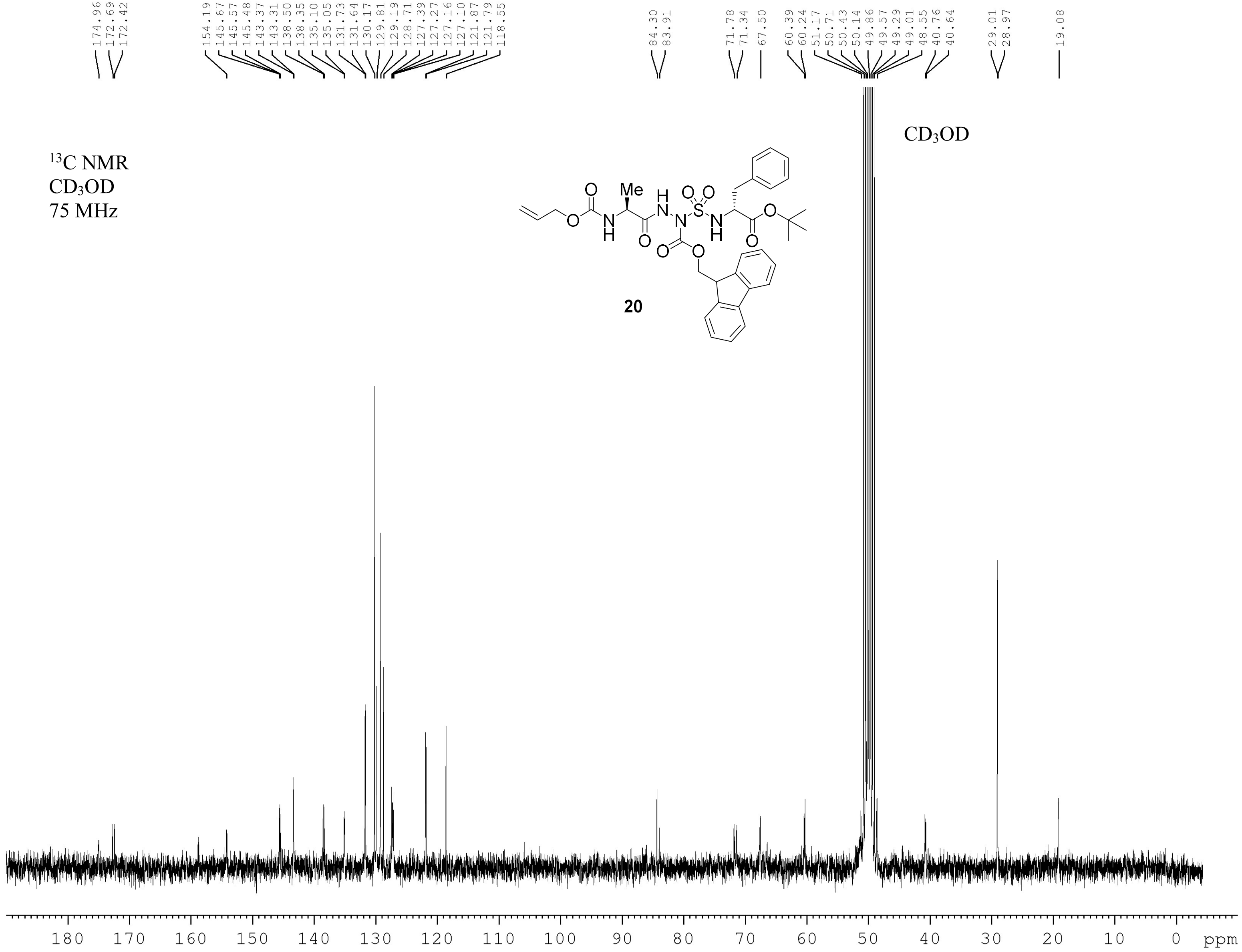
174.96
172.69
172.42
154.19
145.67
145.57
145.48
143.37
143.31
138.50
138.35
135.10
135.05
131.73
131.64
130.17
129.81
129.19
128.71
127.39
127.27
127.16
127.10
121.87
121.79
118.55
84.30
83.91
71.78
71.34
67.50
60.39
60.24
51.17
50.71
50.43
50.14
49.86
49.57
49.29
49.01
48.55
40.76
40.64
29.01
28.97
19.08
CD3OD
13C NMR
CD3OD
75 MHz
Me
20
180 170 160 150 140 130 120 110 100 90 80 70 60 50 40 30 20 10 0 ppm

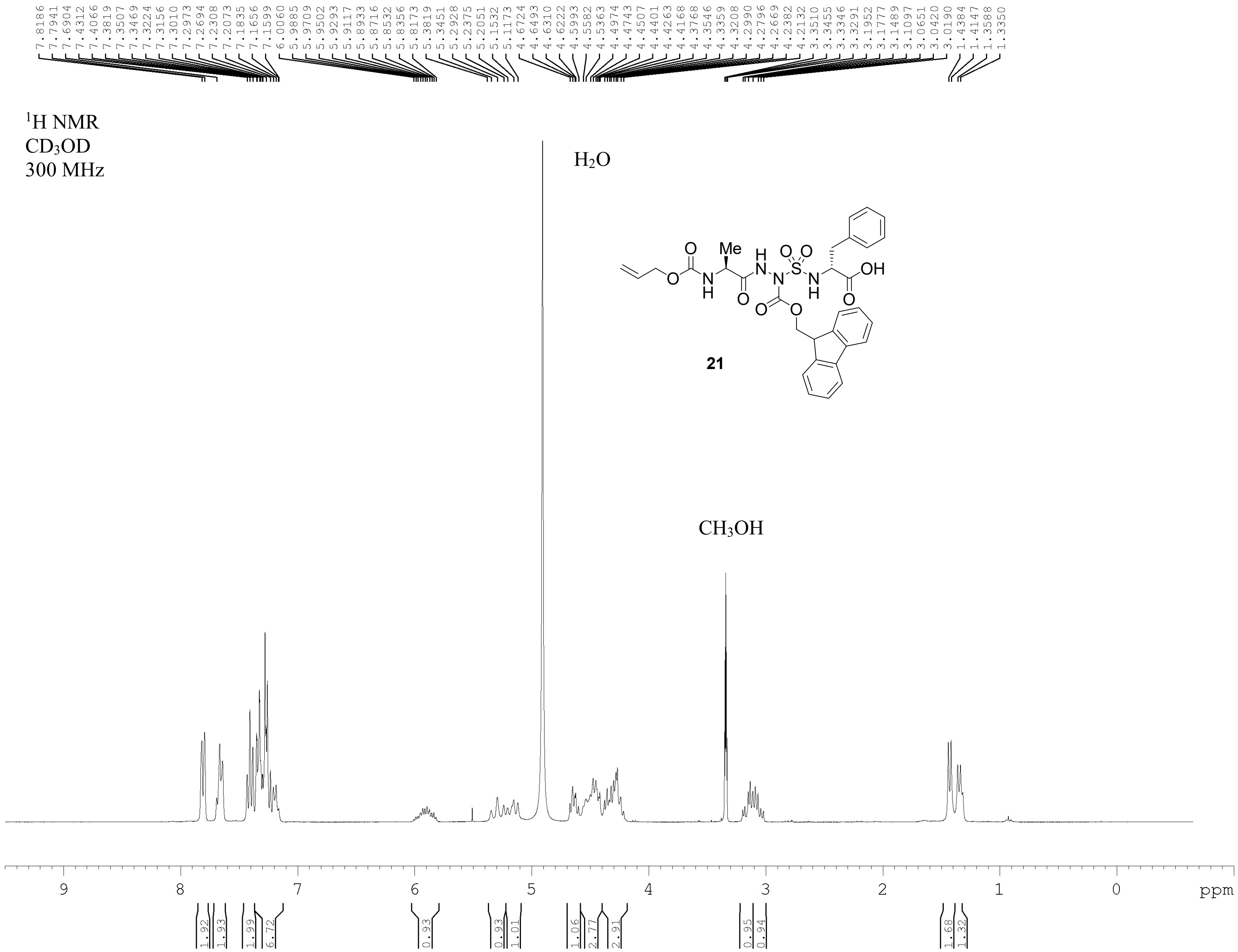
7.8186
7.7941
7.6904
7.4312
7.4066
7.3819
7.3507
7.3469
7.3224
7.3156
7.3010
7.2973
7.2694
7.2308
7.2073
7.1835
7.1656
7.1599
6.0060
5.9885
5.9709
5.9502
5.9293
5.9117
5.8933
5.8716
5.8532
5.8356
5.8173
5.3819
5.3451
5.2928
5.2375
5.2051
5.1532
5.1173
4.6724
4.6493
4.6310
4.6222
4.5993
4.5582
4.5363
4.4974
4.4743
4.4507
4.4401
4.4263
4.4168
4.3768
4.3546
4.3359
4.3208
4.2990
4.2796
4.2669
4.2382
4.2132
3.3510
3.3455
3.3346
3.3291
3.1952
3.1777
3.1489
3.1097
3.0651
3.0420
3.0190
1.4384
1.4147
1.3588
1.3350
¹H NMR
CD3OD
300 MHz
H2O
CH3OH
Me
H
O
OH
N
S
NH
21
9
8
7
6
5
4
3
2
1
0
ppm
1.92
1.93
1.99
6.72
0.93
0.93
1.01
1.06
2.77
2.91
0.95
0.94
1.68
1.32

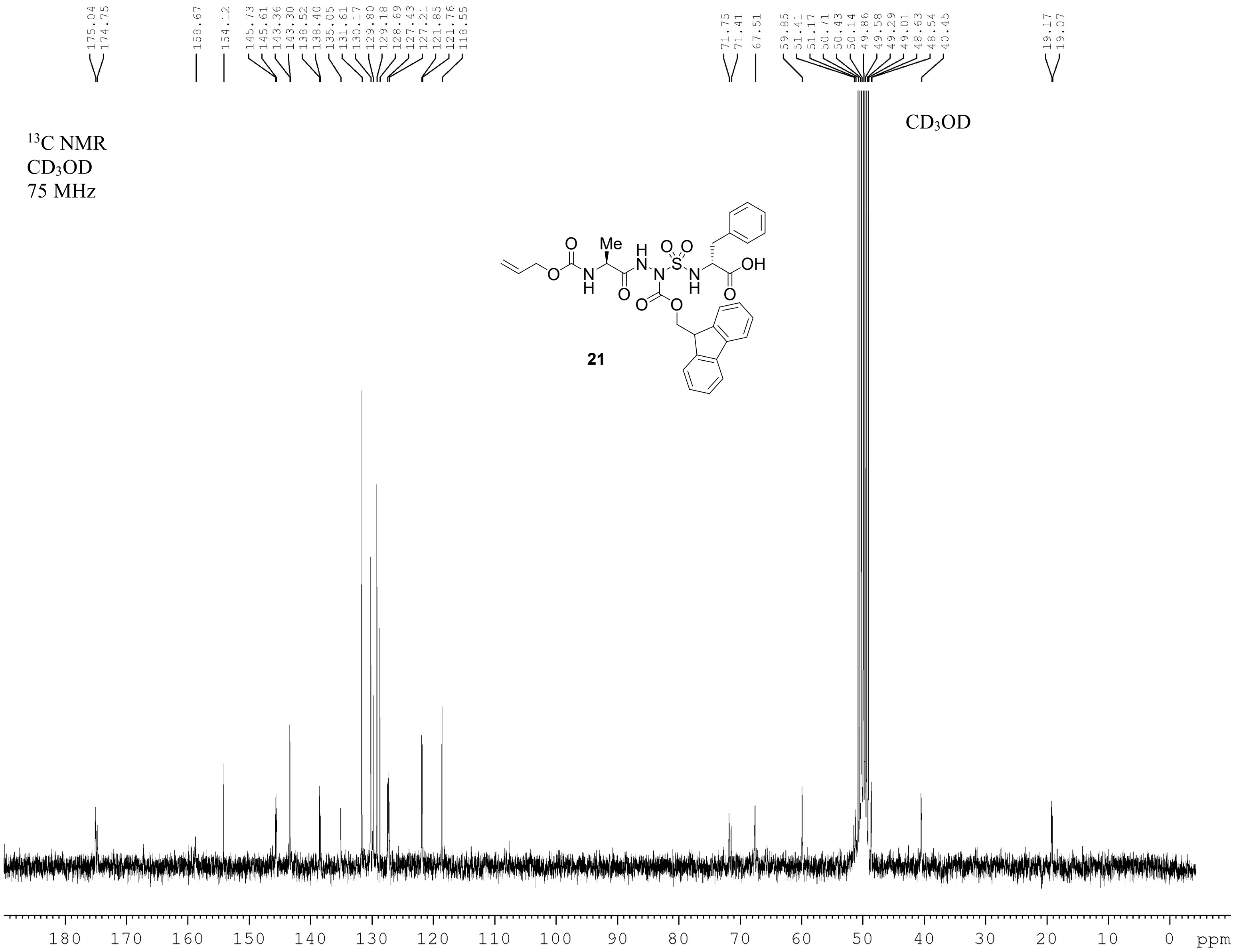
175.04
174.75
158.67
154.12
145.73
145.61
143.36
143.30
138.52
138.40
135.05
131.61
130.17
129.80
129.18
128.69
127.43
127.21
121.85
121.76
118.55
71.75
71.41
67.51
59.85
51.41
51.17
50.71
50.43
50.14
49.86
49.58
49.29
49.01
48.63
48.54
40.45
19.17
19.07
13C NMR
CD3OD
75 MHz
CD3OD
Me
OH
21
180 170 160 150 140 130 120 110 100 90 80 70 60 50 40 30 20 10 0 ppm

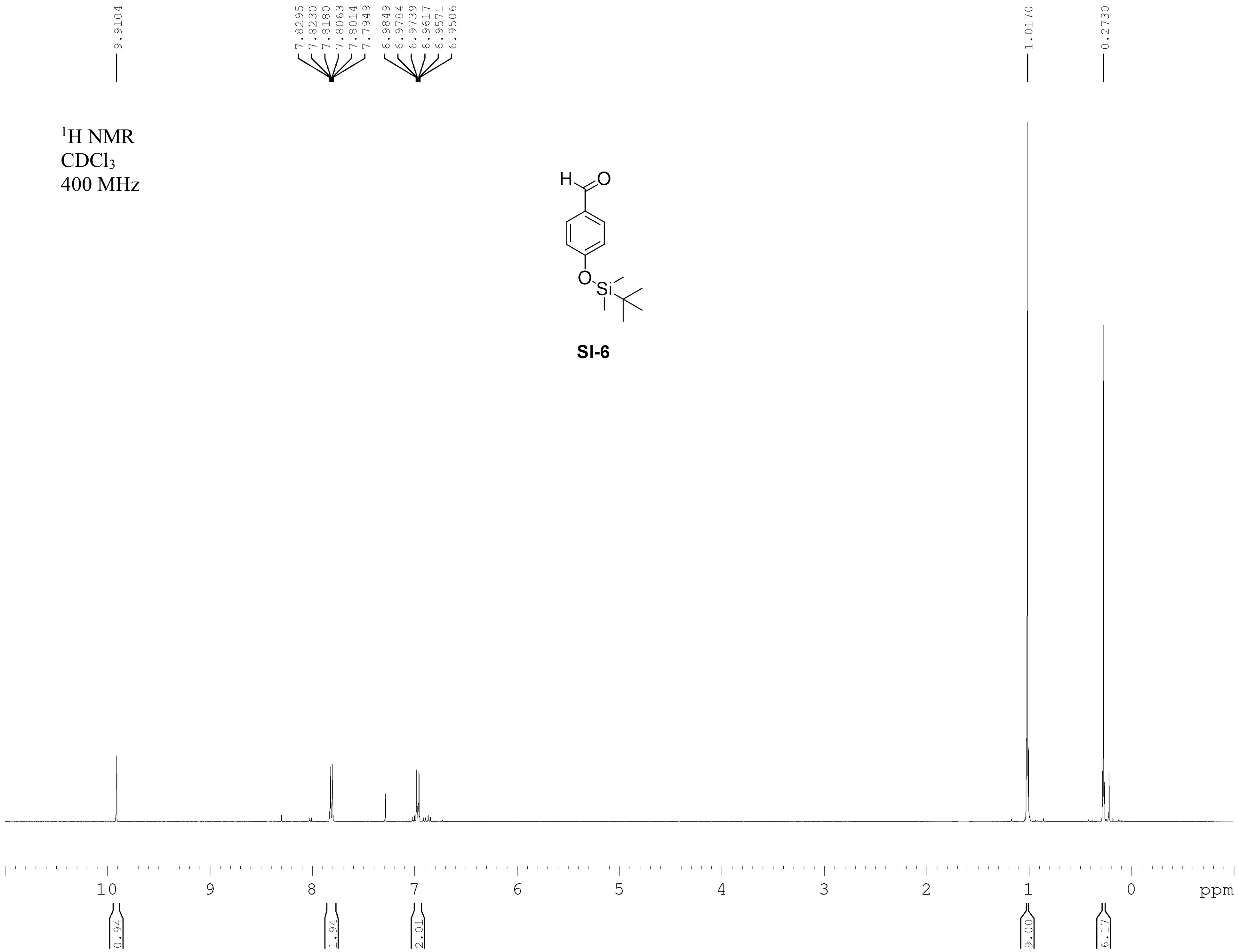

^{1}H NMR
CDCl$_3$
400 MHz
SI-6
9.9104
7.8295
7.8230
7.8180
7.8063
7.8014
7.7949
6.9849
6.9784
6.9739
6.9617
6.9571
6.9506
1.0170
0.2730
10 9 8 7 6 5 4 3 2 1 0 ppm
0.94
1.94
2.01
9.00
6.17

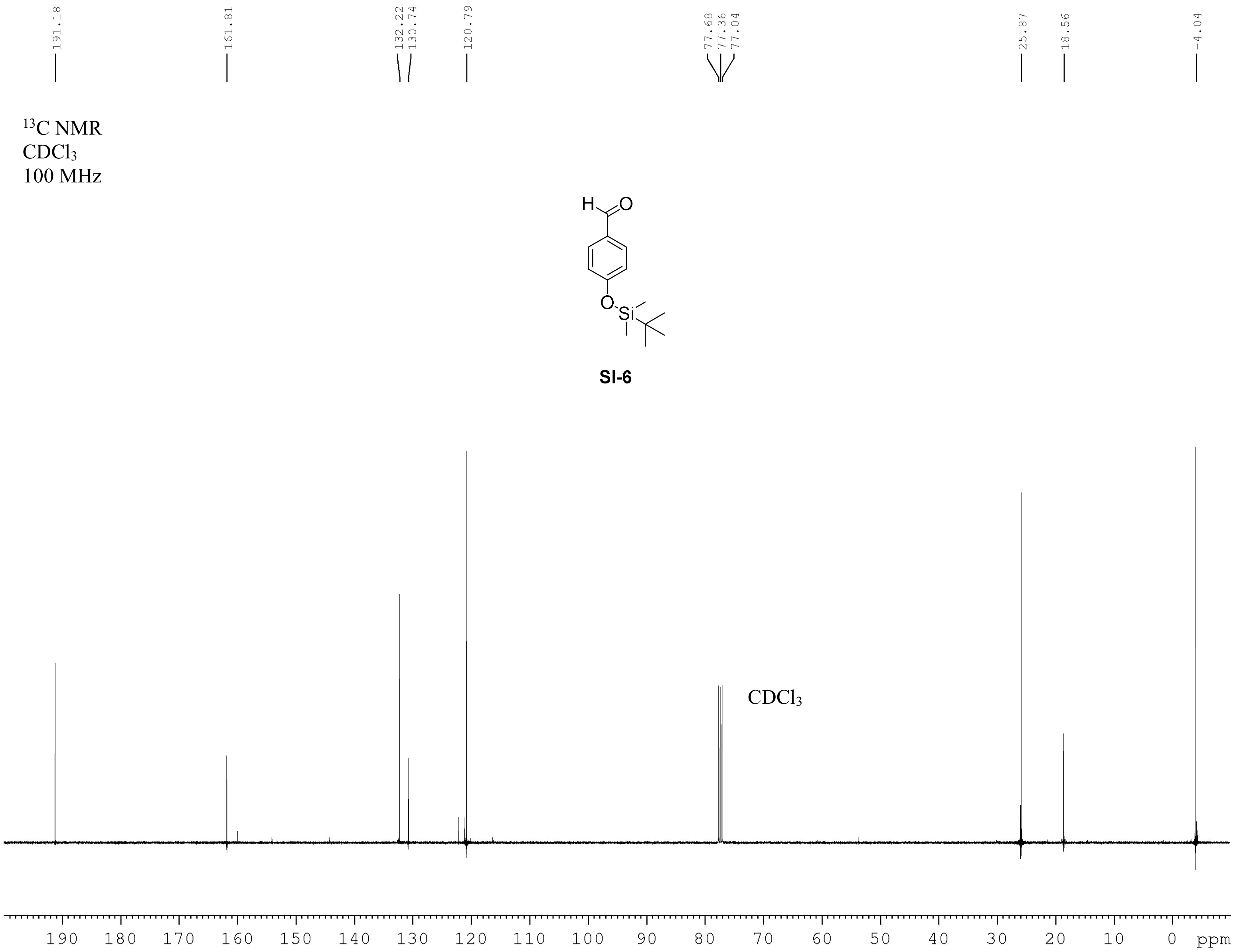
191.18
161.81
132.22
130.74
120.79
77.68
77.36
77.04
25.87
18.56
-4.04
^{13}C NMR
$CDCl_3$
100 MHz
SI-6
$CDCl_3$
190 180 170 160 150 140 130 120 110 100 90 80 70 60 50 40 30 20 10 0 ppm

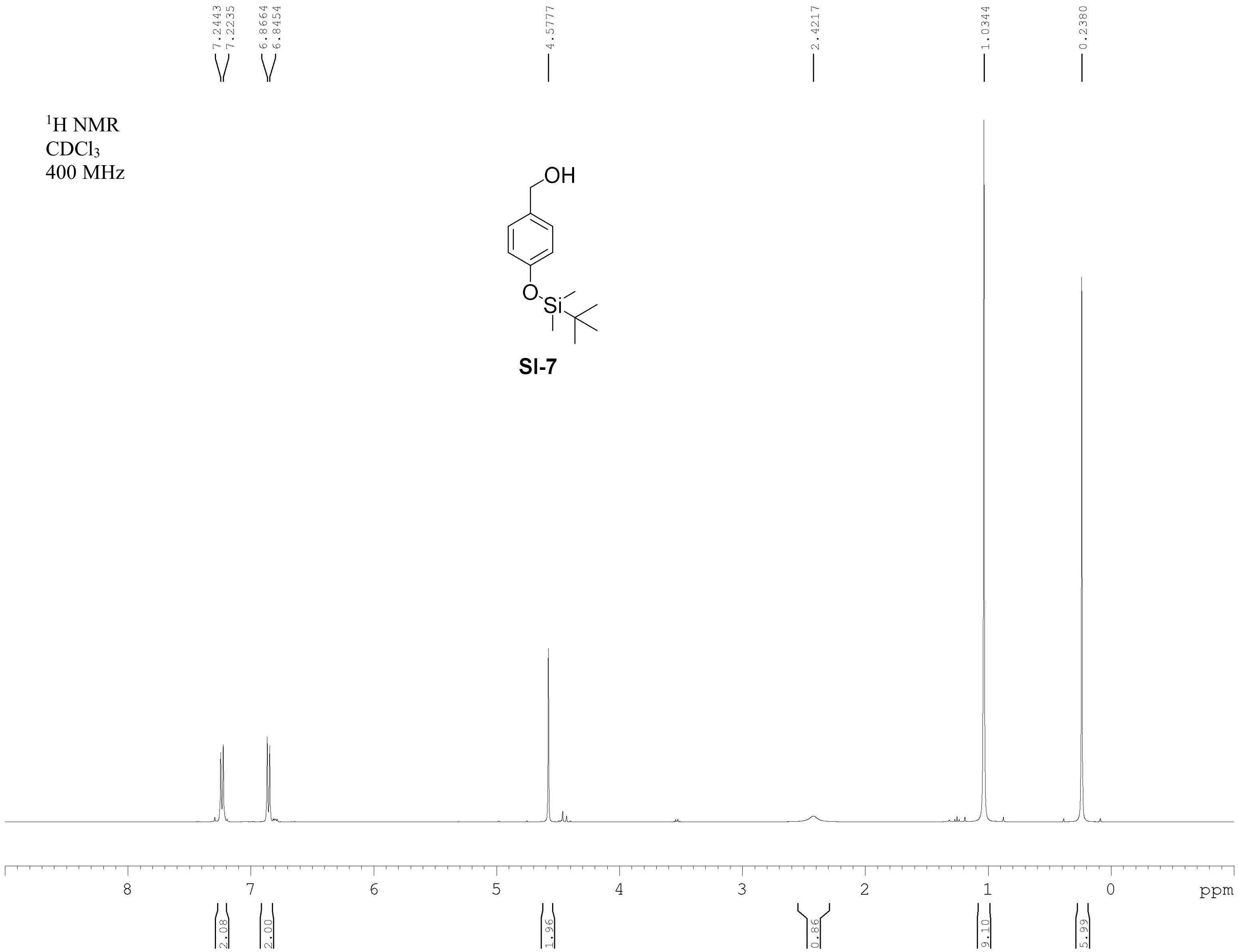
^{1}H NMR
$CDCl_3$
400 MHz
OH
O
Si
SI-7
7.2443
7.2235
6.8664
6.8454
4.5777
2.4217
1.0344
0.2380
8
7
6
5
4
3
2
1
0
ppm
2.08
2.00
1.96
0.86
9.10
5.99

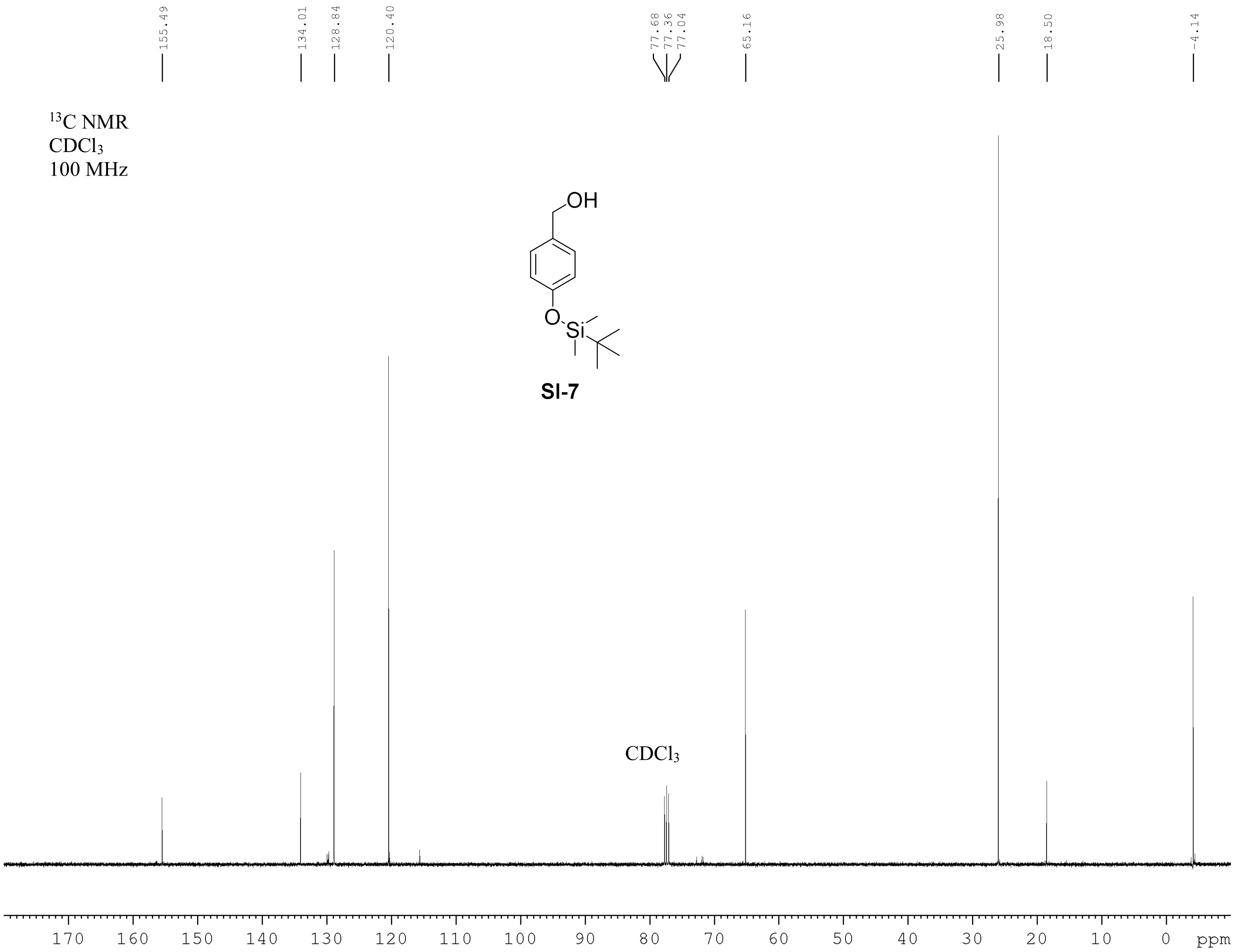

155.49
134.01
128.84
120.40
77.68
77.36
77.04
65.16
25.98
18.50
-4.14
^{13}C NMR
CDCl$_3$
100 MHz
OH
O
Si
SI-7
CDCl$_3$
170 160 150 140 130 120 110 100 90 80 70 60 50 40 30 20 10 0 ppm

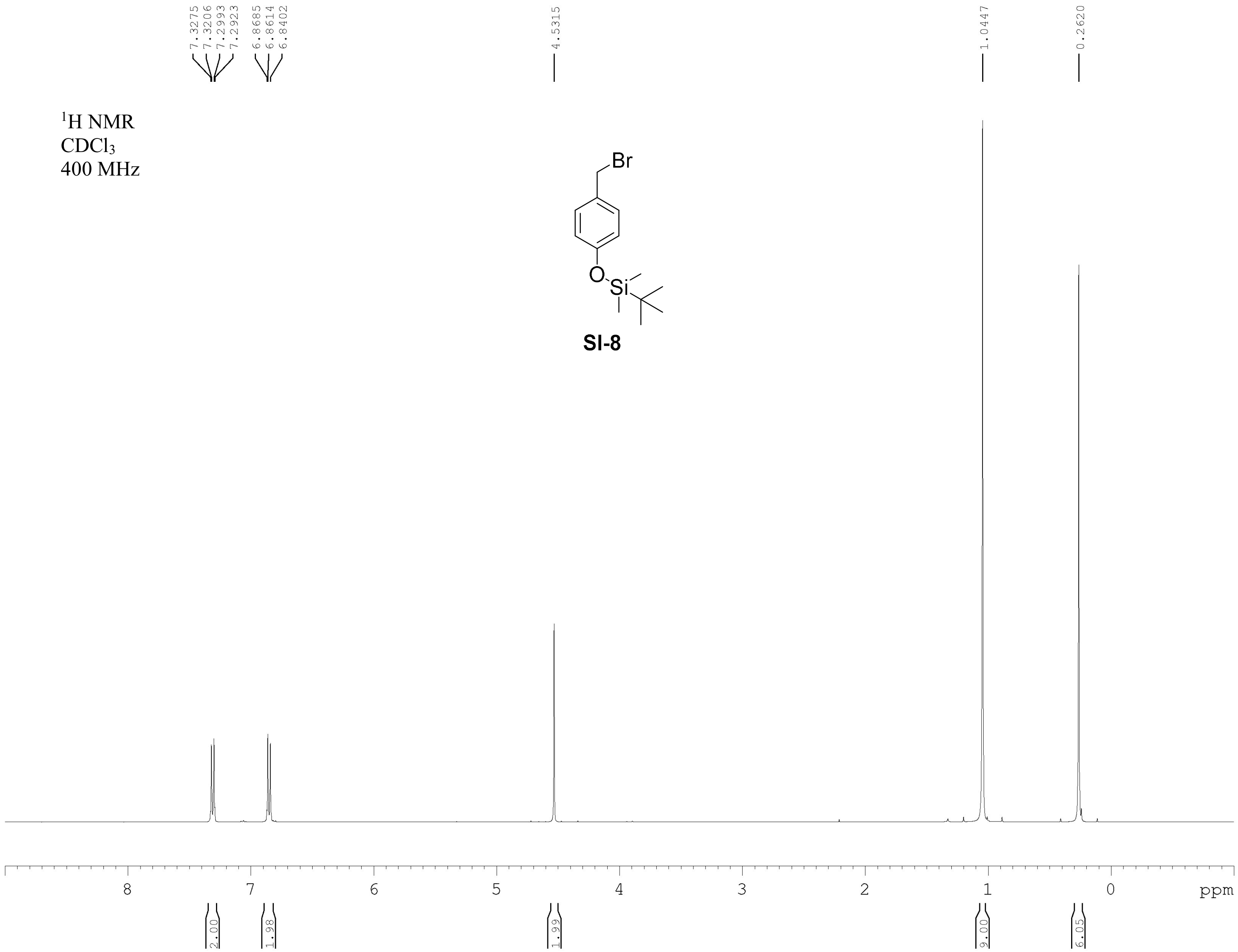
7.3275
7.3206
7.2993
7.2923
6.8685
6.8614
6.8402
4.5315
1.0447
0.2620
^{1}H NMR
$CDCl_3$
400 MHz
Br
O
Si
SI-8
8
7
6
5
4
3
2
1
0
ppm
2.00
1.98
1.99
9.00
6.05

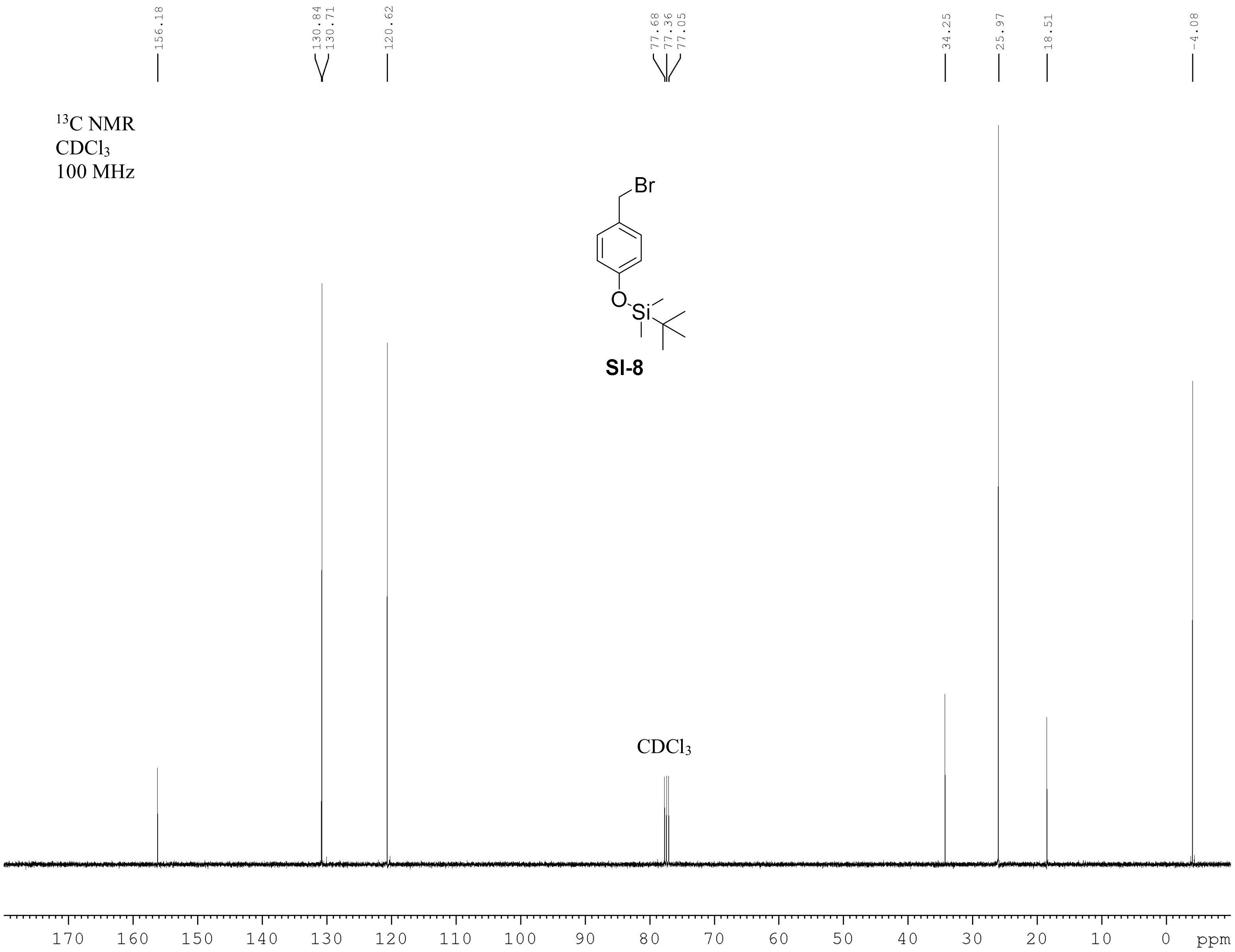
156.18
130.84
130.71
120.62
77.68
77.36
77.05
34.25
25.97
18.51
-4.08
^{13}C NMR
$CDCl_3$
100 MHz
Br
O
Si
SI-8
$CDCl_3$
170 160 150 140 130 120 110 100 90 80 70 60 50 40 30 20 10 0 ppm

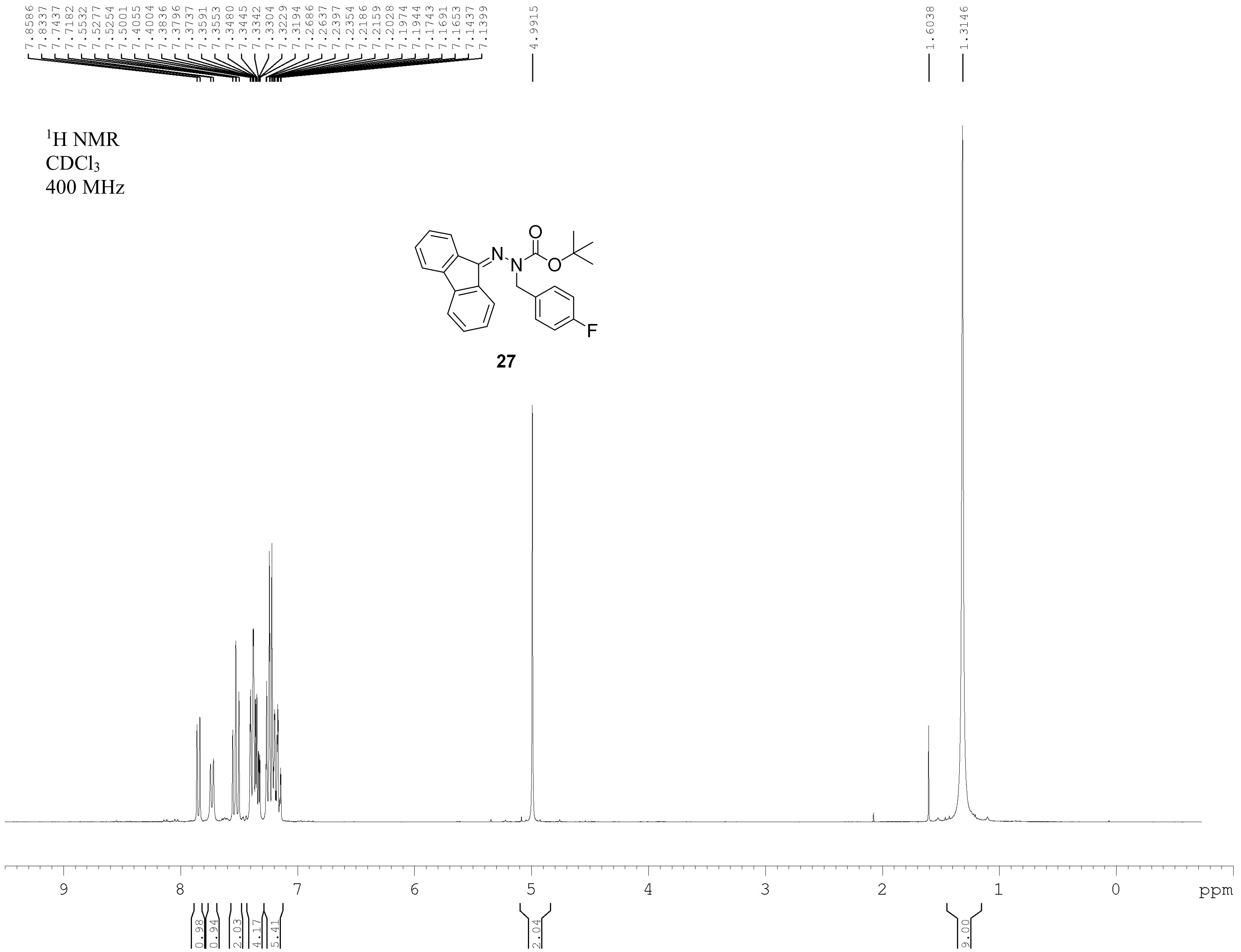
7.8586
7.8337
7.7437
7.7182
7.5532
7.5277
7.5254
7.5001
7.4055
7.4004
7.3836
7.3796
7.3737
7.3591
7.3553
7.3480
7.3445
7.3342
7.3304
7.3229
7.3194
7.2686
7.2637
7.2397
7.2354
7.2186
7.2159
7.2028
7.1974
7.1944
7.1743
7.1691
7.1653
7.1437
7.1399
4.9915
1.6038
1.3146
^{1}H NMR
$CDCl_3$
400 MHz
O
N
N
O
F
27
9
8
7
6
5
4
3
2
1
0
ppm
0.98
0.94
2.03
4.17
5.41
2.04
9.00

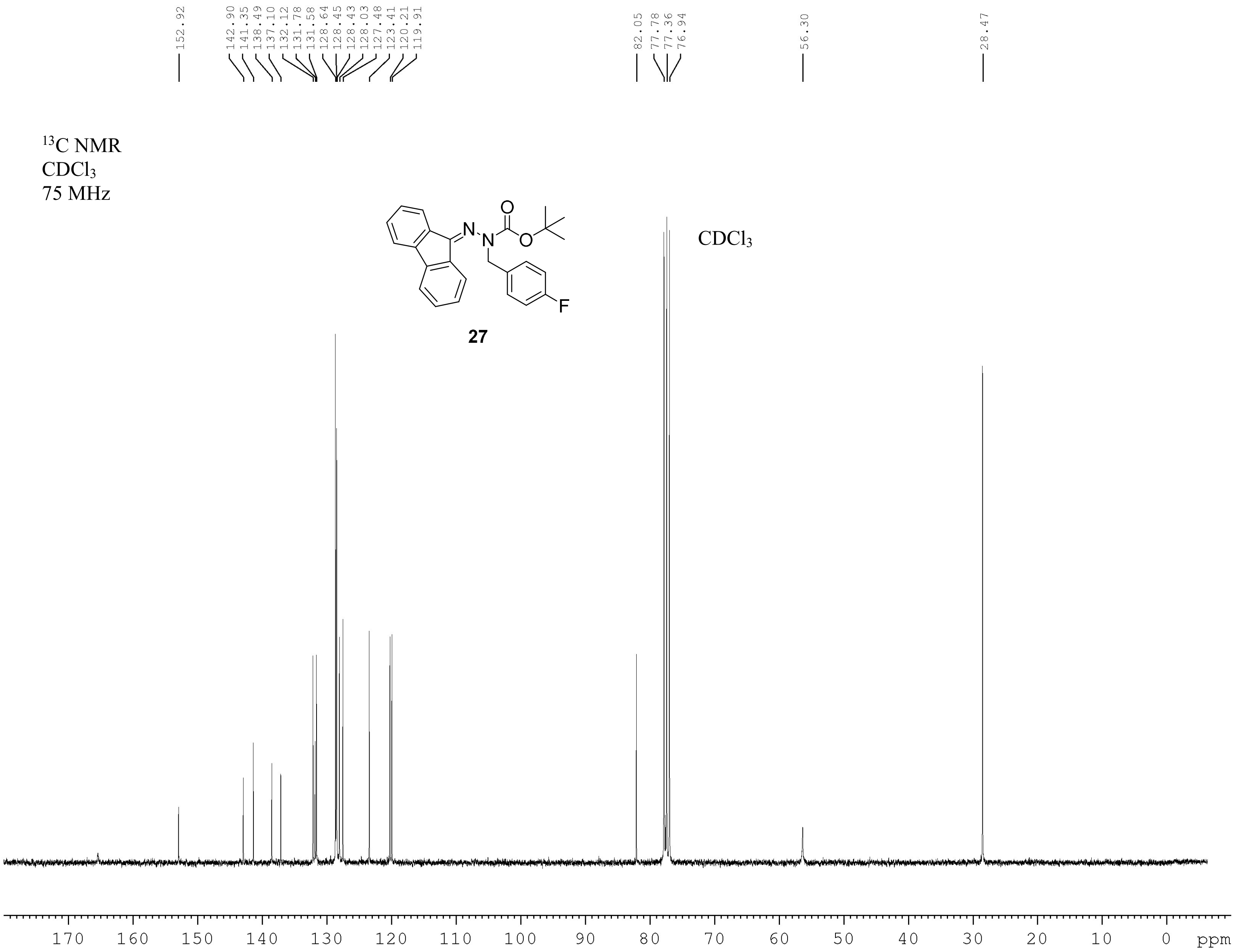

152.92
142.90
141.35
138.49
137.10
132.12
131.78
131.58
128.64
128.45
128.43
128.03
127.48
123.41
120.21
119.91
82.05
77.78
77.36
76.94
56.30
28.47
13C NMR
CDCl3
75 MHz
O
N
N
O
F
27
CDCl3
170
160
150
140
130
120
110
100
90
80
70
60
50
40
30
20
10
0
ppm

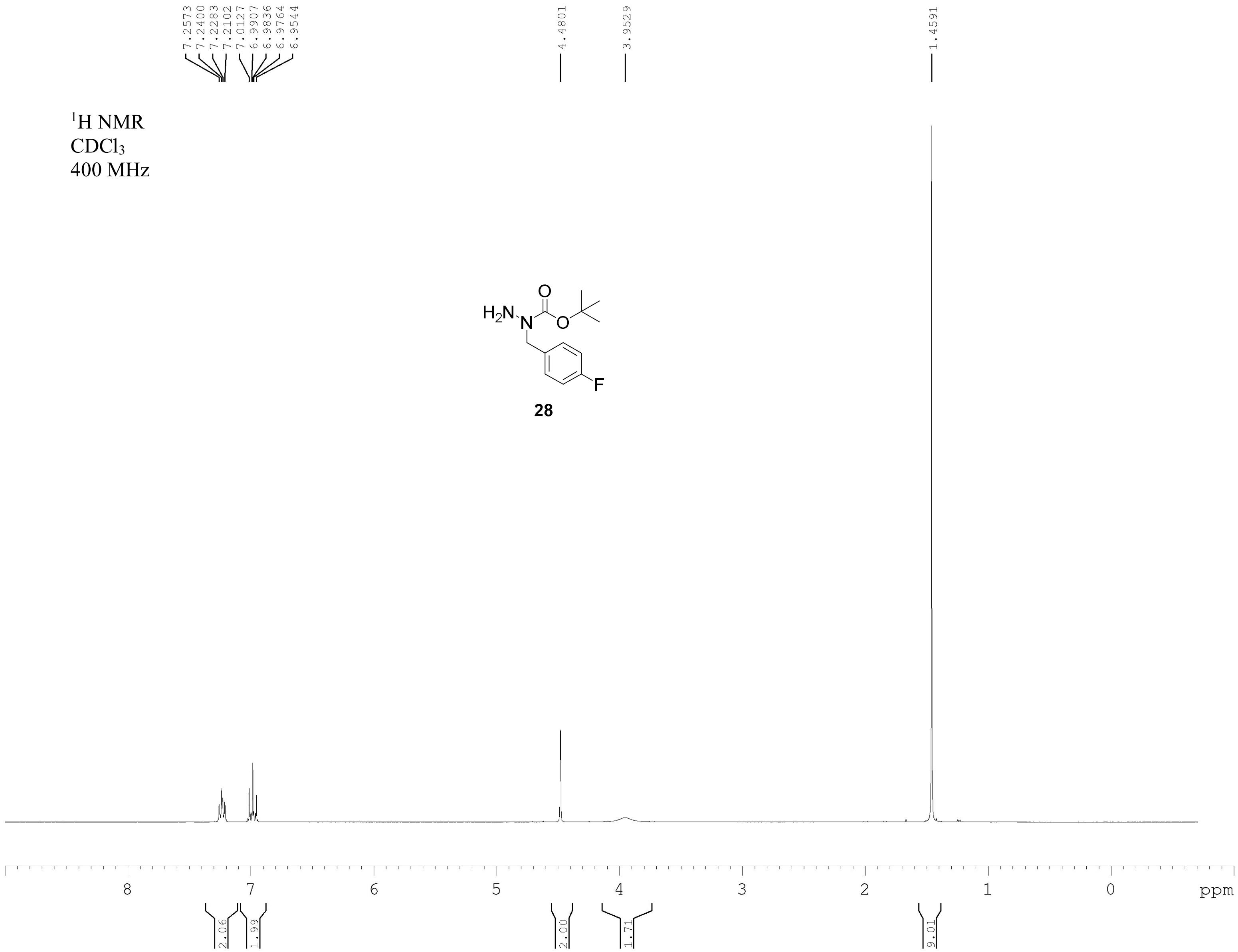
1H NMR
CDCl3
400 MHz
7.2573
7.2400
7.2283
7.2102
7.0127
6.9907
6.9836
6.9764
6.9544
4.4801
3.9529
1.4591
8
7
6
5
4
3
2
1
0
ppm
2.06
1.99
2.00
1.71
9.01

28

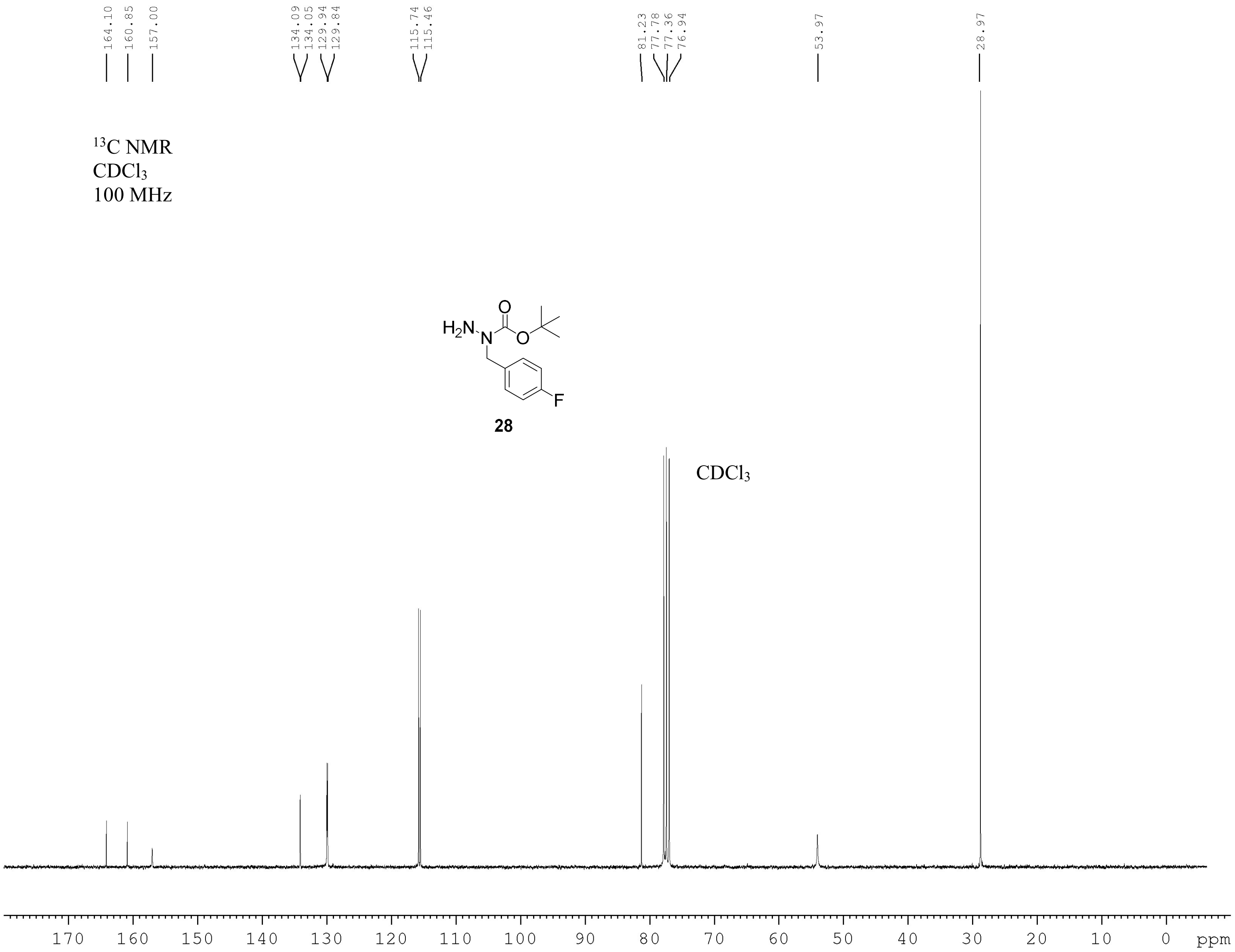
164.10
160.85
157.00
134.09
134.05
129.94
129.84
115.74
115.46
81.23
77.78
77.36
76.94
53.97
28.97
13C NMR
CDCl3
100 MHz
H2N
N
O
O
F
28
CDCl3
170
160
150
140
130
120
110
100
90
80
70
60
50
40
30
20
10
0
ppm

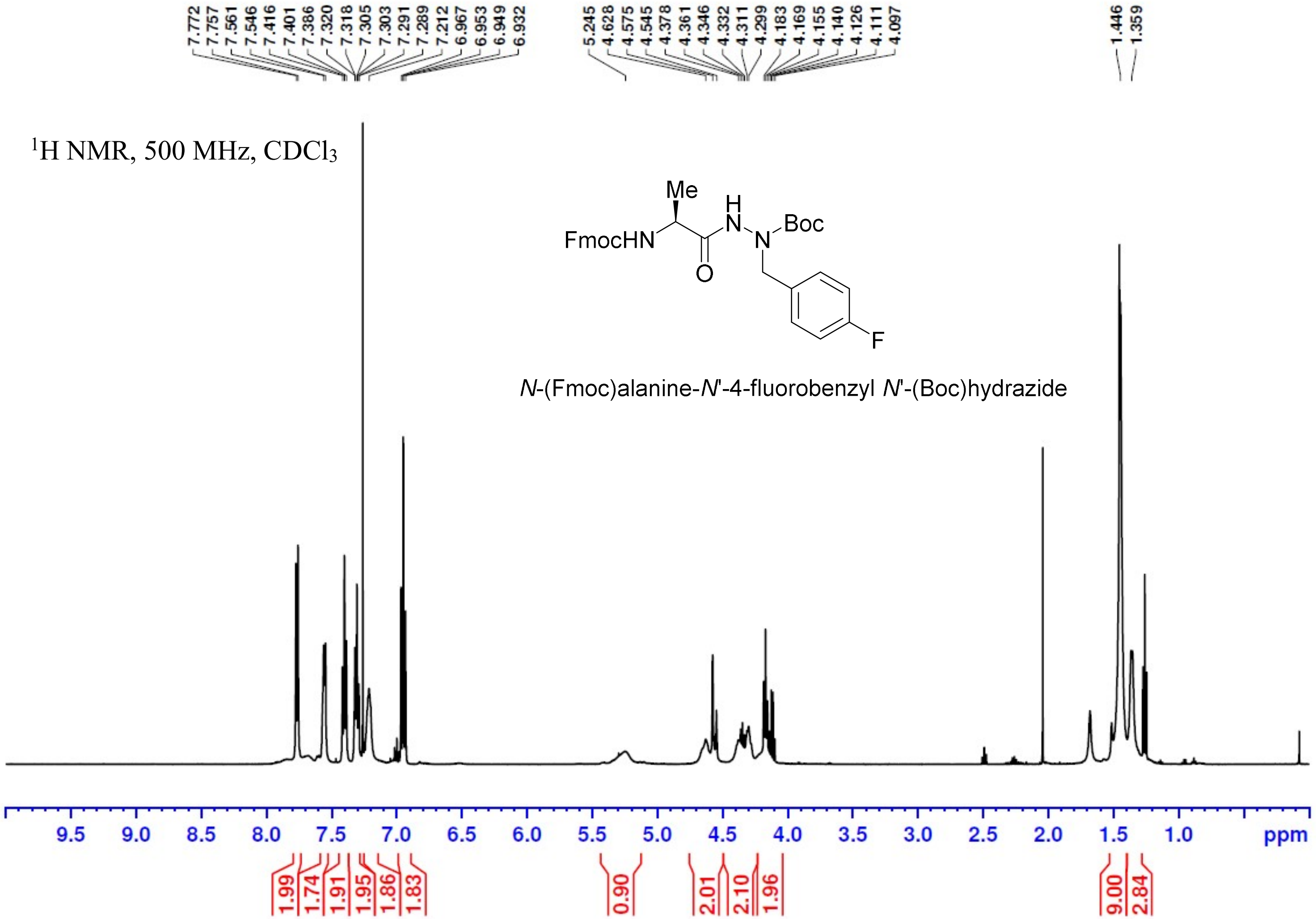
7.772
7.757
7.561
7.546
7.416
7.401
7.386
7.320
7.318
7.305
7.303
7.291
7.289
7.212
6.967
6.953
6.949
6.932
5.245
4.628
4.575
4.545
4.378
4.361
4.346
4.332
4.311
4.299
4.183
4.169
4.155
4.140
4.126
4.111
4.097
1.446
1.359
1H NMR, 500 MHz, CDCl3
Me
H
N
Boc
FmocHN
N
O
F
N-(Fmoc)alanine-N'-4-fluorobenzyl N'-(Boc)hydrazide
9.5
9.0
8.5
8.0
7.5
7.0
6.5
6.0
5.5
5.0
4.5
4.0
3.5
3.0
2.5
2.0
1.5
1.0
ppm
1.99
1.74
1.91
1.95
1.86
1.83
0.90
2.01
2.10
1.96
9.00
2.84

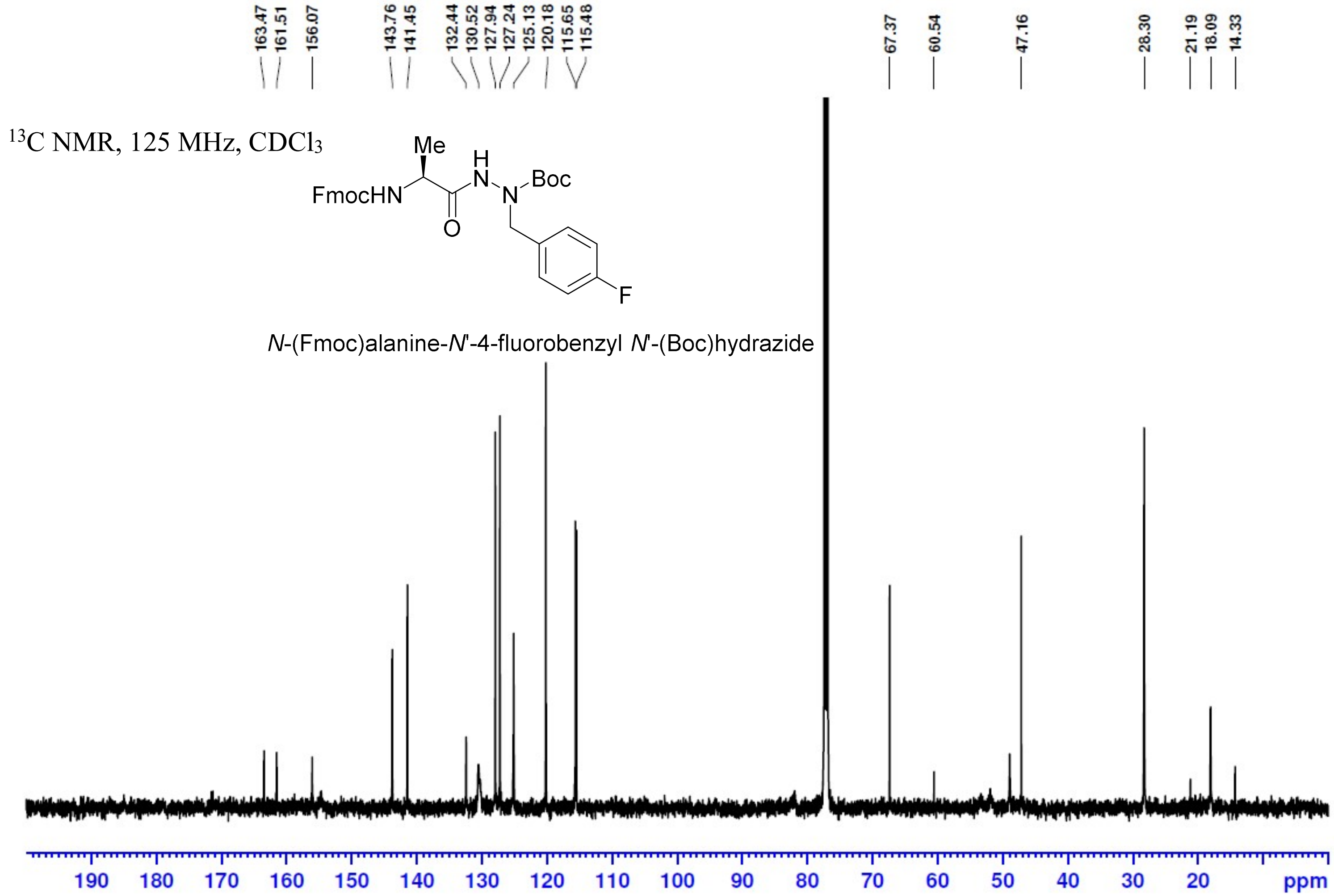
163.47
161.51
156.07
143.76
141.45
132.44
130.52
127.94
127.24
125.13
120.18
115.65
115.48
67.37
60.54
47.16
28.30
21.19
18.09
14.33
¹³C NMR, 125 MHz, CDCl₃
Me
H
N
N
Boc
FmocHN
O
F
N-(Fmoc)alanine-N'-4-fluorobenzyl N'-(Boc)hydrazide
190
180
170
160
150
140
130
120
110
100
90
80
70
60
50
40
30
20
ppm

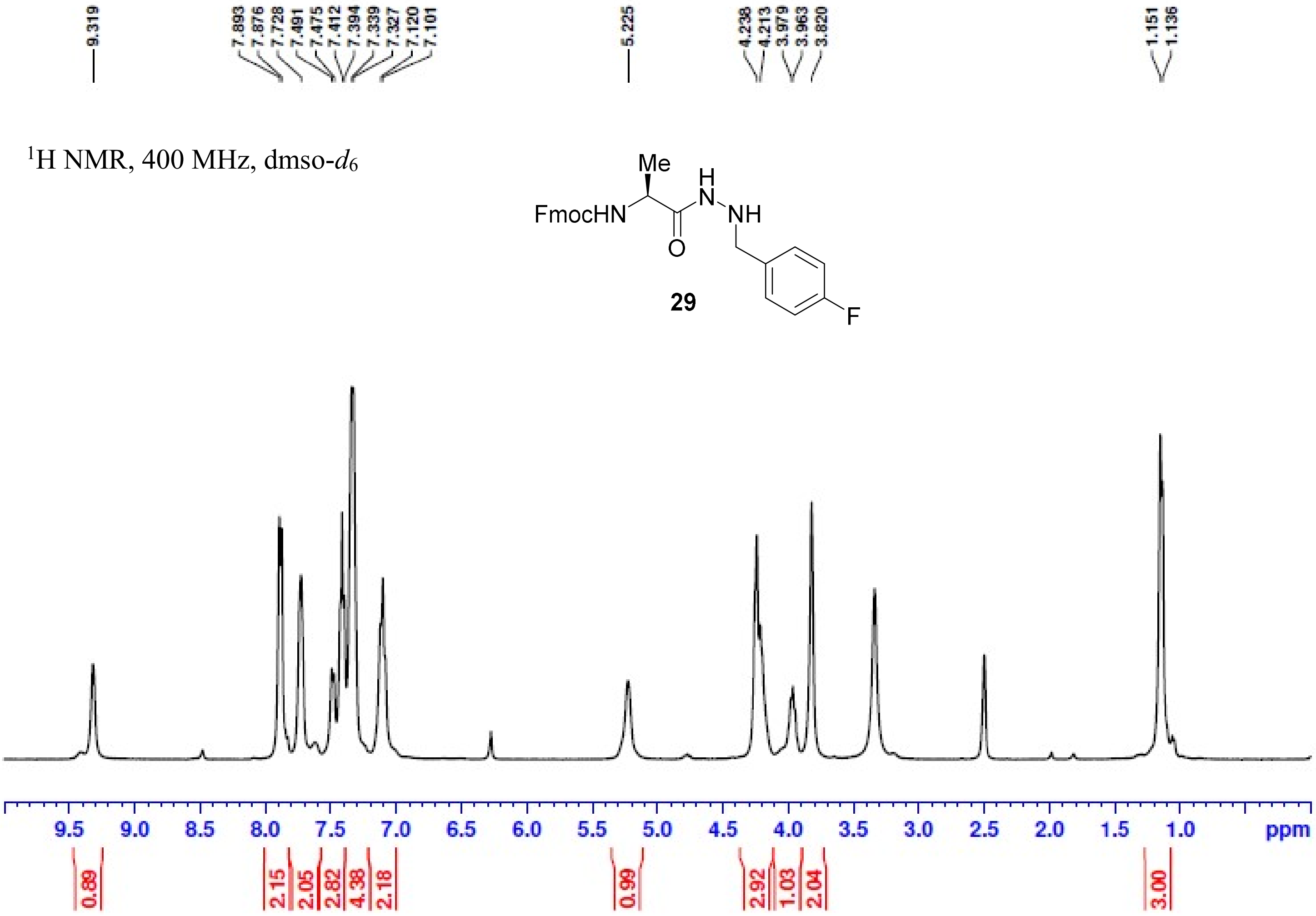

9.319
7.893 7.876 7.728 7.491 7.475 7.412 7.394 7.339 7.327 7.120 7.101
5.225
4.238 4.213 3.979 3.963 3.820
1.151 1.136
^{1}H NMR, 400 MHz, dmso-d_6
Me
FmocHN
H
N
NH
O
F
29
9.5 9.0 8.5 8.0 7.5 7.0 6.5 6.0 5.5 5.0 4.5 4.0 3.5 3.0 2.5 2.0 1.5 1.0 ppm
0.89 2.15 2.05 2.82 4.38 2.18 0.99 2.92 1.03 2.04 3.00

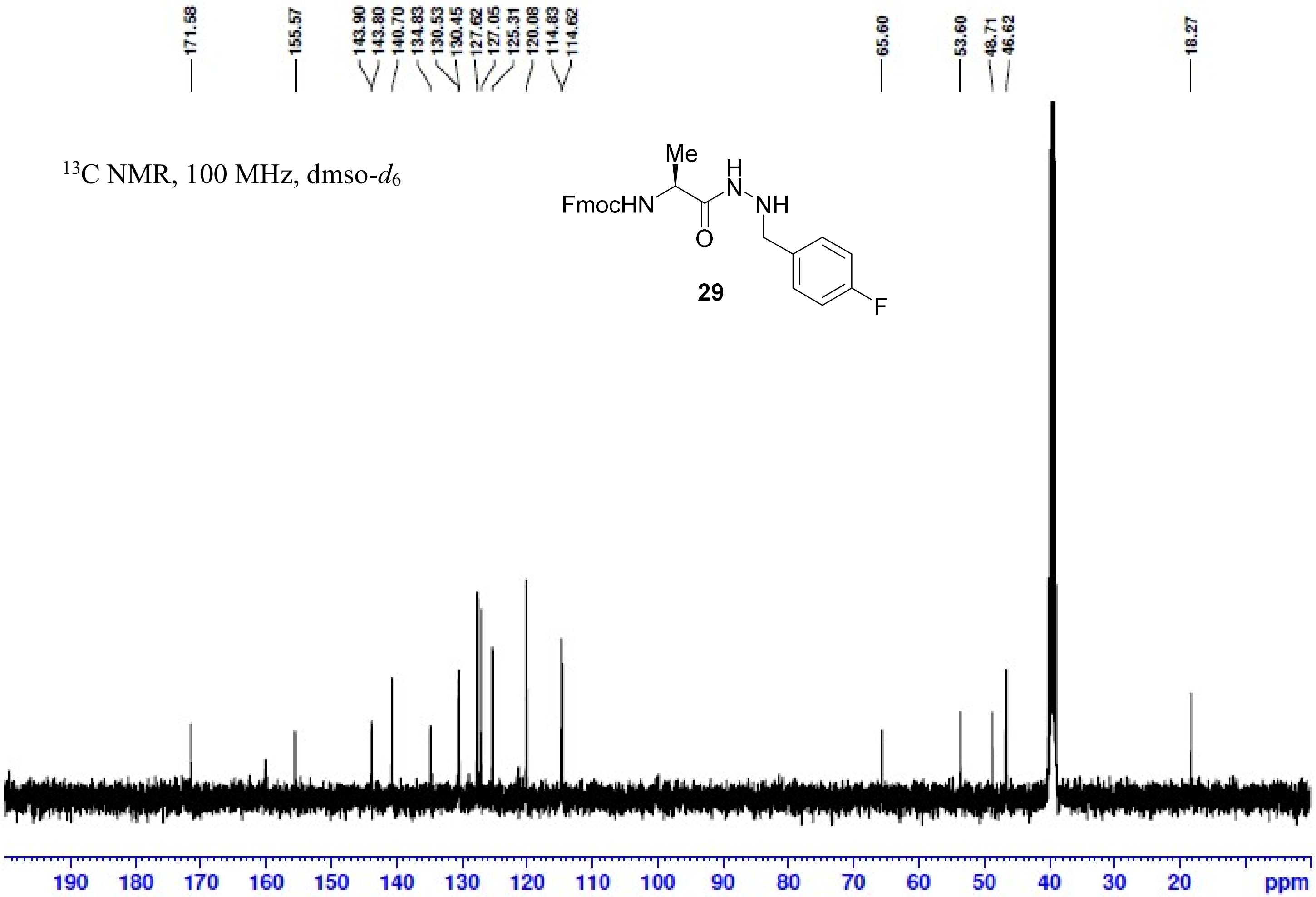

171.58
155.57
143.90
143.80
140.70
134.83
130.53
130.45
127.62
127.05
125.31
120.08
114.83
114.62
65.60
53.60
48.71
46.62
18.27
^{13}C NMR, 100 MHz, dmso-d_6
Me
FmocHN
H
N
NH
O
F
29
190 180 170 160 150 140 130 120 110 100 90 80 70 60 50 40 30 20 ppm

^{1}H NMR, 500 MHz, $CDCl_3$

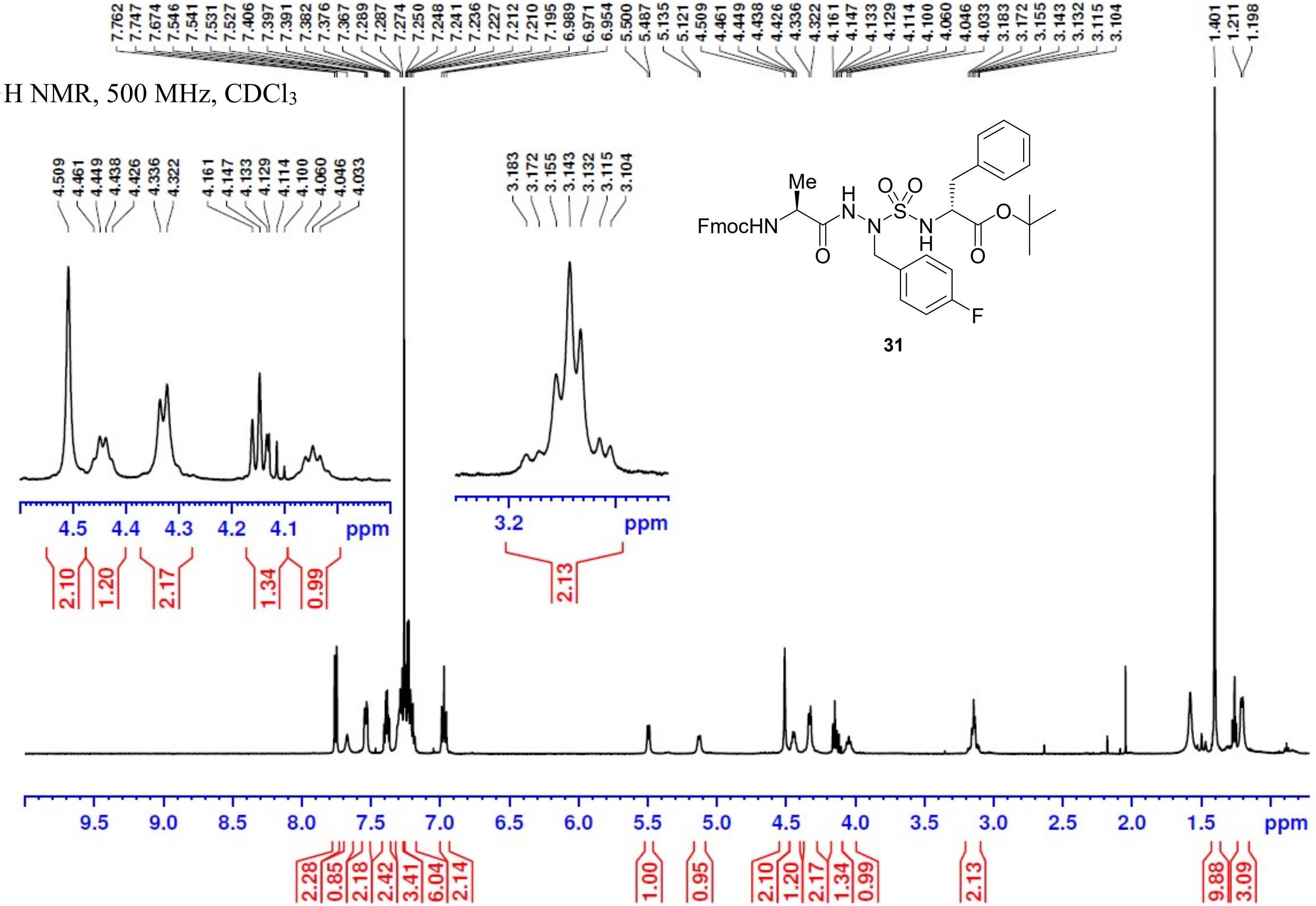

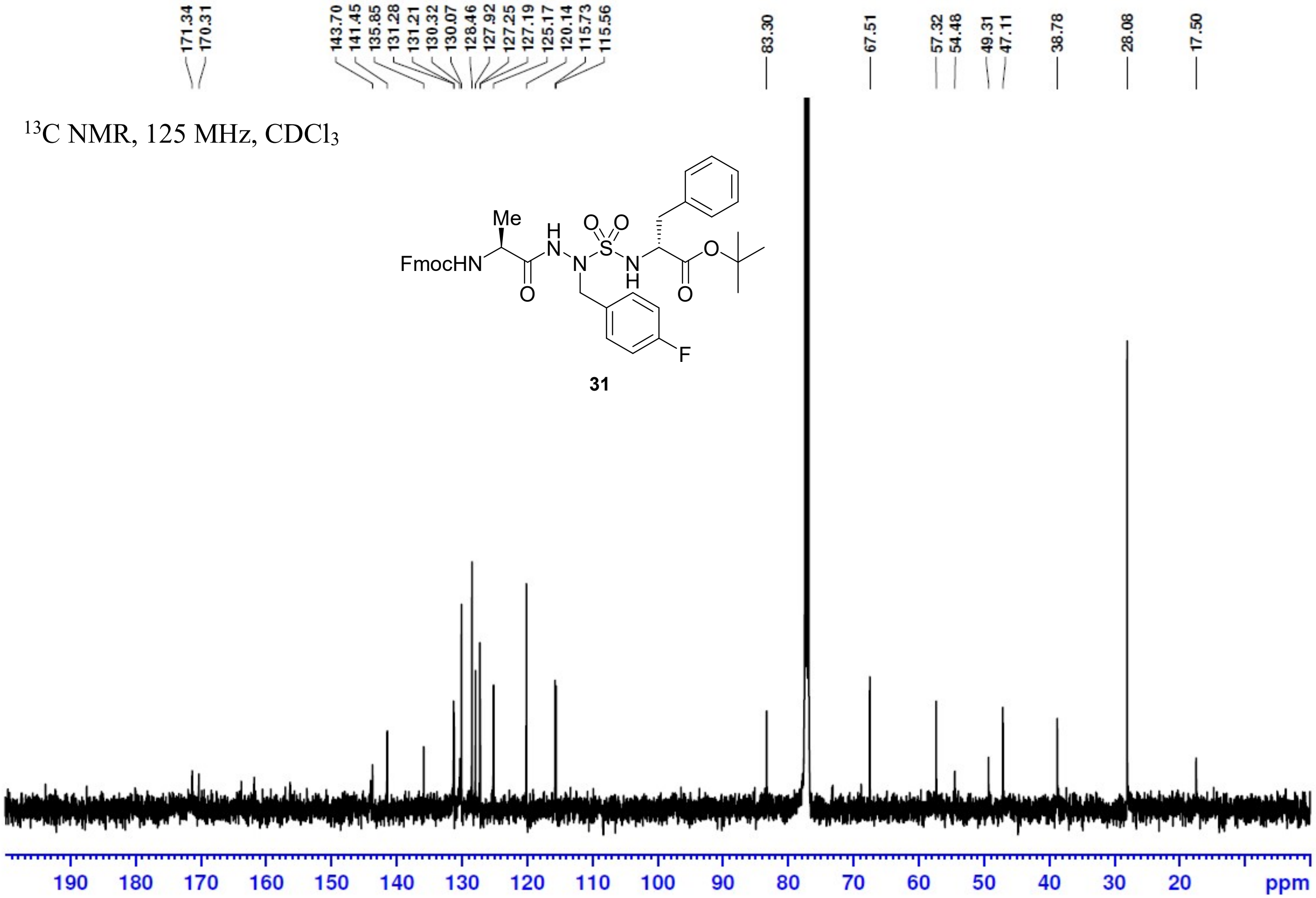

^{13}C NMR, 125 MHz, CDCl$_3$
171.34
170.31
143.70
141.45
135.85
131.28
131.21
130.32
130.07
128.46
127.92
127.25
127.19
125.17
120.14
115.73
115.56
83.30
67.51
57.32
54.48
49.31
47.11
38.78
28.08
17.50
Me
FmocHN
F
31
190 180 170 160 150 140 130 120 110 100 90 80 70 60 50 40 30 20 ppm

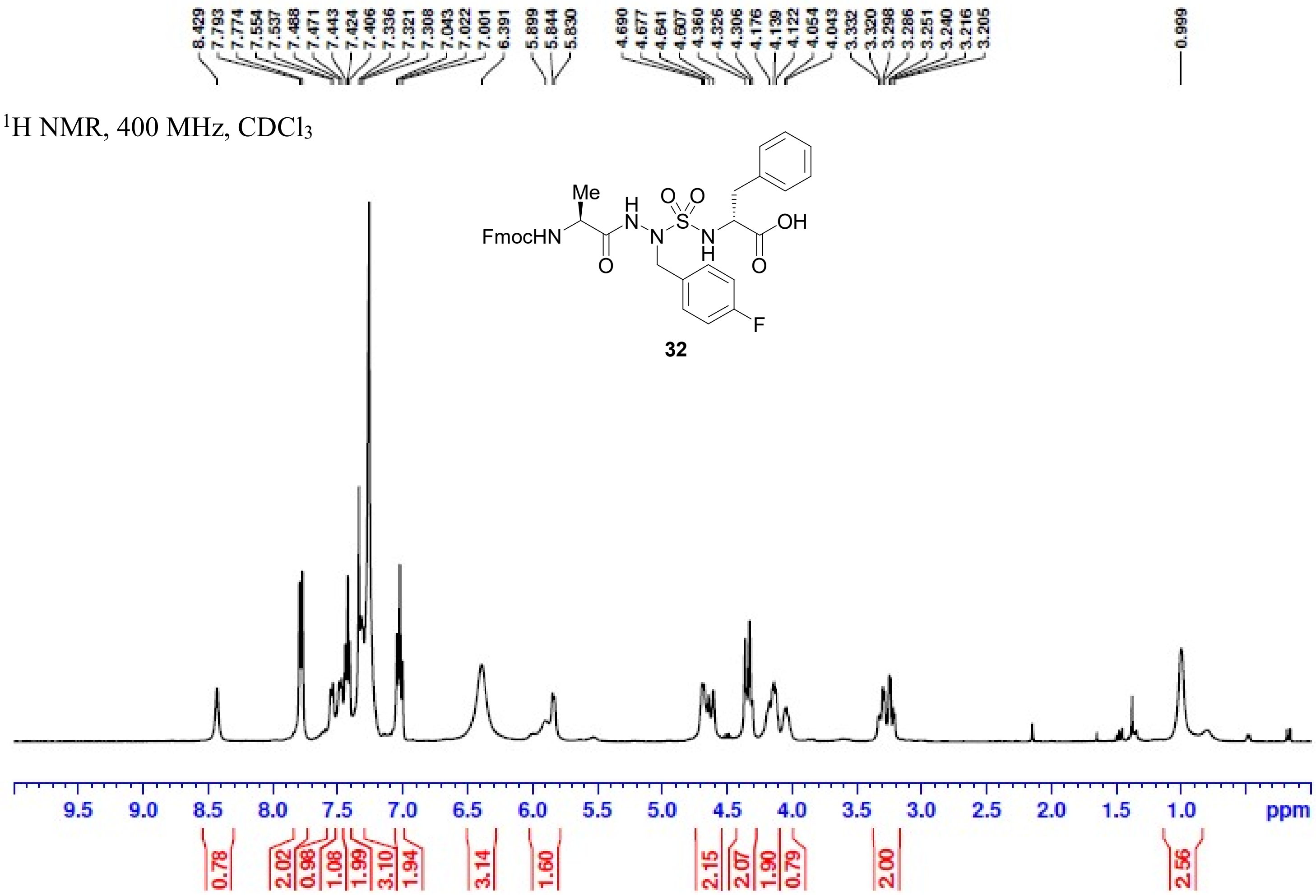

^{1}H NMR, 400 MHz, $CDCl_3$

32

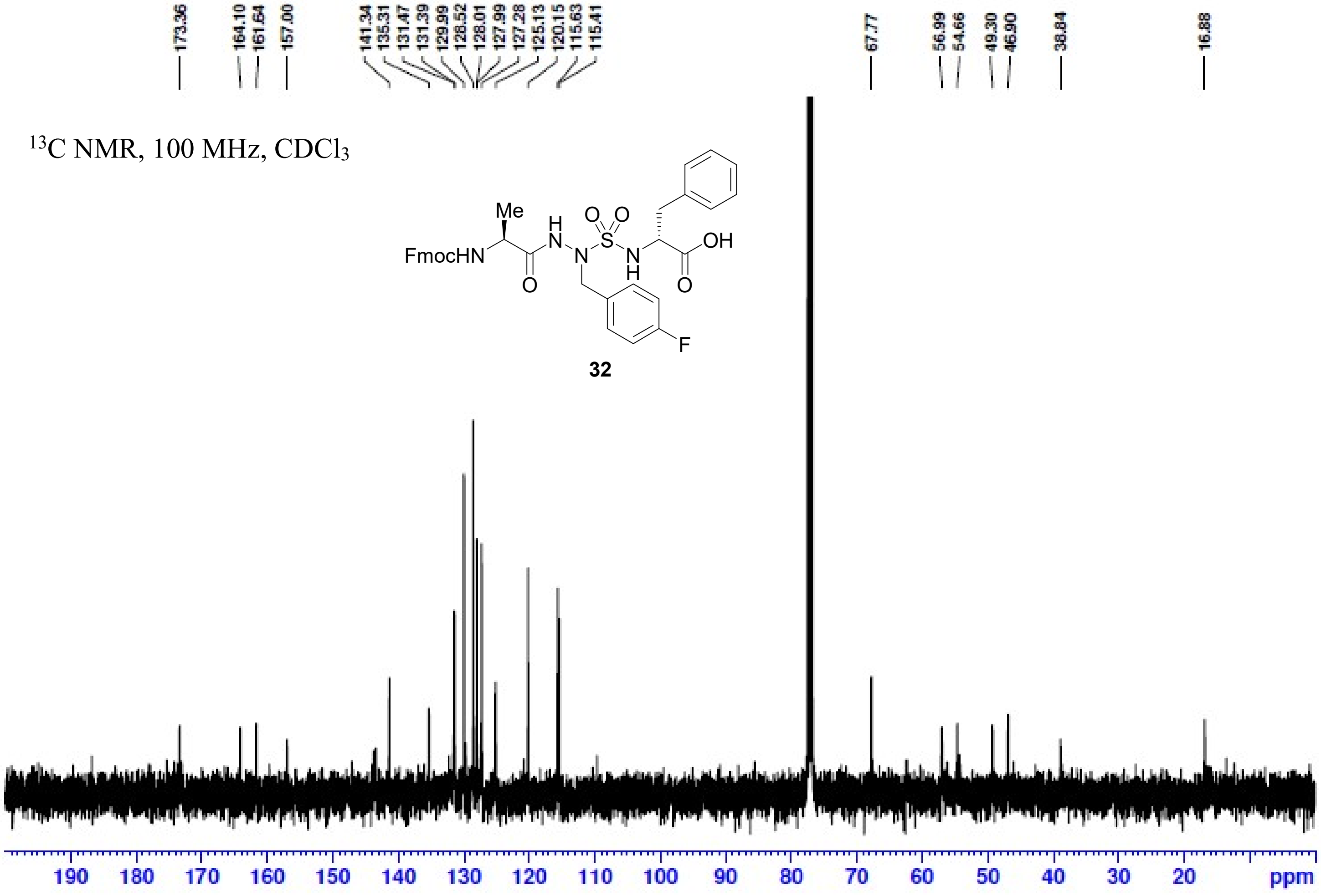

173.36
164.10
161.64
157.00
141.34
135.31
131.47
131.39
129.99
128.52
128.01
127.99
127.28
125.13
120.15
115.63
115.41
67.77
56.99
54.66
49.30
46.90
38.84
16.88
13C NMR, 100 MHz, CDCl3
Me
FmocHN
H
N
O
S
OH
32
F
ppm
190
180
170
160
150
140
130
120
110
100
90
80
70
60
50
40
30
20

D-7000 HPLC System Manager Report

Analyzed: 01/11/10 12:40 PM   Reported: 01/11/10 01:47 PM
Processed: 01/11/10 01:47 PM

Data Path: H:\echantillons\DATA\0246\
Processing Method: Grad 05-80%MeOH 30min(40min)ST
System(acquisition): Sys 1   Series:0246
Application: echantillons   Vial Number: 3
Sample Name: SPST-7-1(P2)_5-80MeOH   Vial Type: UNK
Injection from this vial: 1 of 1   Volume: 5.0 ul
Sample Description: SPST-7-1(P2)_5-80MeOH

Chrom Type: Fixed WL Chromatogram, 214 nm

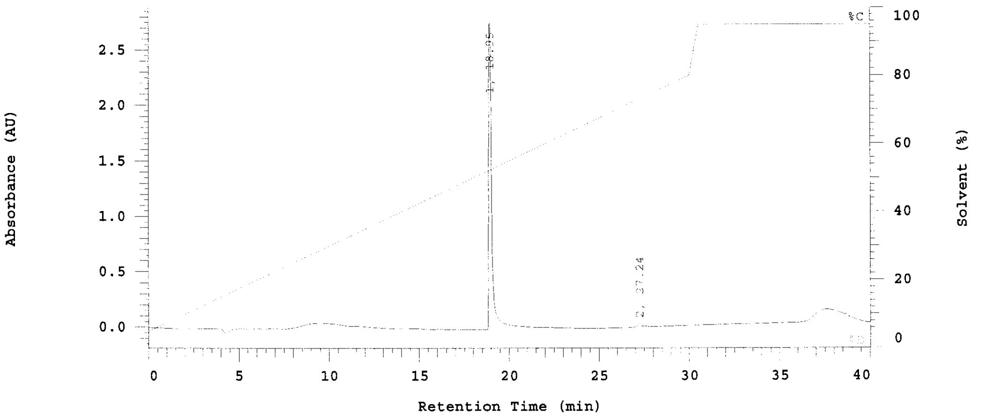

Acquisition Method: Grad 05-80%MeOH 30min(40min)ST
Column Type: Developed by: Lubell
Pump A Type: L-7100
Solvent A: H2O 0.1% FA   Solvent B: MeCN 0.1% FA
Solvent C: MeOH 0.1% FA   Solvent D: Storage H2O/ORG 35/65 no FA
Method Description: grad 5 to 80% MeOH in 30 min, 10 min wash at 95% MeOH

Chrom Type: Fixed WL Chromatogram, 214 nm

Peak Quantitation: AREA
Calculation Method: AREA%

No.	RT	Area	Area %
1	18.95	15485900	98.771
2	27.24	192660	1.229
		15678560	100.000

Peak rejection level: 10000

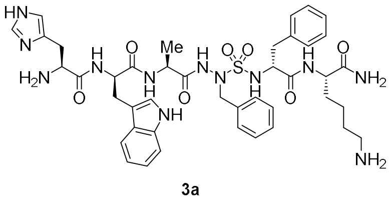

3a

Page Indicator 1 / 1

D-7000 HPLC System Manager Report

Analyzed: 01/11/10 10:55 AM
Reported: 01/11/10 01:48 PM
Processed: 01/11/10 01:45 PM

Data Path: H:\echantillons\DATA\0244\
Processing Method: Grad 05-80%MeCN 30min(40min)ST

System(acquisition): Sys 1
Series:0244
Application: echantillons
Vial Number: 3
Sample Name: SPST-7-1(P2)_5-80MeCN
Vial Type: UNK
Injection from this vial: 1 of 1
Volume: 5.0 ul
Sample Description: SPST-7-1(P2)_5-80MeCN

Chrom Type: Fixed WL Chromatogram, 214 nm

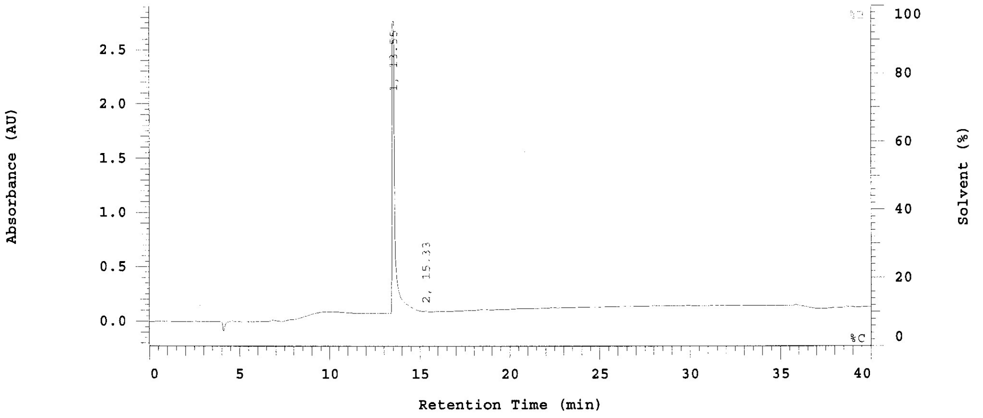

Acquisition Method: Grad 05-80%MeCN 30min(40min)ST
Column Type:
Developed by: Lubell
Pump A Type: L-7100
Solvent A: H2O 0.1% FA
Solvent B: MeCN 0.1% FA
Solvent C: MeOH 0.1% FA
Solvent D: Storage H2O/ORG 35/65 no FA
Method Description: grad 5 to 80% MeCN in 30 min, 10 min wash at 95% MeCN

Chrom Type: Fixed WL Chromatogram, 214 nm

Peak Quantitation: AREA
Calculation Method: AREA%

No.	RT	Area	Area %
1	13.55	16142720	99.809
2	15.33	30920	0.191
		16173640	100.000

Peak rejection level: 10000

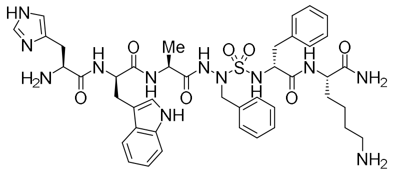

3a

Page Indicator 1 / 1

Data File C:\CHEM32\1\DATA\2013OCT30\ST0174_(PK15-45MEOH).D
Sample Name: ST0174_(PK15-45MeOH)

===

Acq. Operator : carine
Acq. Instrument : Instrument 1
Injection Date : 29/10/2013 12:50:59 PM

Seq. Line : 17
Location : P2-A-05
Inj : 1
Inj Volume : 5 µl

Different Inj Volume from Sequence ! Actual Inj Volume : 3 µl

Acq. Method : C:\CHEM32\1\METHODS\LC_15_45_12MN_MEOH_
Last changed : 23/10/2013 9:42:49 AM
Analysis Method : C:\CHEM32\1\METHODS\CONDITIONNEMENT_15ACN_5 MIN.M
Last changed : 29/10/2013 2:22:35 PM by carine
(modified after loading)

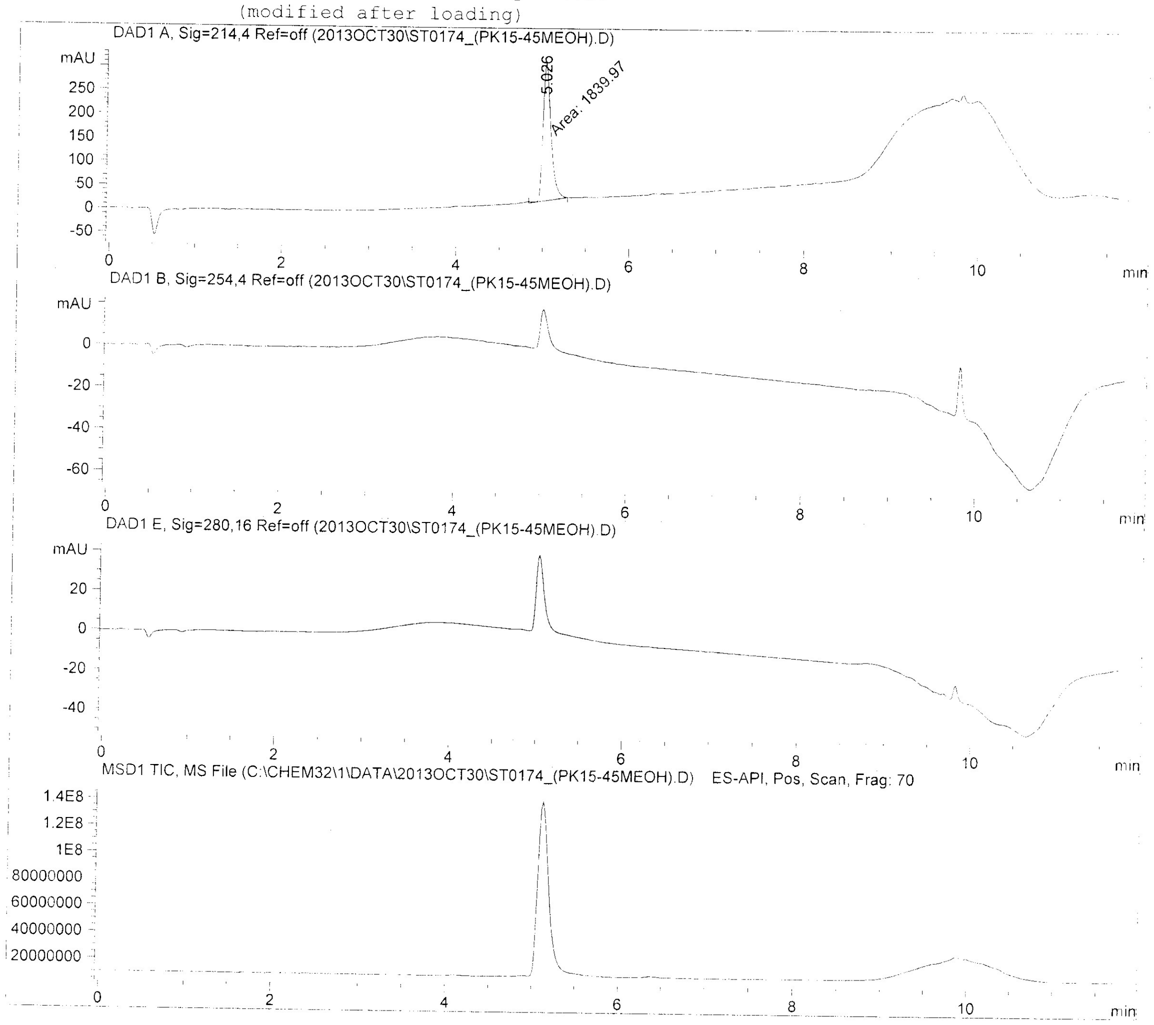

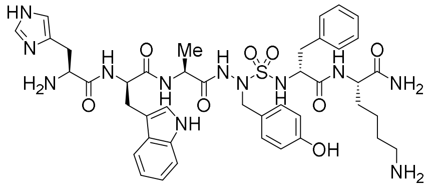

3b

Data File C:\CHEM32\1\DATA\2013OCT30\ST0174_(PK15-40ACN).D
Sample Name: ST0174_(PK15-40ACN)

===

Acq. Operator : carine Seq. Line : 2
Acq. Instrument : Instrument 1 Location : P2-A-05
Injection Date : 29/10/2013 2:31:25 PM Inj : 1
Inj Volume : 5 µl
Different Inj Volume from Sequence ! Actual Inj Volume : 3 µl
Acq. Method : C:\CHEM32\1\METHODS\LC_15_40_12MN_ACN_S
Last changed : 29/10/2013 12:04:55 PM by carine
Analysis Method : C:\CHEM32\1\METHODS\CONDITIONNEMENT_05ACN_5 MIN.M
Last changed : 30/10/2013 9:47:32 AM by carine
(modified after loading)

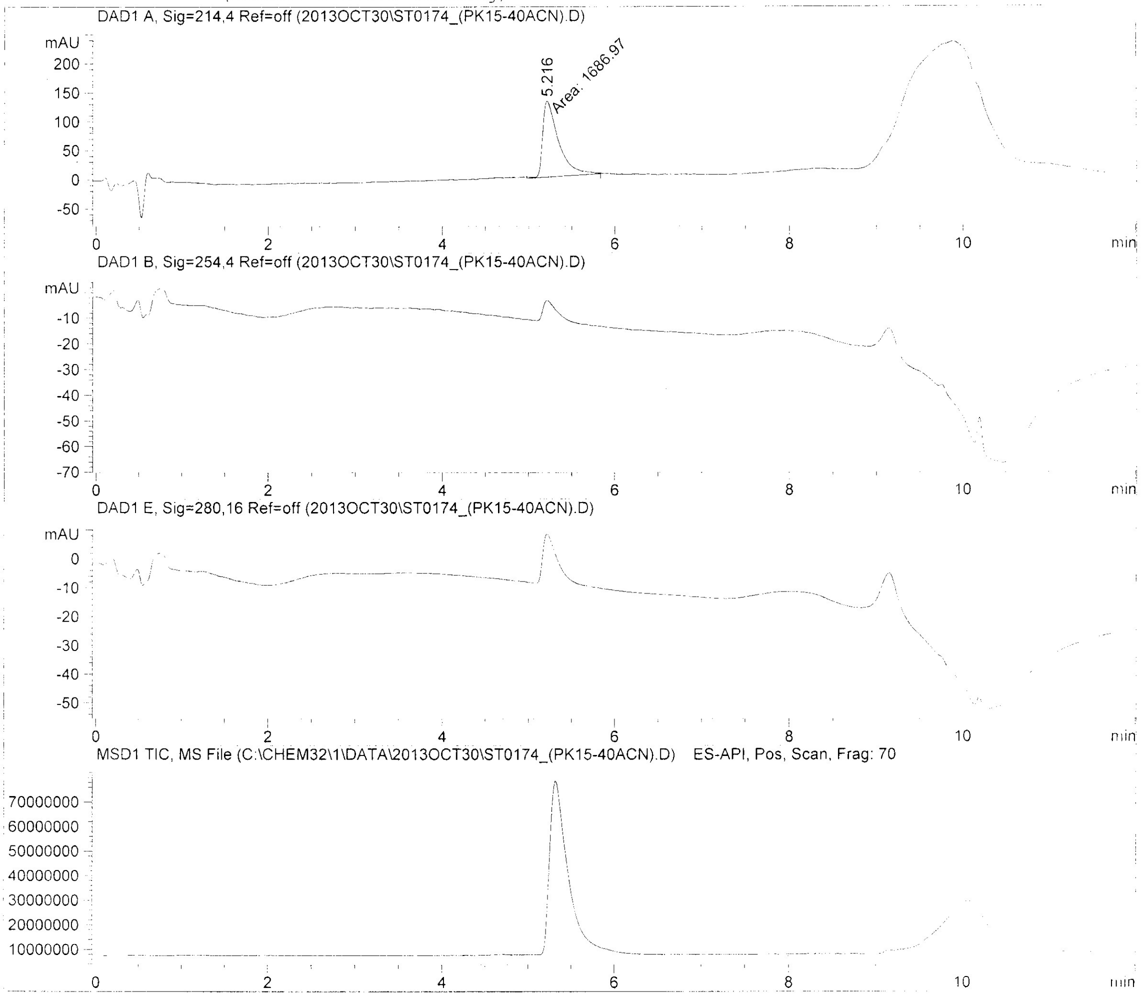

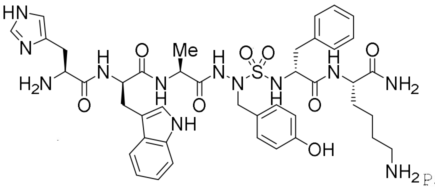

3b

Print of window 38: Current Chromatogram(s)

Current Chromatogram(s)

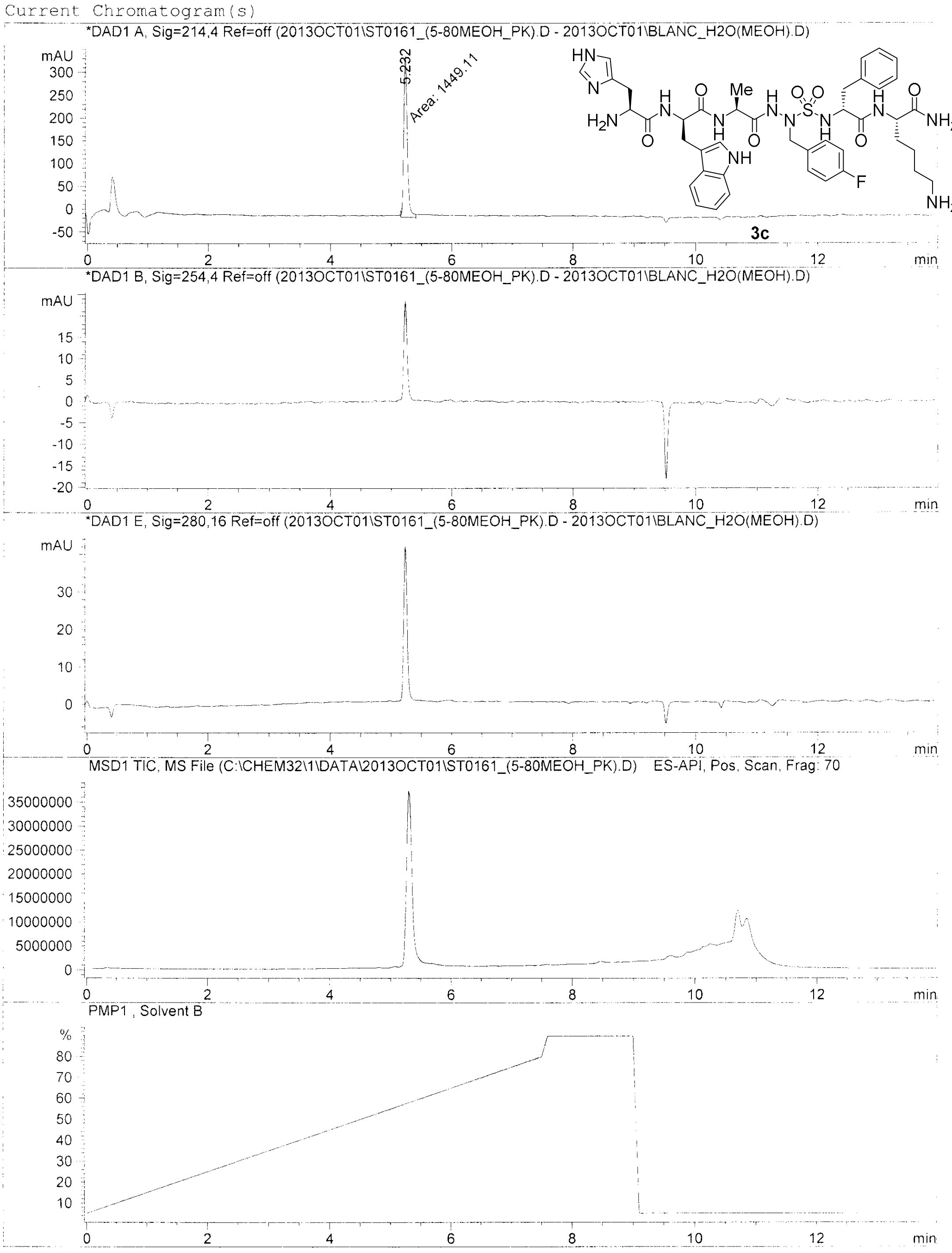

Instrument 1 04/10/2013 3:10:53 PM

Page 1 of 1

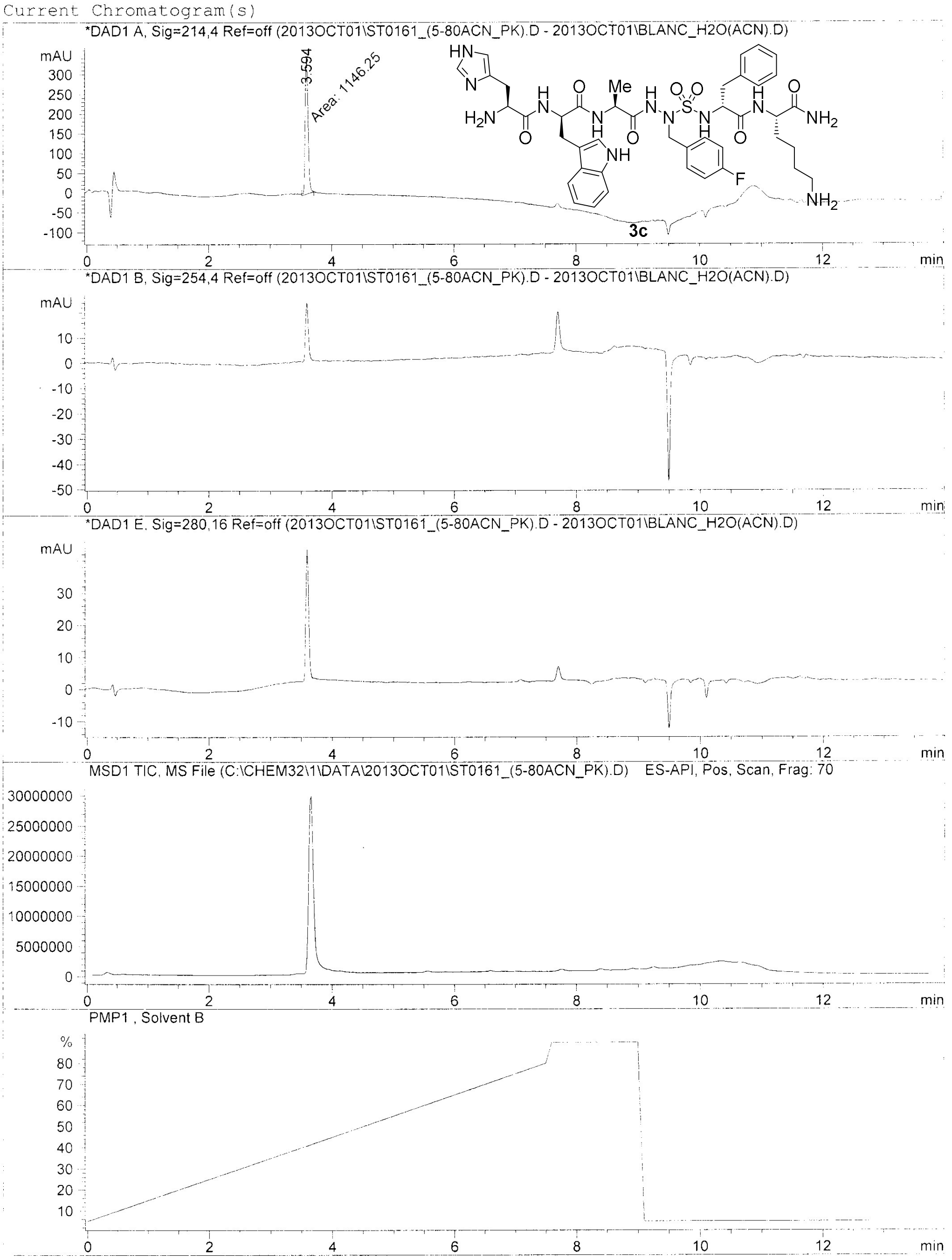
Current Chromatogram(s)
*DAD1 A, Sig=214,4 Ref=off (2013OCT01\ST0161_(5-80ACN_PK).D - 2013OCT01\BLANC_H2O(ACN).D)
mAU
3.594
Area: 1146.25
3c
min
*DAD1 B, Sig=254,4 Ref=off (2013OCT01\ST0161_(5-80ACN_PK).D - 2013OCT01\BLANC_H2O(ACN).D)
*DAD1 E, Sig=280,16 Ref=off (2013OCT01\ST0161_(5-80ACN_PK).D - 2013OCT01\BLANC_H2O(ACN).D)
MSD1 TIC, MS File (C:\CHEM32\1\DATA\2013OCT01\ST0161_(5-80ACN_PK).D) ES-API, Pos, Scan, Frag: 70
PMP1 , Solvent B
%

Data File C:\CHEM32\1\DATA\2013OCT21\ST0171_(PK15-45MEOH).D
Sample Name: ST0171_(PK15-45MeOH)

===

Acq. Operator : Seq. Line : 19
Acq. Instrument : Instrument 1 Location : P2-A-01
Injection Date : 21/10/2013 3:45:41 PM Inj : 1
Inj Volume : 5 µl
Acq. Method : C:\CHEM32\1\METHODS\LC_15_45_14MN_MEOH_
Last changed : 08/10/2013 3:38:45 PM
Analysis Method : C:\CHEM32\1\METHODS\LC_20_90_12MN_MEOH_CB.M
Last changed : 22/10/2013 9:56:50 AM
(modified after loading)

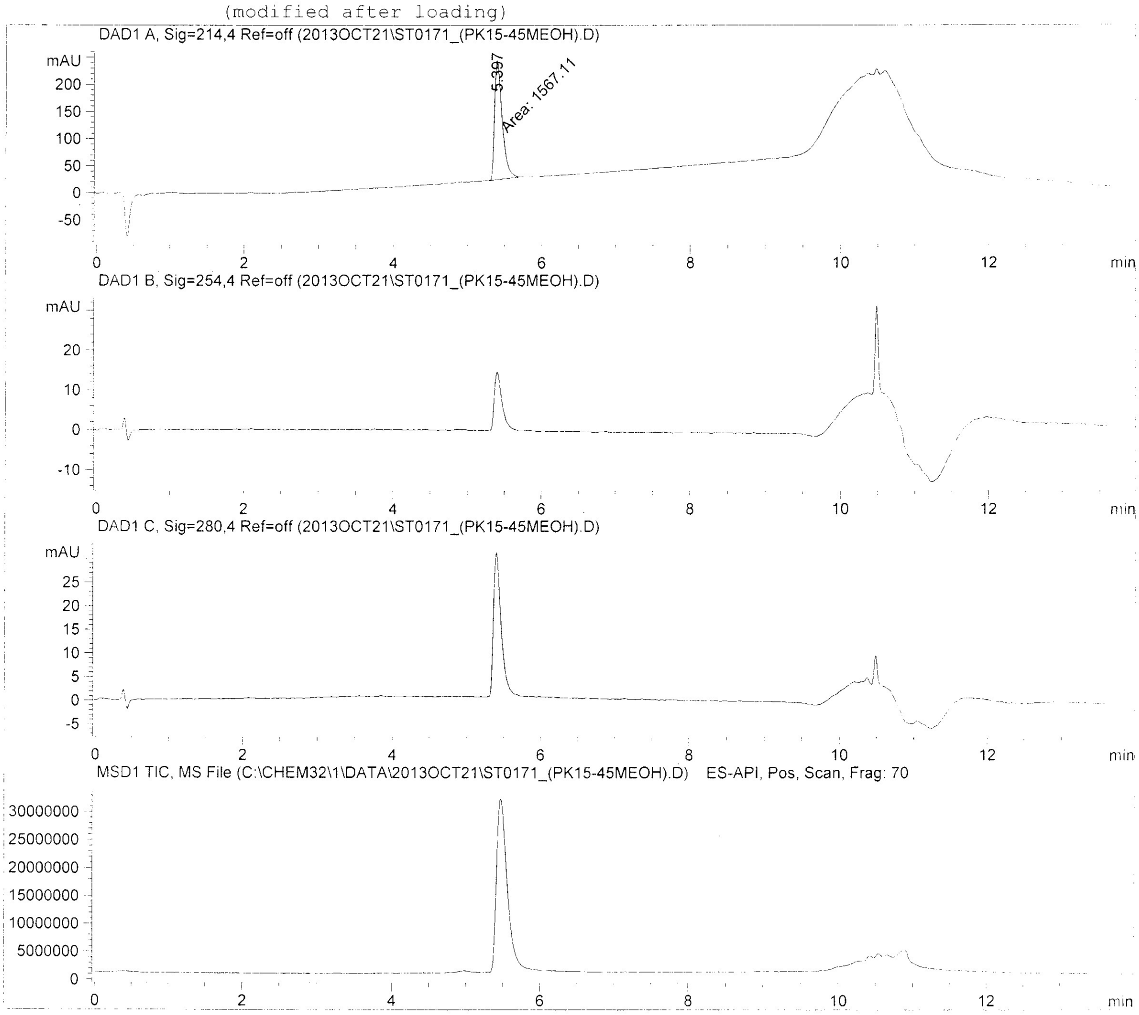

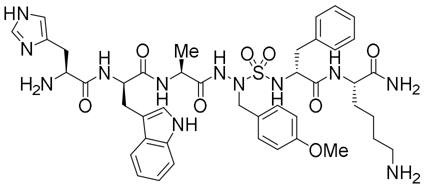

3d

Data File C:\CHEM32\1\DATA\2013OCT21\ST0171_(PK10-30ACN).D
Sample Name: ST0171_(PK10-30ACN)

===

Acq. Operator	:	Seq. Line :	24
Acq. Instrument	: Instrument 1	Location :	P2-A-01
Injection Date	: 21/10/2013 4:47:23 PM	Inj :	1
		Inj Volume :	5 µl

Acq. Method : C:\CHEM32\1\METHODS\LC_10_30_14MN_ACN_S
Last changed : 18/10/2013 9:56:58 AM
Analysis Method : C:\CHEM32\1\METHODS\LC_20_90_12MN_MEOH_CB.M
Last changed : 22/10/2013 9:56:50 AM
(modified after loading)

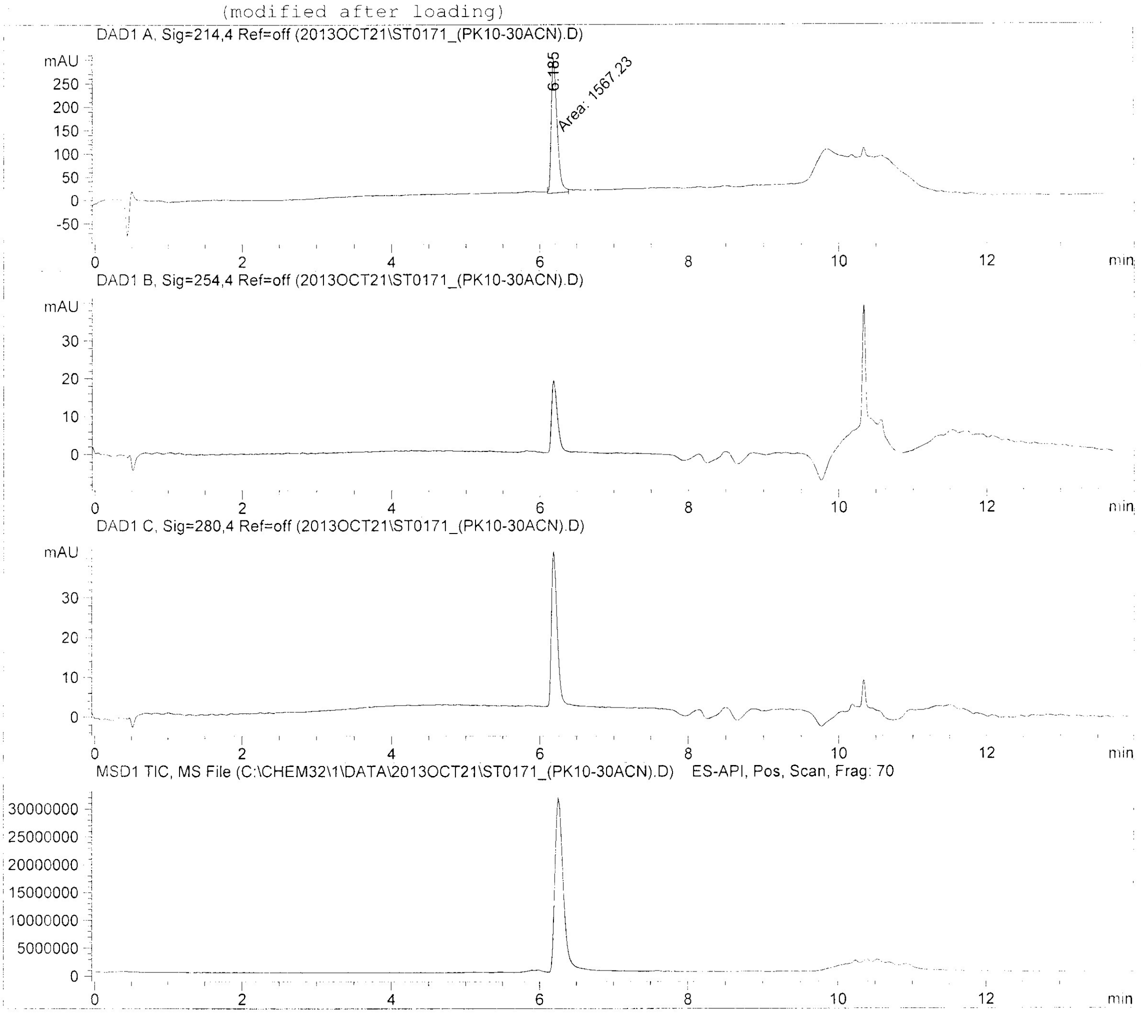

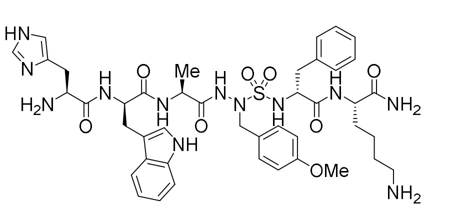

Instrument 1 22/10/2013 9:57:32 AM

3d

Page 1 of 2

```
Data File C:\CHEM32\1\DATA\2013OCT21\ST0172_(PK25-55MEOH).D
Sample Name: ST0172_(PK25-55MeOH)

=====================================================================
Acq. Operator       :                              Seq. Line :  21
Acq. Instrument     : Instrument 1                  Location : P2-A-02
Injection Date      : 21/10/2013 4:09:13 PM              Inj :   1
                                                  Inj Volume : 5 µl
Acq. Method         : C:\CHEM32\1\METHODS\LC_25_55_14MN_MEOH_
Last changed        : 08/10/2013 10:21:32 AM
Analysis Method     : C:\CHEM32\1\METHODS\LC_20_90_12MN_MEOH_CB.M
Last changed        : 22/10/2013 9:56:50 AM
                      (modified after loading)
```

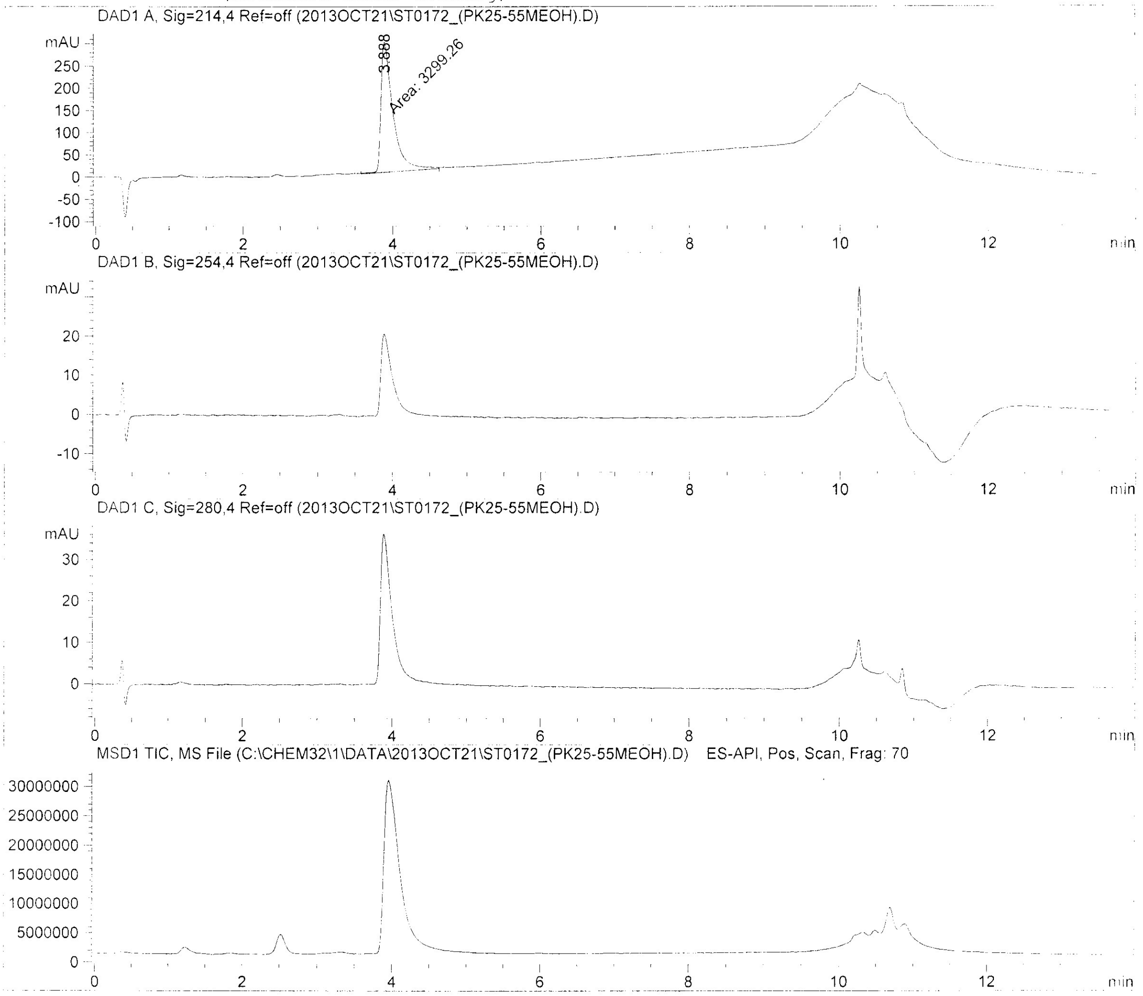

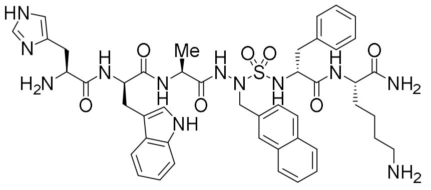

3e

Instrument 1 22/10/2013 10:02:56 AM   Page 1 of 2

Data File C:\CHEM32\1\DATA\2013OCT21\ST0172_(PK15-35ACN).D
Sample Name: ST0172_(PK15-35ACN)

===

Acq. Operator : Seq. Line : 26
Acq. Instrument : Instrument 1 Location : P2-A-02
Injection Date : 21/10/2013 5:09:19 PM Inj : 1
Inj Volume : 5 µl
Acq. Method : C:\CHEM32\1\METHODS\LC_15_35_14MN_ACN_S
Last changed : 21/10/2013 11:25:10 AM
Analysis Method : C:\CHEM32\1\METHODS\LC_20_90_12MN_MEOH_CB.M
Last changed : 22/10/2013 9:56:50 AM
(modified after loading)

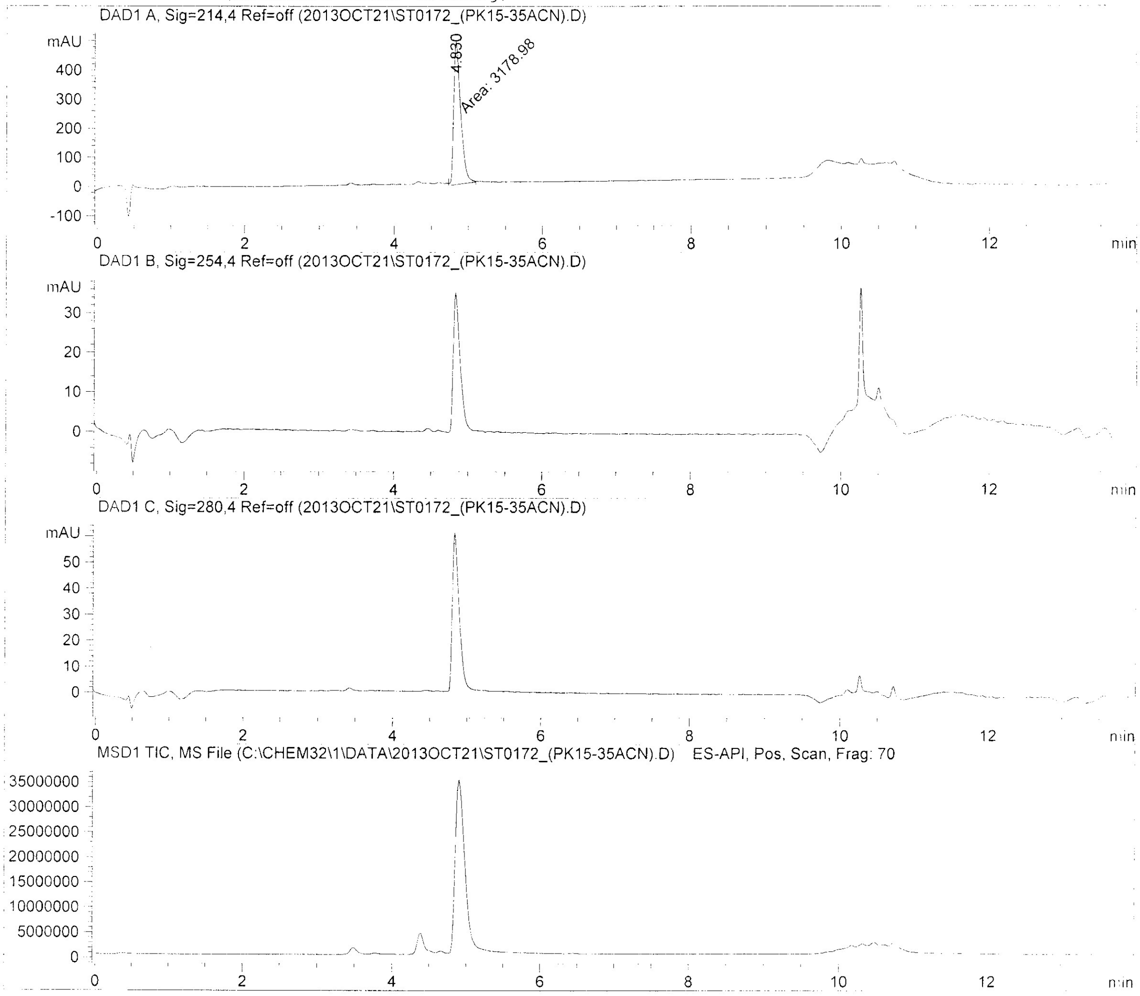

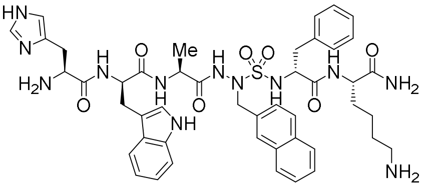

3e

Instrument 1 22/10/2013 10:02:31 AM Page 1 of 2

Data File name: C:\CHEM32\1\DATA\2014AUG13\WDL-LDY-88-LC_20_80_14MN_MEOH_ST->
Date:13 July 2014
Acq method: C:\CHEM32\1\METHODS\LC_20_80_14MN_MEOH_

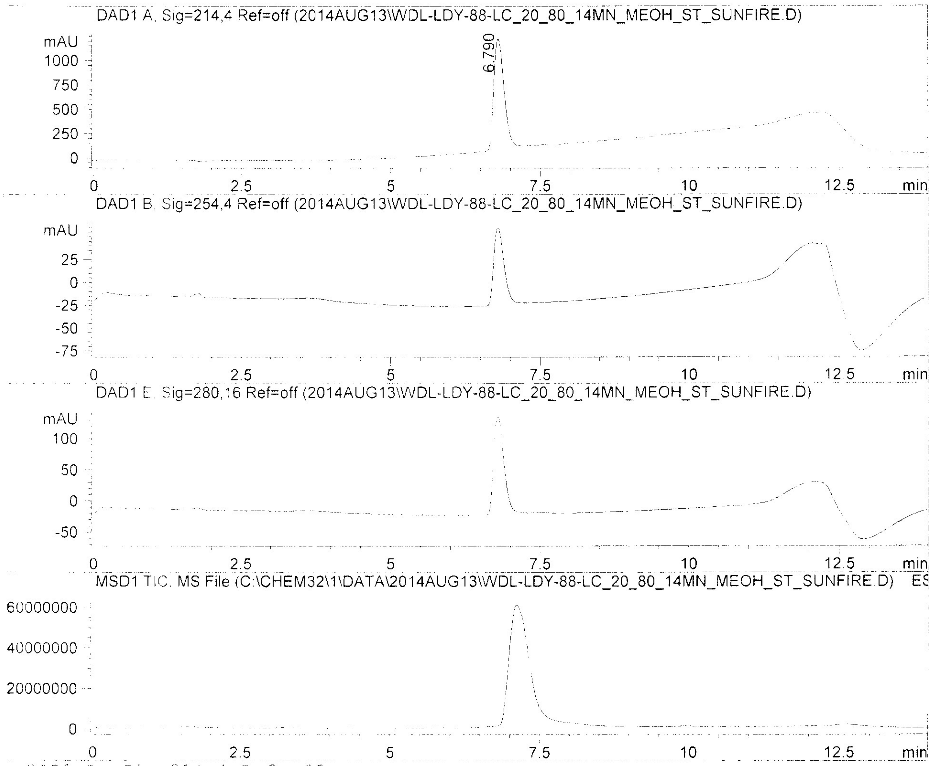

DAD1 A, Sig=214,4 Ref=off

Ret. Time	Height	Area	Area %
6.790	1140.937	13941.933	100.000

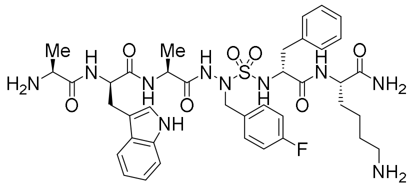

3f

DAD1 B, Sig=254,4 Ref=off

Ret. Time	Height	Area	Area %

DAD1 E, Sig=280,16 Ref=off

Ret. Time	Height	Area	Area %

Page 1 of 2

Data File name: C:\CHEM32\1\DATA\2014AUG13\WDL-LDY-84-10-80-ACN-14MIN.D
Date:13 July 2014
Acq method: C:\CHEM32\1\METHODS\LC_10_80_14MN_ACN_S

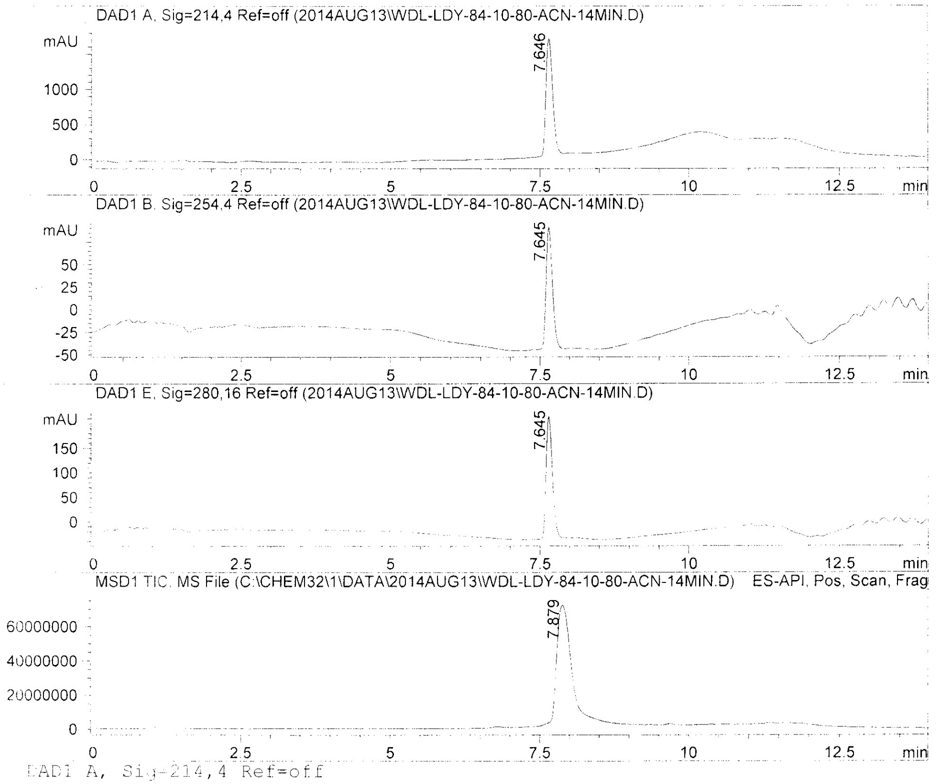

DAD1 A, Sig=214,4 Ref=off

Ret. Time	Height	Area	Area %
7.646	1653.223	12945.865	100.000

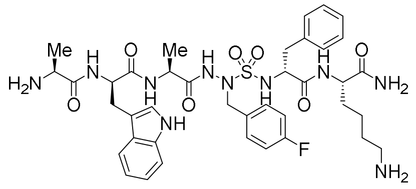

3f

DAD1 B, Sig=254,4 Ref=off

Ret. Time	Height	Area	Area %
7.645	132.671	956.554	100.000

DAD1 E, Sig=280,16 Ref=off

Ret. Time	Height	Area	Area %
7.645	252.605	1929.010	100.000

Page 1 of 2
